# Transcriptome changes in leukocytes of dairy calves exposed to heat stress

**DOI:** 10.1093/tas/txag029

**Published:** 2026-03-15

**Authors:** Sonia J Moisá, Trevor Freeman, Jonathan E Beever, Zhantao Yu, Juan M Cantet, M R R Nair, Agustín G Ríus

**Affiliations:** Department of Animal Science, University of Tennessee Institute of Agriculture, Knoxville, TN, 37996, United States; Department of Animal Science, University of Tennessee Institute of Agriculture, Knoxville, TN, 37996, United States; Department of Animal Science, University of Tennessee Institute of Agriculture, Knoxville, TN, 37996, United States; Department of Animal Science, University of Tennessee Institute of Agriculture, Knoxville, TN, 37996, United States; Department of Animal Science, University of Tennessee Institute of Agriculture, Knoxville, TN, 37996, United States; Department of Animal Science, University of Tennessee Institute of Agriculture, Knoxville, TN, 37996, United States; Department of Animal Science, University of Tennessee Institute of Agriculture, Knoxville, TN, 37996, United States

## Abstract

Heat stress impacts the health and immune function of dairy calves. Our objective was to conduct a longitudinal study to assess temporal alterations in the transcriptome of white blood cells from dairy calves exposed to heat stress. Eight Holstein bull calves (68.5 ± 1.37 kg of BW, aged 3.5 ± 0.5 weeks) were raised in individual pens following current commercial management practices. Calves were exposed to cyclic heat stress treatment (40°C ambient temperature from 0800 to 1900 h daily followed by 27°C) for 5 days. Blood samples were collected at 0, 1, and 5 days relative to treatment to conduct RNA extractions from white blood cells for RNA sequencing. Comparisons between control on day 0 against day 1 and day 5 of differentially expressed genes (DEGs) were considered using an adjusted P-value < 0.05. A total of 5 DEGs were detected when comparing control day 0 against day 1 (i.e., 3 downregulated and 2 upregulated). A total of 1,133 DEGs were detected when comparing control day 0 against day 5 (i.e., 722 downregulated and 411 upregulated). Differentially expressed genes were screened to fit into functional categories of pathways or ontologies associated with known impacts on functions. The functional annotation of differentially expressed genes showed upregulation of pathways associated with the heat shock response and changes in lipid metabolism possibly to support the activation of immune cells on day 1 compared with day 0. Transcriptional changes showed downregulation of “Spliceosome,” “Neutrophil extracellular trap formation,” and “ATP-dependent chromatin remodeling” on day 5 compared with day 0 due to the inhibition of most of spliceosomal RNA-related genes and several clustered histone variants respectively. In general, on day 1 heat stress was associated with preservation of cellular homeostasis and changes in metabolism. On day 5 heat stress was associated with the inhibition of various pathways, possibly to tightly control inflammation and prevent dysregulation of leukocytes’ function.

## Teaser

Our results provide clues, at the transcriptome level, concerning early and late leukocyte response to heat stress in dairy calves.

## Graphical Abstract

**Graphical Abstract:**
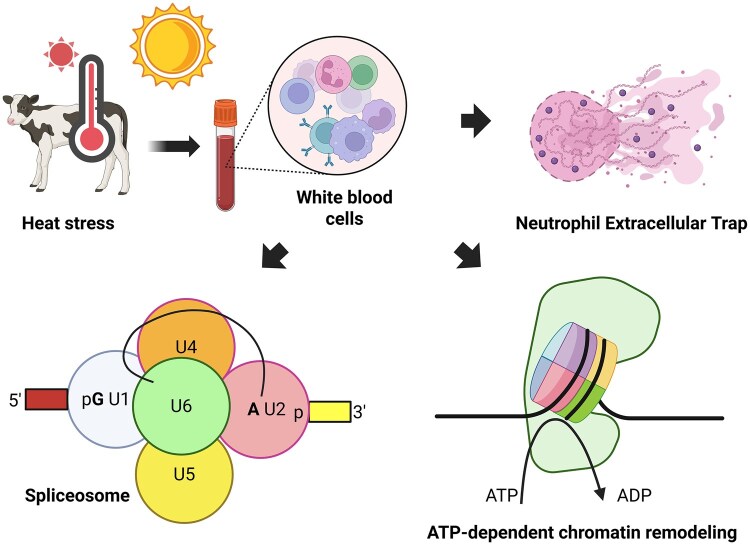

## Introduction

Heat stress occurs when exposure to high environmental temperature and humidity results in disruption of thermoneutrality, and animals are not able to dissipate heat effectively. As a result, animal productivity and health decline and significant economic losses occur in the livestock industry (Kotsampasi et al. 2024). Existing heat abatement tools such as ventilation and spray cooling are used to mitigate the effects of elevated ambient temperatures on dairy farms. These tools partially control the heat stress impact on lactating cows; however, modern heat abatement tools are not used to control heat stress in calves in most dairy farms (Wang et al. 2020; Kaufman et al. 2021). Furthermore, the efficacy of cooling systems is influenced by several animal-related factors (eg genomics, hair coat, and sweat gland characteristics and numbers). For example, in Holstein cows identified as high-immune responders, in vitro heat stress of peripheral mononuclear cells triggered differentially methylated regions in the DNA of genes associated with stress response and prevention of apoptosis. However in low-immune responder cows, heat stress resulted in differentially methylated regions of genes associated with cell proliferation and histone deacetylases (Livernois et al. 2021). This suggests that the genomic response to heat stress is significantly affected by the animal underlying immune phenotype.

Gene networks within and across cells respond to heat stress with intracellular signals that coordinate cellular and whole-animal metabolism (Wu et al. 2020). Changes in gene expression regulate key biological processes to protect cells from the adverse effects of heat stress and maintain tissues’ homeostasis (Jianfang et al. 2025). Previous studies showed transcriptional changes in peripheral white blood cells of lactating dairy cows exposed to heat stress. The analysis showed changes related to lipid and carbohydrate metabolism, protein folding and refolding, protein phosphorylation, transcription factor binding, immune effector process, cell proliferation, autophagy, and apoptosis, among others (Fang et al. 2021). These results support the concept that the study of animal-related factors such as the transcriptome has the potential to increase our understanding of heat stress impact on growing calves and to develop effective and viable interventions to apply on-farm.

Our previous work revealed that heat stress elicited an increase in intestinal permeability and inflammation that was paralleled with changes in biomarkers of systemic inflammation (Ríus et al. 2022; Yu et al. 2024). However, the role of leukocytes in the heat stress phenotype has not been studied in dairy calves. Leukocytes are a well-described cell model and can be easily collected to determine temporal changes in immune and stress pathways associated with heat stress, while reflecting the overall physiological response of the calf. Therefore, the objective of this study was to assess the changes in gene expression and pathways in white blood cells of dairy calves exposed to heat stress. We hypothesized that heat stress in dairy calves would elicit temporal changes in the transcriptome of white blood cells and reveal key functional pathways related to systemic or tissue specific pro-inflammatory changes (Ríus et al. 2022; Yu et al. 2024).

## Materials and methods

All procedures outlined in this study received approval from the University of Tennessee Institutional Animal Care and Use Committee (IACUC), under protocol number 2851-0921.

### Animal housing and management

Eight newborn Holstein bull calves (68.5 ± 1.37 kg of BW, aged 3.5 ± 0.5 weeks, mean ± SD) gathered at the East Tennessee Ag Research and Education Center (Little River Animal and Environmental Unit, Walland, TN) were raised in individual pens following current commercial management practices. Fresh water and a commercial calf starter were provided ad libitum once daily. A commercial milk replacer was fed to each calf using commercial bottles at 0530 and 1600 h (0.34 kg of milk replacer per feeding). The health of calves was monitored for 2 weeks (data not shown) before transportation to a climate-controlled room in the Johnson Research and Teaching Unit (East Tennessee Research and Education Center, Knoxville, TN) to conduct the study. Two cohorts of 4 animals were transported to a climate-controlled room. The animals had a 5-d adaptation to confinement facilities. After the adaptation period, calves were exposed to cyclic heat stress treatment (40°C ambient temperature from 0800 to 1900 h daily followed by 27°C, ∼12 h/d of heat stress) for 5 days. Climate-controlled room temperature and relative humidity records were monitored every 10 min using loggers located in the front and back of the room at the animal level (HOBO U23 Pro v2; Onset Computer Corp., Bourne, MA; accuracy ± 0.21°C and 2.5% relative humidity). Rectal temperature (RT; GLA M700 digital thermometer; accuracy ± 0.1°C) and respiratory rate (RR, breaths per minute) were measured at 0630, 1100, 1400, 1700, and 1900 h daily in all calves. Personnel were trained to monitor the RR, utilizing the method of quantifying the movement of the flank area within a 15-s timeframe and multiplying by 4.

**Figure 1:**
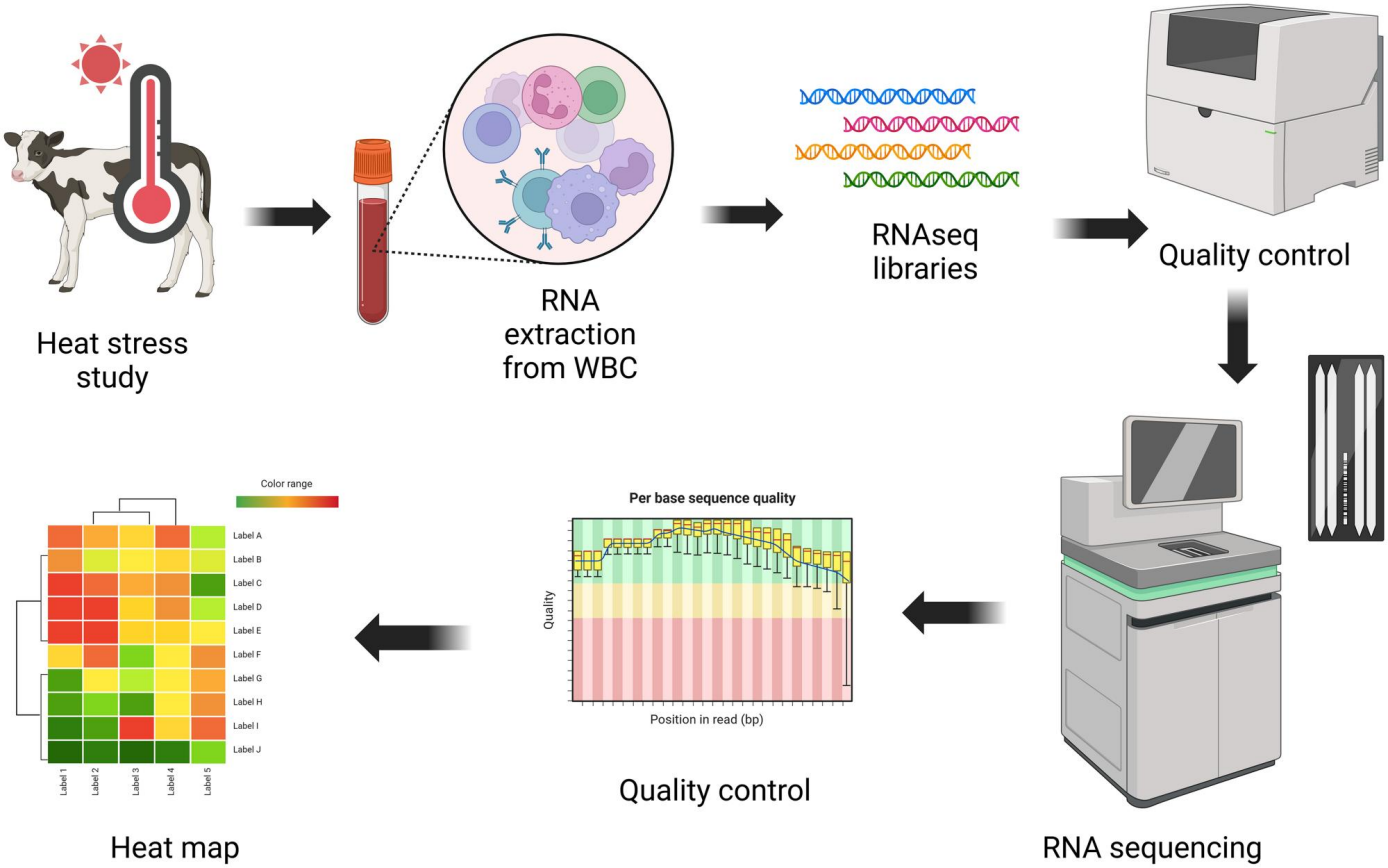
Transcriptomics analysis steps performed in white blood cells (WBC) from dairy calves exposed to heat stress for 5 days.

Blood samples were collected by jugular venipuncture into vacutainers containing K_2_EDTA (dipotassium ethylenediaminetetraacetic acid), from each calf repeatedly at days 0 (baseline control at 0600 h), 1 (at 1800 h), and 5 (at 1800 h) relative to the start of treatment. The 1800 h on day 1 timepoint was selected to capture the initial response to moderate hyperthermia in the transcriptome of white blood cells. It was anticipated that the calves should display changes in physiology associated with hyperthermia on day 1 between noon and 1400 h, thus the 1800 h timepoint aimed to capture early changes in the transcriptome associated with immune function, immune metabolism, and the HSR. Our previous work in dairy calves revealed that heat stress elicited an increase in intestinal permeability and inflammation paralleled with an increase in biomarkers of systemic inflammation (Ríus et al. 2022; Yu et al. 2024). Day 5 at 1800 h timepoint was selected to capture long-term effects of hyperthermia in the transcriptome of white blood cells. Blood samples were placed immediately on ice and processed to extract RNA. An outline of our transcriptomics analysis steps can be found in Fig. 1.

### RNA extraction from white blood cells

A 10X solution of ammonium chloride (NH_4_Cl) red blood cells (RBC) lysis buffer was prepared by adding 83 g ammonium chloride, 10 g of potassium bicarbonate and 40 mL of ½ molar EDTA and 960 mL with purified water (i.e., MilliQ water). One hundred mL of the 10X NH_4_Cl were mixed on 900 mL of purified water to make a 1X NH_4_Cl RBC lysis buffer solution. Then, in a 50 mL RNAse free tube, 10 mL of whole blood were combined with 30 mL of 1X NH_4_Cl RBC lysis buffer. After centrifugation at 2000 × g at room temperature for 10 min and aspiration of the supernatant, this step was repeated until the solution was relatively clear. White blood cells pellet was resuspended with 1.2 mL of Trizol reagent, and the pellet was dissolved by pipetting. In addition, 1 mL of sample was transferred into a 1.5 mL tube and 200 µL of Chloroform were added and mixture was vortexed and shaken vigorously 2 times for about 30 seconds. After a centrifugation step at 18,000 × g and 10°C for 5 min, the upper phase was transferred to a new 1.5 mL tube with 500 µL of Isopropanol (2-propanol). After vortexing each tube and another centrifugation step of 5 min at 18,000 × g and 10°C, the liquid inside the tube was discarded making sure not to lose the RNA pellet while inverting the tube. To wash the RNA pellet, 1000 µL of 75% Ethanol solution were added to the tube and centrifuged again for 5 min at 18,000 × g and 10°C. After centrifugation, the liquid on the tube was discarded by inverting the tube and the remining alcohol was absorbed by a napkin wrapped in a forceps. When the RNA pellet was dry, a solution of RNA inhibitor and RNAse free water (i.e., 1:40 ratio) was used to elute the RNA pellet. The final volume selected to elute the RNA pellet was determined using the size of the RNA pellet. Finally, tubes were vortexed gently at medium speed to resuspend the pellet and the RNA concentration was measured using Nanodrop One C (Thermo Fisher Scientific, Wilmington, DE). The RNA clean and concentrator ^TM^-5 kit was used to remove any DNA contamination by performing the DNAse I treatment before RNA cleanup (Zymo Research Cat #R1014). The quality of the extracted RNA was measured in a Tapestation 4200 (Agilent, Santa Clara, CA). The RNA samples presented an average RNA integrity number (RIN) of 8.7. White blood cells harvested from two calves (ie days 0 and 1) yielded poor quality RNA (i.e., RIN < 7) and data from these samples were not included in the transcriptome analysis.

### Library preparation and sequencing

Sequencing libraries were prepared from isolated RNA using a Takara SMART-Seq v4 3′ DE as per the manufacturer’s instructions. Final library quality and concentrations for pooling were evaluated using the Agilent Tapestation 4200 system. For each kit, libraries for all samples were pooled to achieve equal concentration. Each sample had 5 ng of cDNA. Libraries were sequenced on a single SP flow cell on the Illumina Novaseq 6000 (University of Tennessee Genomics Core—Knoxville, TN) with a 200-cycle v1.5 reagent kit. Read 1 was 150 bp and Read 2 consisted of 26 bp for demultiplexing.

### RNA sequencing data processing

Only forward (R1) reads were used as input for data processing since reverse (R2) reads from the SMART-Seq v4 3’ DE Kit are not informative beyond indexing. Raw reads were trimmed and quality filtered using fastp (v0.23.4; default arguments) (Chen et al. 2018; Chen, 2023). Raw and trimmed reads quality were assessed with FastQC (v0.11.9; default arguments) (Andrews, 2010). Trimmed reads were mapped to the *Bos taurus* reference genome ARS-UCD2.0 (NCBI RefSeq GCF_002263795.3) using STAR (2.7.11b; arguments: ‘–outSAMtype BAM Unsorted’) (Dobin et al. 2013). Quantification was performed with SubRead featureCounts (v2.0.6; default arguments) (Liao et al. 2014). Quality control metrics were collected at each step and summarized in a MultiQC (1.21) report to evaluate overall library quality and the performance of the processing steps (Ewels et al. 2016). Information about read counts, mapping rates, and quality metrics is provided in Additional Table S10.

### RNA sequencing statistical analysis

Statistical analysis was performed in R. Heat stress effect was analyzed on days 1 and 5 and compared with control day 0. For libraries sequenced across multiple runs, counts for technical replicates were summed using the collapseReplicates() function from the DESeq2 package (Love et al. 2014). Low expressed genes were filtered out with the filterByExpr() function from the edgeR package with grouping by the group variable and a minimum count of 10 (Robinson et al. 2010). Descriptive statistics visualizations such as principal components analysis, correlation heatmaps, and variance partitioning analysis were produced to evaluate sample quality and aid in model selection (Hoffman and Roussos 2021).

Differentially expressed genes (DEGs) across time points were called using the dream analysis workflow from the variancePartition package (Hoffman and Roussos 2021). Briefly, normalization factors were calculated for counts matrix with edgeR calcNormFactors, and count transformation weights for the linear mixed model were estimated using voomWithDreamWeights. A linear mixed model was fit for each gene weighted by the voom estimates computed in the previous step performed using the dream analysis workflow from the variancePartition package. Subsequently, test statistics were moderated using empirical Bayes moderation as implemented in variancePartition::eBayes. Finally, differentially expressed genes results for all genes from the desired contrasts were extracted with variancePartition::toptable. These results tables were then annotated with each gene’s DEG status–the result of Benjamini-Hochberg adjusted P-value < 0.05—and direction of expression changes for DEGs for coefficients that yielded a t-test result–upregulated for log2 fold change > 0; downregulated for log2 fold change < 0.

A repeated measures study with individuals nested within cohorts was considered using the dream workflow as it allows for linear mixed models to be fit for each gene. In addition to these variables which are best modeled as random effects, different groups had different tissue compositions as exemplified by differences in the white blood cell counts, neutrophil counts, and lymphocyte counts across groups (data not shown and to be published in a companion paper). To control tissue composition effects, the percentage of white blood cells that were neutrophils was included as a model term.

The gprofiler2 R package (Kolberg et al. 2023) was used to convert NCBI gene symbols to Entrez Gene IDs or NCBI gene IDs from the National Center for Biotechnology Information (https://www.ncbi.nlm.nih.gov/gene/).The modification of the gene annotation is a required for proper identification of each gene in the following steps in the analysis.

Functional enrichment analysis was performed with the Database for Annotation, Visualization, and Integrated Discovery (DAVID) (Sherman et al. 2024) using the NCBI gene list and considering a cutoff value of FDR = 0.05. Gene ontology terms (biological processes, cellular components, molecular functions) and integrative protein signature database (INTERPRO) data were obtained using DAVID (Additional Table S4). The Kyoto Encyclopedia of Genes and Genomes (KEGG) maps were utilized as an illustration of the DEGs present in each significant KEGG pathway, which were taken as a reference for our discussion (Additional Tables S5–S9, (Ogata et al. 1999). The dataset analyzed during the current study is available in the NCBI Gene Expression Omnibus https://www.ncbi.nlm.nih.gov/geo/ under accession number GSE 303550.

## Results

**Figure 2:**
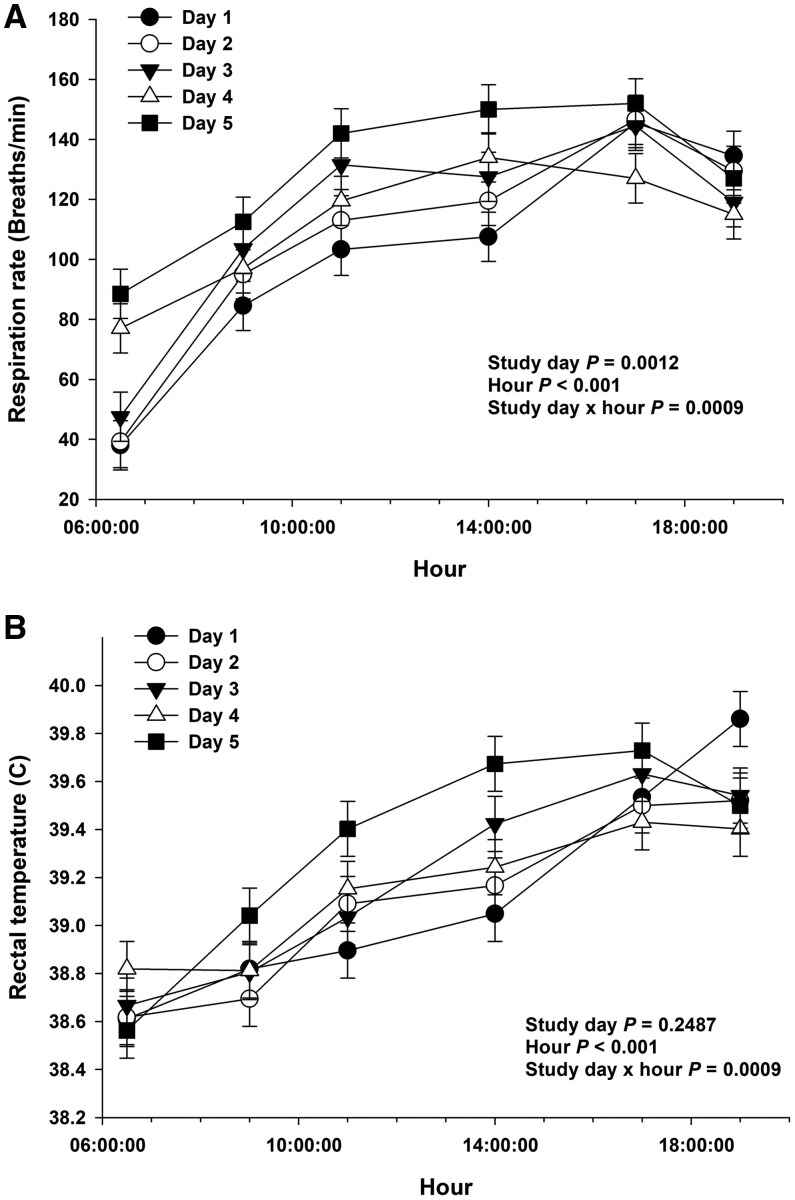
Respiratory rate (A) and rectal temperature (B) in calves exposed to heat stress for 5 days.

The ambient temperature in the climate-controlled room ranged from 19.1°C to 30.9°C between 2000 and 0800 h, from 21.5°C to 40.6°C between 0800 and 1900 h, and the relative humidity ranged from 12.7% to 69.2% (data not shown). By design, RR increased up to 2-fold on 1100, 1400, 1700, and 1900 h compared with 0630 h (Hour *P* < 0.05), and the magnitude of the increase in RR was greater in days 4 and 5 compared with days 1, 2 and 3 on 0630 h (Day x hour interaction *P* < 0.05; Fig. 2A). Rectal temperature increased at 1100, 1400, 1700, and 1900 h compared with 0630 h (Hour *P* < 0.05), and the magnitude of the increase in RT was greater in day 5 compared with day 1 on 1100 and 1400 h (Day × hour interaction *P* < 0.05; Fig. 2B).

Compared with day 0, there were 5 DEGs on day 1 of heat stress (i.e., 3 downregulated and 2 upregulated genes out of 17,059; Additional Table S1). The DEGs that were downregulated included uncharacterized LOC100847791 (*LOC100847791*), chromosome 17 C5orf52 homolog (*C17H5orf52*) and cold inducible RNA binding protein (*CIRBP*). The DEGs that were upregulated included Nocturnin (*NOCT*) and stress induced phosphoprotein 1 (*STIP1*; Additional Tables S1 and S2).

**Figure 3:**
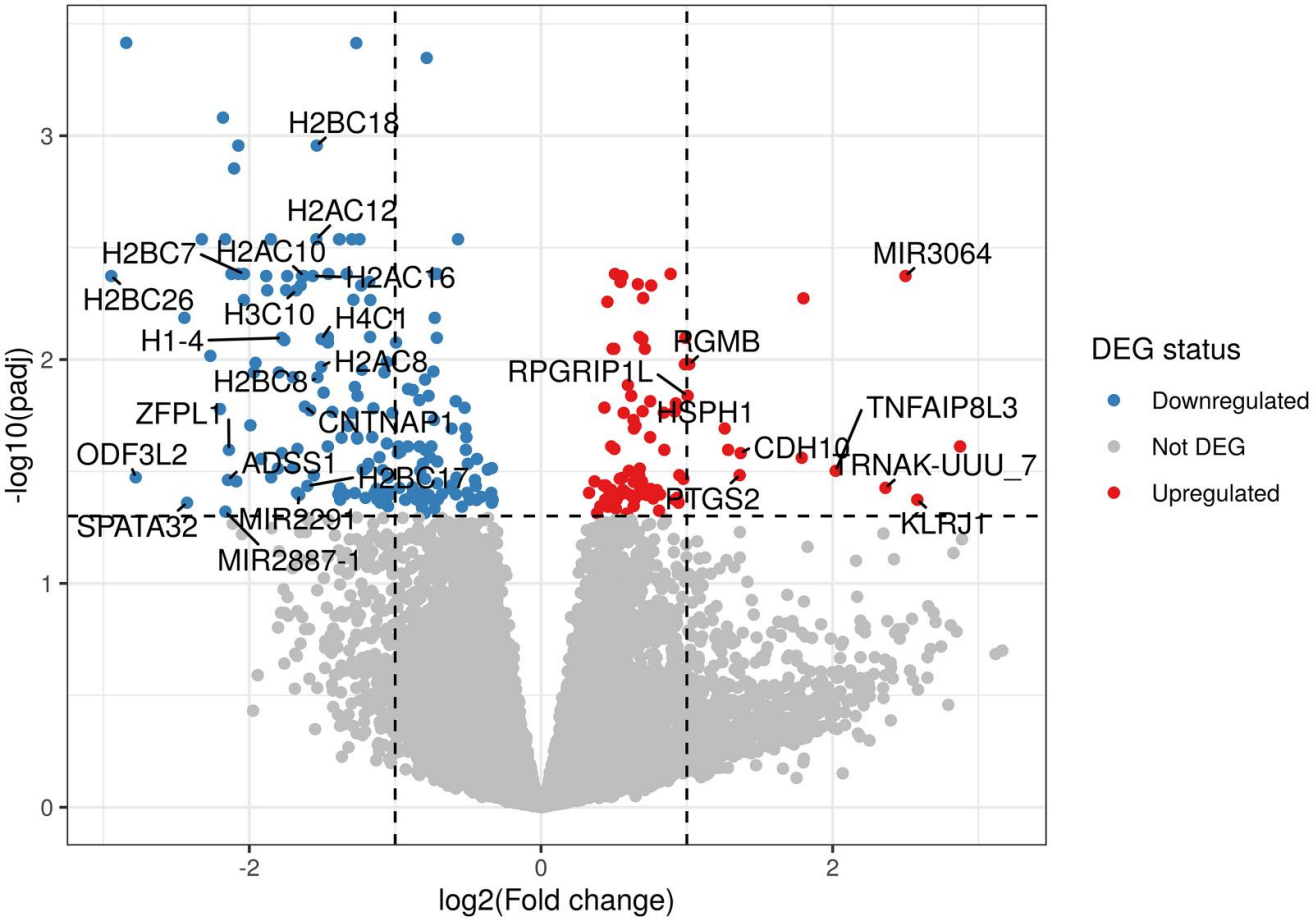
Volcano plot depicting differentially expressed genes (DEG) from white blood cells of dairy calves exposed to heat stress for 5 days.

**Figure 4:**
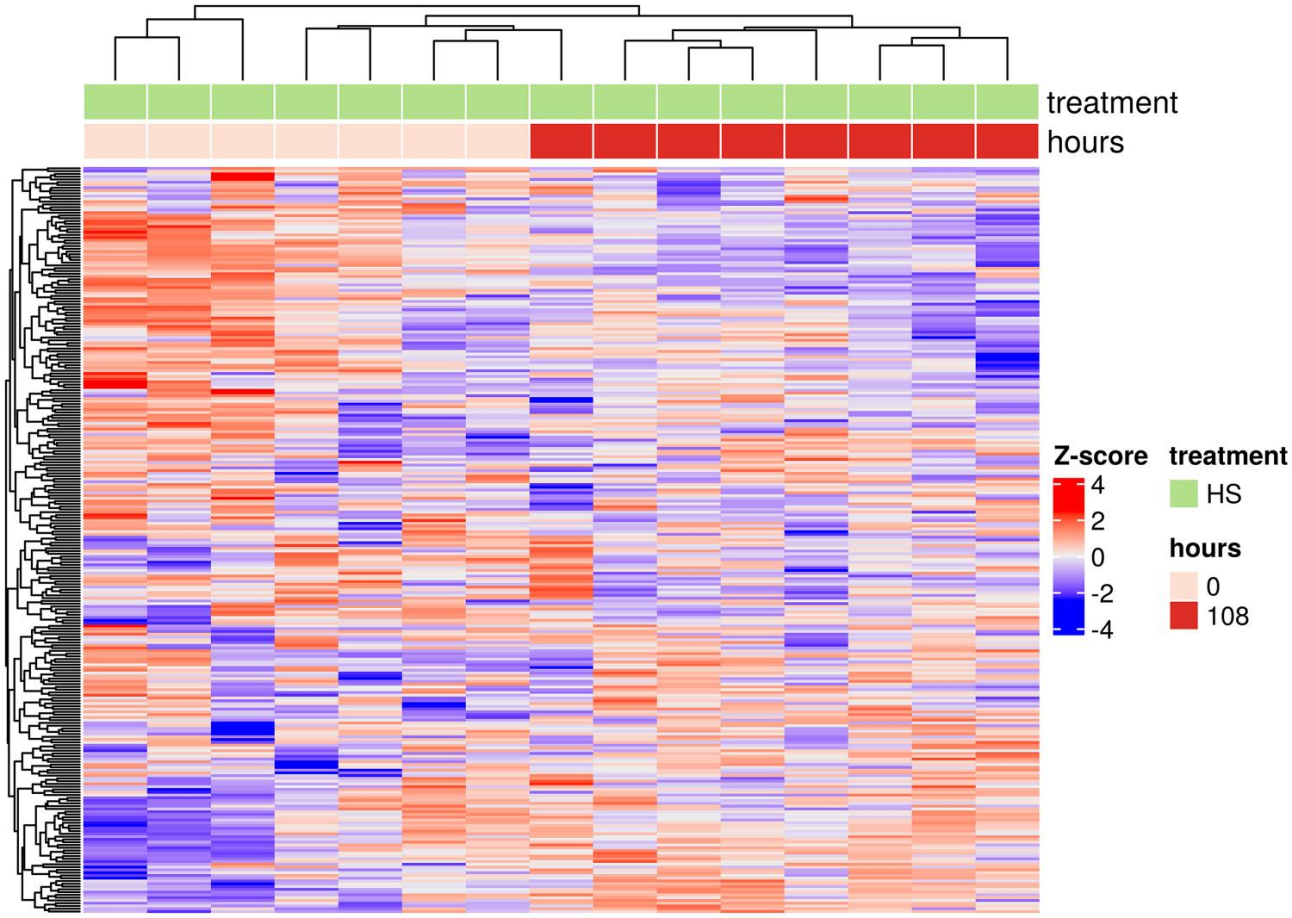
Heat map of differentially expressed genes from white blood cells of dairy calves exposed to heat stress on days 1 (0 hrs) and 5 (108 hours) of the treatment periods.

Compared with day 0, there were 1,133 genes in total impacted on day 5 of heat stress (i.e., 722 downregulated and 411 upregulated genes) out of 17,059 (Figures 3 and 4). A complete list of DEGs can be found in Additional Table S3. From 1,133 DEGs, 90 genes were uncharacterized, and their specific roles have not been elucidated (Additional Table S3). The effect of heat stress in the most impacted KEGG pathways were listed according to their number of DEGs following a descendent order. The KEGG pathway “Spliceosome” had 44 DEGs, mostly uridine-rich small nuclear RNA (i.e., U1, U2, U4 and U5 snRNA), downregulated due to heat stress (Additional Table S5). Components of the EJC/TREX spliceosome, Aly/REF export factor (*ALYREF*) and THO complex subunit 3 (*THOC3*) were downregulated and upregulated, respectively due to heat stress. The nuclear cap binding protein subunit 1 (*NCBP1*), a component of the spliceosome pathway, was upregulated by heat stress on day 5 compared with day 0 (Additional Table S3). Heat stress on day 5 upregulated the arginine and serine rich protein 1 (*RSRP1*) gene. Furthermore, compared with day 0, day 5 of heat stress upregulated the heat shock protein family A (Hsp70) member 8 (*HSPA8*) and downregulated the coiled-coil domain containing 12 (*CCDC12*, Additional Table S3).

Compared with day 0, day 5 of heat stress elicited a similar pattern in downregulation of DEGs associated with “Neutrophil extracellular trap formation,” “Systemic lupus erythematosus” and “Alcoholism” (Additional Tables S6–S8). Heat stress downregulated DEGs that codified for histone variants H2A (i.e., type 2-A or LOC788724, *H2AC4*, *H2AC7*, *H2AC8*, *H2AC10*, *H2AC11*, *H2AC12*, *H2AC13*, *H2AC15*, *H2AC16*, *H2AC17*, *H2AC21*, *H2AC25*, *H2AJ*), H2B (i.e., *H2BC3*, *H2BC6*, *H2BC7*, *H2BC8*, *H2BC9*, *H2BC11*, *H2BC12*, *H2BC17*, *H2BC18*, *H2BC20*, *H2BC26*), H3 (i.e., *H3C1*, *H3C2*, *H3C6*, *H3C10*, *H3C12*, *H3C13*) and H4 (i.e., *H4C1*, *H4C2*, *H4C8*, *H4C16*, Additional Tables S6–S8).

**Figure 7:**
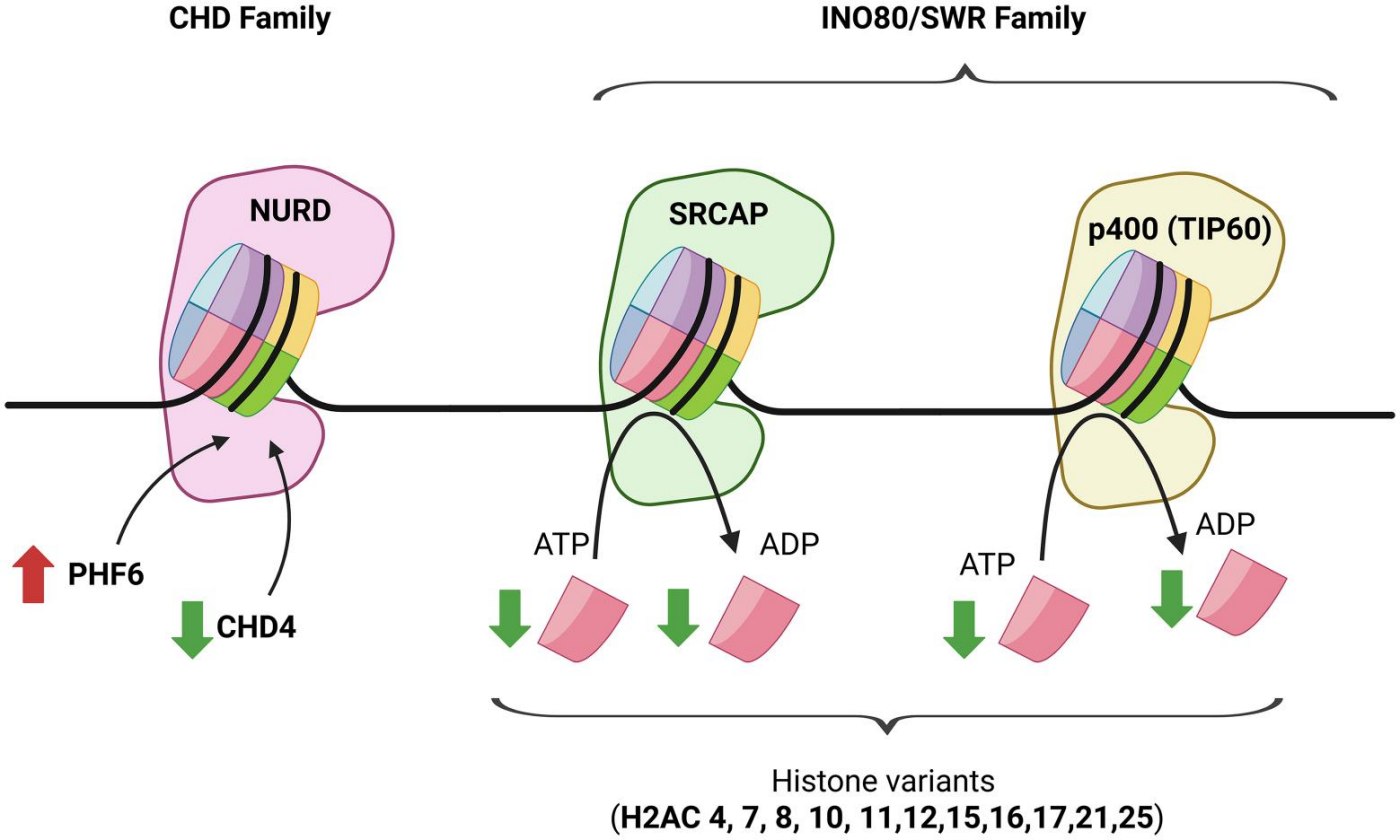
Heat stress effect on ATP-dependent chromatin remodeling. Plant homeo-domain finger protein 6 (PHF6); Chromodomain helicase DNA binding protein 4 (CHD4). Black arrows coming from histones represent histone variant exchange, one canonical histone is removed and replaced with a histone variant. Green arrows represent gene inhibition and red arrows represent gene activation. *Note.* Plant HomeoDomain finger protein 6 (PHD6); Chromodomain helicase DNA binding protein 4 (CHD4). Black arrows coming from histones represent histone variant exchange, one canonical histone is removed and replaced with a histone variant. Green arrows represent gene inhibition and red arrows represent gene activation. Created in BioRender. Moisá, S. (2025) https://BioRender.com/dj3uha7

The “ATP-dependent chromatin remodeling” KEGG pathway had 23 DEGs by heat stress on day 5 (Additional Tables S4 and S9). Compared with day 0, day 5 heat stress downregulated H2A clustered histone variants (i.e., type 2-A or *LOC788724*, *H2AC4*, *H2AC7*, *H2AC8*, *H2AC10*, *H2AC11*, *H2AC12*, *H2AC13*, *H2AC15*, *H2AC16*, *H2AC17*, *H2AC21*, *H2AC25, H2AJ*) which are components of the SRCAP and p400 (TIP60) complex (Fig. 7). Furthermore, two members of the CHD family—NURD complex were DEGs; chromodomain helicase DNA binding protein 4 (*CHD4*) was downregulated and PHD finger protein 6 (*PHD6*) was upregulated by heat stress treatment (Fig. 7).

When considering Gene Ontology (GO) terms describing biological functions, “heterochromatin formation,” was the only biological process in white blood cells altered by heat stress on day 5 compared with day 0 (Additional Table S4). Analysis of GO revealed heat stress impacted cellular functions located in the nucleus, cytoplasm, cytosol, nucleoplasm, nucleosome, chromatin and nuclear body. The “metal ion binding,” “DNA binding,” “identical protein binding,” “protein heterodimerization activity” and “structural constituent of chromatin” were the molecular functions impacted by heat stress in white blood cells. For more details about DEGs connected to these GO terms, refer to Additional Table S4.

The integrative protein signature database (INTERPRO) shows predictive models representing protein domains, families and functional sites. Compared with day 0, day 5 of heat stress elicited a prediction affecting “Histone-fold,” “Znf_C2H2_type,” “Histone_H2A/H2B/H3,” “SH2,” “Histone_H2A,” “Histone_H2A_C,” “Histone_H2A_CS,” “Histone_H2B_site,” “Histone_H3/CENP-A,” “CENP-T/H4_C,” “Histone_H4,” “TAF_TATA-bd_Histone-like_dom,” “Histone_H4_CS,” “Histone_H1/H5_H15” and “H1/H5” family proteins (Additional Table S4).

## Discussion

The objective of this study was to assess the transcriptome profile of white blood cells of dairy calves exposed to heat stress. Compared with day 0 (before heat stress started), days 1 to 5 of heat stress showed the physiological changes expected to see using our protocol for this study. In line with the objectives of this work, calves in the heat stress room were exposed to warm ambient temperatures for approximately 10 h per d and heat stress ranged from moderate (0900 to noon) to severe (noon to 1900). The changes in ambient temperature observed in the present study were similar to those observed in our previous studies on heat stress in dairy cattle (Kaufman et al. 2021; Ríus et al. 2022).

Compared with day 0, heat stress treatment on day 1 elicited a transcriptomic response in calves’ leukocytes. The heat stress response was mediated through the downregulation of *CIRBP* gene in leukocytes. The literature indicated that the cold-inducible RNA-binding protein (CIRBP) and the HSR pathways respond differently to changes in temperature, with CIRBP being primarily activated by hypothermia while HSR activated by heat stress (Gardela et al. 2019, Corre and Lebreton, 2024). In contrast to the HSR, heat stress downregulated *CIRBP* expression in most contexts (Zhu et al. 2016). In our study, heat stress treatment upregulated *STIP1* in white blood cells. The expression of *STIP1*, also known as HSP70/HSP90 organizing protein, activates cellular stress responses and facilitates protein folding, and it is part of the HSR pathway (Tsai et al. 2016, Singh et al. 2024). Collectively in our study, the inhibition of *CIRBP* expression and the upregulation of *STIP1* expression could be part of an early response to preserve leukocytes’ function in calves exposed to heat stress.

Compared with day 0, our results on day 1 showed that the heat stress treatment upregulated Nocturnin (*NOCT)* in white blood cells. Nocturnin is rhythmically expressed in tissues (eg liver and adipose) to regulate metabolism under the control of circadian clock in mice (Stubblefield et al. 2018). In addition to this circadian expression, Nocturnin is also acutely inducible by several stimuli (eg nutrient status), revealing immediate early gene responses (Onder et al. 2019). The endotoxin lipopolysaccharide (LPS), a well-known immunogenic stimulus, acutely induced Nocturnin expression in vitro. Furthermore, mice lacking Nocturnin improved survival following LPS injection exposing the link between the circadian rhythms and pro-inflammatory signals (Niu et al. 2011). The literature reported that leukocytes’ response is heavily influenced by the circadian rhythm and during the nocturnal resting phase, immune cells migrate from the peripheral blood to the bone marrow and lymph nodes, while the immune system’s pro-inflammatory activity increases. In our study, on day 1 of heat stress the expression of *NOCT* may be part of a mechanism to promptly coordinate lipid metabolism to support the activation of a pro-inflammatory response.

Compared with day 0, day 5 transcriptomic results revealed that heat stress upregulated the expression of *HSPA8* (Heat shock protein family A (HSP70), member 8). The HSP70 protein binds to hydrophobic regions of unfolded polypeptides and attracts auxiliary factors to accomplish ATP-dependent repair of proteins (Yost et al. 1990; Mayer and Bukau, 2005). As discussed above the effect of heat stress on HSR pathways on day 1, the pathway is upregulated to assist in protein folding, thus protecting cells from heat damage (Singh et al. 2024). In addition, heat stress upregulated the expression of *LOC786726* (Heterogeneous nuclear ribonucleoprotein k-like), a member of RNA-binding protein complex, in white blood cells. The expression of Heterogeneous nuclear ribonucleoprotein k-like protein regulates the activity of HSR pathway preventing the overexpression of HSP (Hong et al. 2001; Kim et al. 2017). Collectively, the analysis of transcriptomic results on day 5 showed that the HSR pathway remains active probably to maintain the protein functions in leukocytes of calves exposed to prolong heat stress.

**Figure 5:**
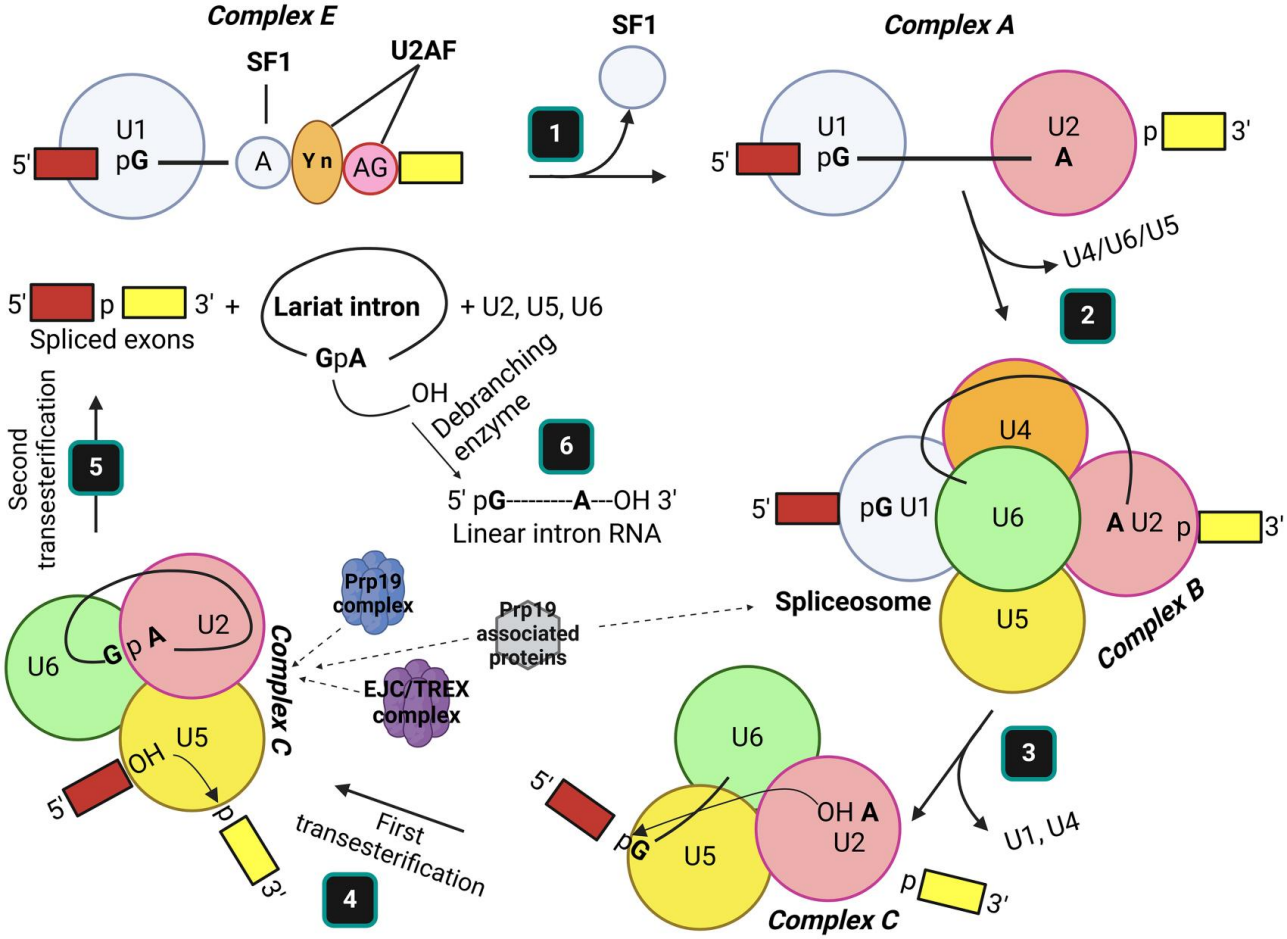
Spliceosome pathway steps: 1) after U1 base pair with the consensus 5’ splice site, SF1 (Splicing Factor 1) binds the branch point A; U2AF (U2 snRNP associated factor) associates with the polypyrimidine tract and 3’ splice site, and the U2 snRNP associates with the branch point A via base-paring interactions, displacing SF1. 2) A trimeric snRNP complex of U4/U5/U6 joins the initial complex to form the spliceosome. 3) Rearrangements of base-pairing interactions between snRNAs transform the spliceosome into a catalytically active unit and destabilize the U1 and U4 snRNPs, which are released. 4) The catalytic core (i.e., U6/U2), then catalyzes the first transesterification reaction. 5) Following further reorganizations between the snRNPs, the second transesterification reaction connects to the 2 exons and releases the intron as a lariat structure together with the remaining snRNPs. 6) The removed lariat intron is transformed into a linear RNA by a debranching enzyme. In this spliceosome pathway figure, the effect of heat stress in white blood cells of dairy calves is represented through the EJC/TREX complex, the Prp19 complex and associated proteins. Modified from Lodish et al. 2013 (Lodish et al. 2013).

The analysis of transcriptomic results on day 5 revealed that the heat stress treatment downregulated several uridine-rich small nuclear RNA of the “spliceosome” (i.e., U1, U2, U4 and U5 snRNA) in leukocytes, according to the KEGG pathway (Fig. 5 and Additional Table S5). To distinguish ribonucleoproteins (RNP) subunits from other cellular RNPs, the spliceosomal subunits are termed small nuclear RNPs (snRNPs, (Jurica and Moore 2003). Each snRNP presents a uridine-rich snRNA, ie U1, U2, U4, U5 or U6, and a specific set of interacting proteins. Bond (1988) showed that at the post-translational level, splicing of pre-mRNA could be inhibited by heat stress. In addition in previous studies, hyperthermia produced inactivation of U1 and U2, and degradation of the triple complex snRNPs U4/U5/U6 in HeLa cells (Shukla et al. 1990; Utans et al. 1992). In our transcriptome results of day 0 against day 5, showed that the response to heat stress was post-transcriptionally regulated by an inhibition of most of the snRNP components of the splicing machinery. Therefore, our results suggested that leukocytes’ transcriptome responded to heat stress treatment by inhibiting the maturation of mRNA. It is possible that this post-translational inhibition of the spliceosome machinery was performed to prevent the synthesis of abnormal proteins (Yost and Lindquist 1988).

The analysis of transcriptomic results on day 5 against day 0 revealed that the heat stress treatment downregulated the expression of *CCDC12* (coiled-coil domain containing 12) in leukocytes. The Prp19 complex is key during the splicing reaction because it is responsible for the assembly of the tri-snRNP complex U2/U5/U6 (Chan et al. 2003) and remains associated with the spliceosome during step 3 and 4 of splicing (Fig. 5). The *CCDC12* factor has an important role in the spliceosome assembly by intervening in its tetramerization (Grote et al. 2010). Coiled-coil domains play an essential role in detecting changes in ambient temperature (Upadhyay et al. 2021) and in the assembly and function of the spliceosome (Ford and Fioriti 2020). In our study heat stress downregulated *CCDC12* expression in white blood cells. We speculate that the downregulation of *CCDC12* expression could be part of a coordinated mechanism to reduce synthesis of proteins.

The analysis of transcriptomic results of day 0 versus day 5 revealed that the heat stress treatment downregulated the “Neutrophil extracellular trap formation,” “Systemic lupus erythematosus,” and “Alcoholism” KEGG pathways mainly through downregulating the expression of histone variants genes (Additional Tables S6–S8). Neutrophils effectively remove harmful molecules through phagocytosis, degranulation, and the release of neutrophil extracellular traps (NETs) pathways. The NETs pathway can be triggered by exogenous and endogenous stimuli (e.g., LPS and abnormal proteins), and immune complexes. Upon activation of the pathway, NETs form large, extracellular, web-like sticky meshes. For example, NETs developed from posttranslational modification of positively charged residues in core (i.e., H2A, H2B, H3, H4) and linker histone H1 and the H2A-H2B-DNA complex (Neeli et al. 2008). Studies in mice showed that an acute (60 min) bout of hyperthermia increased NETs formation, activated the coagulation system, and led to disseminated intravascular coagulation (Zhang et al. 2022). Our previous work in dairy calf and cows showed that heat stress increased intestinal permeability to lumen antigens (Ríus et al. 2022; Yu et al. 2024) and blood concentration of markers of inflammation (eg haptoglobin; Kaufman et al. 2021). In the present study, it is possible that the downregulation of NETs pathway in neutrophils was part of a coordinated response to tightly control the pro-inflammatory response elicited on day 5 of the study.

Previous work in rodents showed that a dysregulated NETs pathway contributed to the pathogenesis of immune-related diseases (e.g., lupus erythematosus, Bosch 2011; Lee et al. 2017). Recently it was reported that neutrophils, through NETs formation and altered degradation, transitioned from defenders innate immune cells to drivers of autoimmune attack in systemic lupus erythematosus (Wigerblad and Kaplan 2023). Indeed, high levels of NETs (or their components like cell-free DNA) in the blood were associated with active lupus (Xu et al. 2018) and autoimmune outbreaks were developed by inducing inflammation (Hedrich and Tsokos 2011). Systemic lupus erythematosus is uncommon in cattle. The downregulation of the lupus pathway in our study may reveal a key component of immune-related pathways tightly regulated to control the inflammatory response. Therefore, in our study it is possible to speculate that compared with day 0 on day 5 of heat stress the downregulation of the lupus pathways was associated with a leukocyte response to lower the inflammatory response.

Regarding the change of the alcoholism pathway in our study, in comparison, reports of alcohol consumption in primates showed a dysregulation of leukocytes towards a hyper-inflammatory state which made the immune system overreact to antigens while simultaneously impaired the immune function (Lewis et al. 2023). Malherbe and Messaoudi (2022) concluded that the activation of the alcoholism pathway led to increased expression of inflammatory genes and reduced expression of genes for fighting microbes, creating “poised” cells ready to overreact. In comparison, in our study the downregulation of the alcoholism pathway due to heat stress may reveal a key component of immune-related pathways tightly regulated to avoid dysregulation of leukocytes and the phenotype of chronic inflammation. Therefore, in our study it is possible to speculate that on day 5 of heat stress the downregulation of the alcoholism pathway was associated with a leukocyte response to lower the inflammatory response.

The analysis of transcriptomic results of day 0 versus day 5 showed an upregulation of *CR1L* due to heat stress in white blood cells of dairy calves (Additional Table S6). In rodents, C3b and C4b are crucial proteins factors of the complement system that coat antigens, marking them for destruction through enhanced attachment (ie phagocytosis, Brown et al. 2020). The complement C3b/C4b receptor 1 protein (CR1L) is a membrane-bound receptor that binds to C3b/C4b fragments on antigens or immune complexes to help clear them in the liver or spleen, modulate inflammation, and prevent autoimmune issues (Brown et al. 2020; Chaudhary et al. 2022). In comparison in our study using dairy calves, the upregulation of *CR1L* due to heat stress may reveal a key component of immune-related pathways tightly regulated to prevent autoimmune damage while still effectively removing antigens (Cantet et al. 2021). Together, in our study it is possible to speculate that heat stress upregulation of *CR1L* was associated with an immune response to remove antigens from circulation.

Day 5 against day 0 transcriptome analysis suggested that heat stress downregulated chromatin remodeling pathways in leukocytes. The ATP-dependent chromatin remodeling KEGG pathway showed several downregulated H2A clustered histone variants (Fig. 7 and Additional Table S9). Chromatin experienced modifications that can produce alterations in gene expression but without changing the genetic code present in the DNA (i.e., epigenetic changes, (Bhadouriya et al. 2020; Nodelman and Bowman 2021). Our transcriptomics results suggest that heat stress could inhibit chromatin remodeling and several histone variants. It is possible that on day 5 heat stress repressed the expression of proteins such as pro-inflammatory cytokines in leukocytes.

**Figure 6:**
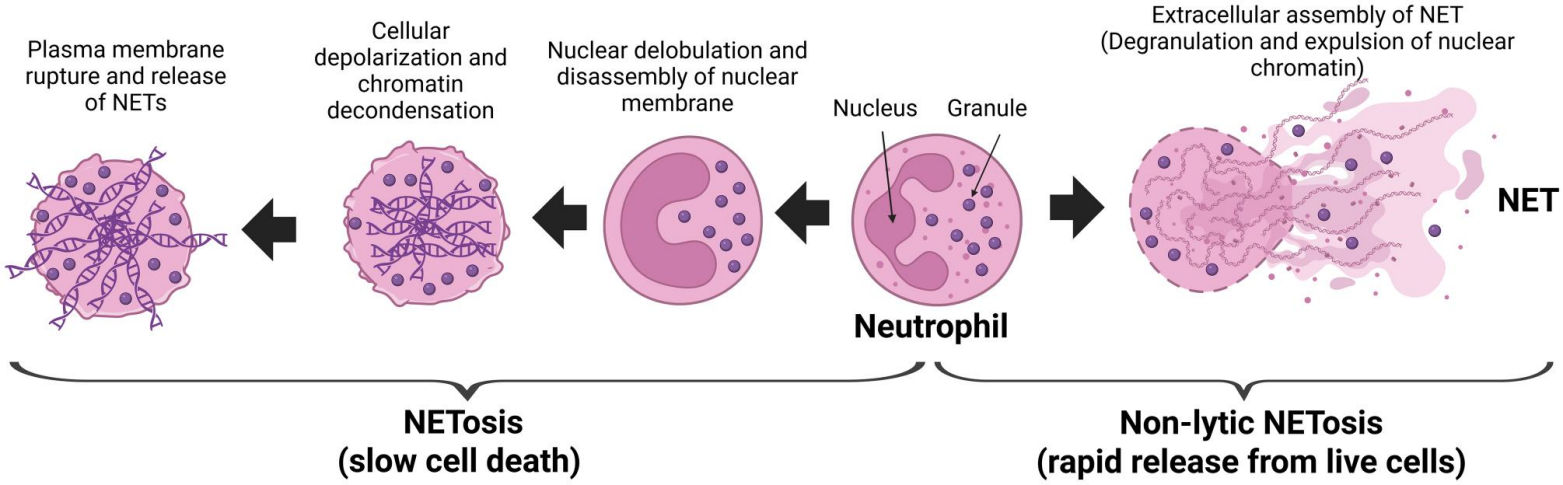
Neutrophil extracellular trap formation pathway. Neutrophil extracellular trap forms in two different ways: by rapid release from live cells (Non-lytic NETosis) or by a slow cell death (NETosis). Our transcriptomics data does not allow us to determine what pathway is predominant. There is only evidence that heat stress has an impact on this metabolic pathway. Modified from Papayannopoulos, 2018 (Papayannopoulos, 2018). Created in BioRender. Moisá, S. (2025) https://BioRender.com/k1c9eun

## Conclusions

Transcriptional changes on day 1 included the upregulation of pathways associated with the preservation of protein functions and changes in lipid metabolism to support the activation of immune cells. These changes in leukocytes transcriptome may anticipate the increasing plasma concentrations of biomarkers of inflammation typically observed immediately during heat stress. The analysis of transcriptome on day 5 revealed that the heat stress treatment downregulated pathways associated with NETs formation, systemic lupus erythematosus, and alcoholism possible to reduce a pro-inflammatory response and prevent dysregulation of leukocytes’ functions. These changes in leukocytes transcriptome may correlate with observations of intestinal inflammation during heat stress. Our results support the concept that the study of transcriptome has the potential to increase the understanding of heat stress effect on the immune function and metabolism of growing dairy calves. These findings may reveal biological pathways of key physiological, immunological and metabolic functions and the potential use of natural or synthetic modulators to ameliorate the effect of heat stress (eg immunomodulators or anti-inflammatory feed additives).

## Supplementary Material

txag029_Supplementary_Data
