# Supplementary material for: Transcriptome changes in leukocytes of dairy calves exposed to heat stress
Source: Transl Anim Sci. 2026 Mar 15;10:txag029. doi: 10.1093/tas/txag029 (PMC13152581; doi:10.1093/tas/txag029)
Supplement: txag029_Supplementary_Data [file txag029_supplementary_data.zip › Additional Table 1.docx]

**Additional Table 1**

| **feature** | **ncbi_gene_id** | **description** | **logFC** | **P.Value** | **adj.P.Val** | **contrast_name** |
| --- | --- | --- | --- | --- | --- | --- |
| LOC100847791 | 100847791 | None | -1.3 | 0.000 | 0.004 | HS_1_vs_HS_0 |
| CIRBP | 507120 | None | -0.7 | 0.000 | 0.044 | HS_1_vs_HS_0 |
| C17H5orf52 | 787653 | chromosome 17 C5orf52 homolog | -1.2 | 0.000 | 0.044 | HS_1_vs_HS_0 |
| STIP1 | 617109 | stress induced phosphoprotein 1 | 0.9 | 0.000 | 0.044 | HS_1_vs_HS_0 |
| NOCT | 540641 | nocturnin | 0.7 | 0.000 | 0.050 | HS_1_vs_HS_0 |
| LOC101907327 | 101907327 | None | -3.0 | 0.000 | 0.000 | HS_5_vs_HS_0 |
| CIRBP | 507120 | None | -0.8 | 0.000 | 0.001 | HS_5_vs_HS_0 |
| C17H5orf52 | 787653 | chromosome 17 C5orf52 homolog | -1.3 | 0.000 | 0.001 | HS_5_vs_HS_0 |
| H2BC6 | 787465 | H2B clustered histone 19 | -1.3 | 0.000 | 0.001 | HS_5_vs_HS_0 |
| H1-2 | 513971 | H1.2 linker histone, cluster member | -1.2 | 0.000 | 0.001 | HS_5_vs_HS_0 |
| H2BC18 | 615091 | None | -1.6 | 0.000 | 0.001 | HS_5_vs_HS_0 |
| H2AC12 | 616634 | None | -1.6 | 0.000 | 0.001 | HS_5_vs_HS_0 |
| LOC112444678 | 112444678 | Small nucleolar RNA U13 | -2.2 | 0.000 | 0.001 | HS_5_vs_HS_0 |
| RBM3 | 509771 | RNA binding motif protein 3 | -0.8 | 0.000 | 0.001 | HS_5_vs_HS_0 |
| LOC100847791 | 100847791 | None | -1.0 | 0.000 | 0.001 | HS_5_vs_HS_0 |
| LOC112443159 | 112443159 | None | -2.0 | 0.000 | 0.001 | HS_5_vs_HS_0 |
| PAQR6 | 516498 | progestin and adipoQ receptor family member 6 | -1.3 | 0.000 | 0.002 | HS_5_vs_HS_0 |
| ASIC1 | 538244 | acid sensing ion channel subunit 1 | -1.4 | 0.000 | 0.002 | HS_5_vs_HS_0 |
| ZNF526 | 514418 | zinc finger protein 526 | -1.0 | 0.000 | 0.002 | HS_5_vs_HS_0 |
| LOC100850875 | 100850875 | None | -1.6 | 0.000 | 0.002 | HS_5_vs_HS_0 |
| LOC112448638 | 112448638 | U5 spliceosomal RNA | -2.1 | 0.000 | 0.002 | HS_5_vs_HS_0 |
| IQCN | 788637 | None | -0.7 | 0.000 | 0.003 | HS_5_vs_HS_0 |
| ANGEL1 | 508197 | None | -0.8 | 0.000 | 0.003 | HS_5_vs_HS_0 |
| STK40 | 515723 | serine/threonine kinase 40 | -0.6 | 0.000 | 0.003 | HS_5_vs_HS_0 |
| JUND | 517192 | None | -1.5 | 0.000 | 0.003 | HS_5_vs_HS_0 |
| MAP1A | 515593 | microtubule associated protein 1A | -1.3 | 0.000 | 0.003 | HS_5_vs_HS_0 |
| IGHMBP2 | 618606 | immunoglobulin mu DNA binding protein 2 | -0.7 | 0.000 | 0.003 | HS_5_vs_HS_0 |
| LOC781785 | 781785 | None | -1.1 | 0.000 | 0.003 | HS_5_vs_HS_0 |
| LOC107132278 | 107132278 | U1 spliceosomal RNA | -2.0 | 0.000 | 0.003 | HS_5_vs_HS_0 |
| LOC112446424 | 112446424 | None | 1.4 | 0.000 | 0.003 | HS_5_vs_HS_0 |
| LOC101907887 | 101907887 | None | -1.4 | 0.000 | 0.003 | HS_5_vs_HS_0 |
| AMDHD2 | 521401 | amidohydrolase domain containing 2 | -0.6 | 0.000 | 0.003 | HS_5_vs_HS_0 |
| ABRAXAS1 | 504796 | abraxas 1, BRCA1 A complex subunit | 0.7 | 0.000 | 0.003 | HS_5_vs_HS_0 |
| HSPH1 | 507165 | heat shock protein family H (Hsp110) member 1 | 1.3 | 0.000 | 0.003 | HS_5_vs_HS_0 |
| LOC104976009 | 104976009 | None | 0.9 | 0.000 | 0.003 | HS_5_vs_HS_0 |
| H2BC17 | 616776 | H2B clustered histone 19 | -1.5 | 0.000 | 0.004 | HS_5_vs_HS_0 |
| C10H14orf93 | 506904 | chromosome 10 C14orf93 homolog | -0.5 | 0.000 | 0.004 | HS_5_vs_HS_0 |
| CTDSP1 | 516199 | None | -0.7 | 0.000 | 0.004 | HS_5_vs_HS_0 |
| MIDN | 523615 | midnolin | -1.0 | 0.000 | 0.004 | HS_5_vs_HS_0 |
| LOC112448640 | 112448640 | U5 spliceosomal RNA | -2.4 | 0.000 | 0.004 | HS_5_vs_HS_0 |
| PGGHG | 511919 | None | -0.9 | 0.000 | 0.004 | HS_5_vs_HS_0 |
| CCER2 | 614600 | coiled-coil glutamate rich protein 2 | -1.1 | 0.000 | 0.004 | HS_5_vs_HS_0 |
| LOC132343001 | 132343001 | None | -1.8 | 0.000 | 0.004 | HS_5_vs_HS_0 |
| LOC112442854 | 112442854 | U2 spliceosomal RNA | -2.3 | 0.000 | 0.004 | HS_5_vs_HS_0 |
| H2BC7 | 505183 | H2B clustered histone 19 | -2.1 | 0.000 | 0.004 | HS_5_vs_HS_0 |
| LOC112448940 | 112448940 | Small nucleolar RNA U13 | -2.2 | 0.000 | 0.004 | HS_5_vs_HS_0 |
| FAM131B | 617268 | family with sequence similarity 131 member B | -1.5 | 0.000 | 0.004 | HS_5_vs_HS_0 |
| LOC132345501 | 132345501 | None | -1.0 | 0.000 | 0.004 | HS_5_vs_HS_0 |
| LOC112442807 | 112442807 | U1 spliceosomal RNA | -1.9 | 0.000 | 0.004 | HS_5_vs_HS_0 |
| H2AC17 | 104968446 | H2A clustered histone 14 | -1.5 | 0.000 | 0.004 | HS_5_vs_HS_0 |
| H2AC7 | 104975683 | H2A clustered histone 14 | -1.4 | 0.000 | 0.004 | HS_5_vs_HS_0 |
| PHYKPL | 537241 | 5-phosphohydroxy-L-lysine phospho-lyase | -0.6 | 0.000 | 0.005 | HS_5_vs_HS_0 |
| LOC132346654 | 132346654 | None | -1.2 | 0.000 | 0.005 | HS_5_vs_HS_0 |
| LOC112449629 | 112449629 | U1 spliceosomal RNA | -2.3 | 0.000 | 0.005 | HS_5_vs_HS_0 |
| H2AC11 | 616611 | H2A clustered histone 14 | -1.5 | 0.000 | 0.005 | HS_5_vs_HS_0 |
| TMEM134 | 510129 | transmembrane protein 134 | -0.6 | 0.000 | 0.005 | HS_5_vs_HS_0 |
| IFT57 | 531436 | intraflagellar transport 57 | 0.6 | 0.000 | 0.005 | HS_5_vs_HS_0 |
| CYP2R1 | 541302 | cytochrome P450 family 2 subfamily R member 1 | 0.7 | 0.000 | 0.005 | HS_5_vs_HS_0 |
| RPGRIP1L | 518659 | RPGRIP1 like | 1.0 | 0.000 | 0.005 | HS_5_vs_HS_0 |
| H2BC12 | 616627 | None | -1.5 | 0.000 | 0.005 | HS_5_vs_HS_0 |
| MIR2291 | 100313118 | bta-mir-2291 | -1.6 | 0.000 | 0.005 | HS_5_vs_HS_0 |
| PCIF1 | 535479 | phosphorylated CTD interacting factor 1 | -0.5 | 0.000 | 0.005 | HS_5_vs_HS_0 |
| LOC112442700 | 112442700 | None | -0.9 | 0.000 | 0.005 | HS_5_vs_HS_0 |
| UFSP2 | 617788 | UFM1 specific peptidase 2 | 0.5 | 0.000 | 0.005 | HS_5_vs_HS_0 |
| C15H11orf71 | 615529 | chromosome 15 C11orf71 homolog | -0.7 | 0.000 | 0.005 | HS_5_vs_HS_0 |
| GATA3 | 505169 | GATA binding protein 3 | -1.0 | 0.000 | 0.005 | HS_5_vs_HS_0 |
| SLC16A6 | 529171 | solute carrier family 16 member 6 | -0.8 | 0.000 | 0.005 | HS_5_vs_HS_0 |
| H1-10 | 511901 | None | -0.8 | 0.000 | 0.005 | HS_5_vs_HS_0 |
| NOTCH1 | 767866 | notch receptor 1 | -0.7 | 0.000 | 0.005 | HS_5_vs_HS_0 |
| LOC112444557 | 112444557 | small Cajal body-specific RNA 18 | -1.1 | 0.000 | 0.005 | HS_5_vs_HS_0 |
| LOC112442425 | 112442425 | small nucleolar RNA, C/D box 35A | -0.8 | 0.000 | 0.006 | HS_5_vs_HS_0 |
| H2BC3 | 525512 | H2B clustered histone 3 | -1.2 | 0.000 | 0.006 | HS_5_vs_HS_0 |
| RPS6KA2 | 517953 | ribosomal protein S6 kinase A2 | -0.9 | 0.000 | 0.006 | HS_5_vs_HS_0 |
| BAHCC1 | 786025 | None | -0.8 | 0.000 | 0.006 | HS_5_vs_HS_0 |
| H1-12 | 515957 | H1.12 linker histone, cluster member | -1.2 | 0.000 | 0.006 | HS_5_vs_HS_0 |
| LOC112449404 | 112449404 | small nucleolar RNA, C/D box 12C | -1.2 | 0.000 | 0.006 | HS_5_vs_HS_0 |
| LOC112446153 | 112446153 | U5 spliceosomal RNA | -1.9 | 0.000 | 0.006 | HS_5_vs_HS_0 |
| ZNF512B | 614014 | None | -1.2 | 0.000 | 0.006 | HS_5_vs_HS_0 |
| H4C1 | 617905 | H4 histone 16 | -1.6 | 0.000 | 0.006 | HS_5_vs_HS_0 |
| VSIR | 783068 | V-set immunoregulatory receptor | -0.8 | 0.000 | 0.006 | HS_5_vs_HS_0 |
| LOC132344334 | 132344334 | None | -1.2 | 0.000 | 0.006 | HS_5_vs_HS_0 |
| DYNLT4 | 516323 | dynein light chain Tctex-type 4 | -0.9 | 0.000 | 0.006 | HS_5_vs_HS_0 |
| FAM170B | 615859 | family with sequence similarity 170 member B | -1.0 | 0.000 | 0.007 | HS_5_vs_HS_0 |
| IFITM2 | 615833 | None | -0.8 | 0.000 | 0.007 | HS_5_vs_HS_0 |
| LCN2 | 526639 | None | -0.6 | 0.000 | 0.007 | HS_5_vs_HS_0 |
| DNAJB13 | 520270 | DnaJ heat shock protein family (Hsp40) member B13 | -1.5 | 0.000 | 0.007 | HS_5_vs_HS_0 |
| GPR141 | 538837 | G protein-coupled receptor 141 | 1.0 | 0.000 | 0.007 | HS_5_vs_HS_0 |
| H1-5 | 527304 | H1.5 linker histone, cluster member | -1.4 | 0.000 | 0.007 | HS_5_vs_HS_0 |
| TLE3 | 514326 | TLE family member 3, transcriptional corepressor | -0.9 | 0.000 | 0.007 | HS_5_vs_HS_0 |
| H2BC26 | 520120 | None | -3.0 | 0.000 | 0.007 | HS_5_vs_HS_0 |
| H3C10 | 616819 | H3 clustered histone 6 | -1.8 | 0.000 | 0.007 | HS_5_vs_HS_0 |
| CABIN1 | 530023 | calcineurin binding protein 1 | -1.0 | 0.000 | 0.008 | HS_5_vs_HS_0 |
| NUPR2 | 614047 | None | -0.7 | 0.000 | 0.008 | HS_5_vs_HS_0 |
| LOC112443327 | 112443327 | U1 spliceosomal RNA | -2.3 | 0.000 | 0.008 | HS_5_vs_HS_0 |
| NQO2 | 508566 | N-ribosyldihydronicotinamide:quinone dehydrogenase 2 | -0.7 | 0.000 | 0.008 | HS_5_vs_HS_0 |
| LOC782951 | 782951 | None | 1.2 | 0.000 | 0.008 | HS_5_vs_HS_0 |
| CDK10 | 615171 | None | -0.5 | 0.000 | 0.008 | HS_5_vs_HS_0 |
| RHOG2 | 531038 | None | 0.5 | 0.000 | 0.008 | HS_5_vs_HS_0 |
| MIS12 | 767858 | MIS12 kinetochore complex component | 1.0 | 0.000 | 0.008 | HS_5_vs_HS_0 |
| LOC112443671 | 112443671 | None | -1.6 | 0.000 | 0.008 | HS_5_vs_HS_0 |
| LOC112442111 | 112442111 | U4 spliceosomal RNA | -1.8 | 0.000 | 0.008 | HS_5_vs_HS_0 |
| LOC112443342 | 112443342 | U1 spliceosomal RNA | -2.0 | 0.000 | 0.008 | HS_5_vs_HS_0 |
| BAIAP3 | 132342081 | BAI1 associated protein 3 | -1.4 | 0.000 | 0.008 | HS_5_vs_HS_0 |
| LOC112446726 | 112446726 | None | 0.8 | 0.000 | 0.008 | HS_5_vs_HS_0 |
| FSD1L | 510535 | fibronectin type III and SPRY domain containing 1 like | 0.7 | 0.000 | 0.008 | HS_5_vs_HS_0 |
| MIR3064 | 104796064 | bta-mir-3064 | 2.4 | 0.000 | 0.008 | HS_5_vs_HS_0 |
| SIRT3 | 614027 | sirtuin 3 | -0.5 | 0.000 | 0.009 | HS_5_vs_HS_0 |
| SEPTIN9 | 100140583 | septin 9 | -0.6 | 0.000 | 0.009 | HS_5_vs_HS_0 |
| MARS2 | 514726 | methionyl-tRNA synthetase 2, mitochondrial | 0.7 | 0.000 | 0.009 | HS_5_vs_HS_0 |
| LOC112449452 | 112449452 | Small nucleolar RNA SNORA70 | 1.6 | 0.000 | 0.009 | HS_5_vs_HS_0 |
| TASL | 513911 | None | 1.0 | 0.000 | 0.009 | HS_5_vs_HS_0 |
| LOC112442845 | 112442845 | U2 spliceosomal RNA | -2.2 | 0.000 | 0.009 | HS_5_vs_HS_0 |
| SPRY1 | 507095 | sprouty RTK signaling antagonist 1 | 1.3 | 0.000 | 0.009 | HS_5_vs_HS_0 |
| IFT43 | 513228 | intraflagellar transport 43 | -0.8 | 0.000 | 0.010 | HS_5_vs_HS_0 |
| NUDT16L1 | 518085 | None | -0.8 | 0.000 | 0.010 | HS_5_vs_HS_0 |
| CCDC85B | 540332 | coiled-coil domain containing 85B | -1.5 | 0.000 | 0.010 | HS_5_vs_HS_0 |
| LOC101909736 | 101909736 | None | 0.7 | 0.000 | 0.010 | HS_5_vs_HS_0 |
| HSPA8 | 281831 | None | 0.9 | 0.000 | 0.010 | HS_5_vs_HS_0 |
| H2AC21 | 614974 | H2A clustered histone 18 | -0.9 | 0.000 | 0.010 | HS_5_vs_HS_0 |
| JAZF1 | 616701 | JAZF zinc finger 1 | -1.0 | 0.000 | 0.010 | HS_5_vs_HS_0 |
| H1-0 | 617975 | H1.0 linker histone | -1.3 | 0.000 | 0.010 | HS_5_vs_HS_0 |
| CERK | 100336737 | None | -0.7 | 0.000 | 0.010 | HS_5_vs_HS_0 |
| HNRNPD | 527471 | heteroous nuclear ribonucleoprotein D | -0.5 | 0.000 | 0.010 | HS_5_vs_HS_0 |
| EEIG1 | 615907 | None | -1.0 | 0.000 | 0.010 | HS_5_vs_HS_0 |
| SDF4 | 528783 | None | -0.8 | 0.000 | 0.010 | HS_5_vs_HS_0 |
| LOC112442857 | 112442857 | U2 spliceosomal RNA | -2.1 | 0.000 | 0.010 | HS_5_vs_HS_0 |
| H2BC8 | 787581 | None | -1.6 | 0.000 | 0.010 | HS_5_vs_HS_0 |
| SOX4 | 768313 | SRY-box transcription factor 4 | -1.2 | 0.000 | 0.010 | HS_5_vs_HS_0 |
| PRSS33 | 528784 | None | -0.5 | 0.000 | 0.010 | HS_5_vs_HS_0 |
| CLEC16A | 615054 | C-type lectin domain containing 16A | -0.6 | 0.000 | 0.010 | HS_5_vs_HS_0 |
| TSPAN6 | 514741 | tetraspanin 6 | 0.6 | 0.000 | 0.010 | HS_5_vs_HS_0 |
| XKRX | 524975 | None | 1.0 | 0.000 | 0.010 | HS_5_vs_HS_0 |
| ANKMY2 | 509032 | ankyrin repeat and MYND domain containing 2 | 0.6 | 0.000 | 0.010 | HS_5_vs_HS_0 |
| ATN1 | 513125 | atrophin 1 | -1.1 | 0.000 | 0.010 | HS_5_vs_HS_0 |
| TSNARE1 | 535306 | t-SNARE domain containing 1 | -0.6 | 0.000 | 0.010 | HS_5_vs_HS_0 |
| GALNS | 515809 | galactosamine (N-acetyl)-6-sulfatase | -0.5 | 0.000 | 0.010 | HS_5_vs_HS_0 |
| UQCC1 | 618503 | ubiquinol-cytochrome c reductase complex assembly factor 1 | 0.5 | 0.000 | 0.010 | HS_5_vs_HS_0 |
| RSRP1 | 615263 | None | 1.0 | 0.000 | 0.010 | HS_5_vs_HS_0 |
| CIC | 538483 | capicua transcriptional repressor | -1.2 | 0.000 | 0.010 | HS_5_vs_HS_0 |
| CSK | 509246 | C-terminal Src kinase | -0.7 | 0.000 | 0.010 | HS_5_vs_HS_0 |
| LOC521580 | 521580 | None | -0.9 | 0.000 | 0.010 | HS_5_vs_HS_0 |
| NPRL3 | 534485 | None | -0.6 | 0.000 | 0.010 | HS_5_vs_HS_0 |
| TKFC | 512373 | triokinase and FMN cyclase | -0.5 | 0.000 | 0.010 | HS_5_vs_HS_0 |
| LOC100299277 | 100299277 | None | -0.9 | 0.000 | 0.010 | HS_5_vs_HS_0 |
| YJU2B | 614791 | YJU2 splicing factor homolog B | -0.5 | 0.000 | 0.010 | HS_5_vs_HS_0 |
| LOC132345979 | 132345979 | None | -1.3 | 0.000 | 0.010 | HS_5_vs_HS_0 |
| BVD1.23 | 619142 | None | -1.5 | 0.000 | 0.010 | HS_5_vs_HS_0 |
| SPINT2 | 507484 | serine peptidase inhibitor, Kunitz type 2 | -0.9 | 0.000 | 0.010 | HS_5_vs_HS_0 |
| LOC112442840 | 112442840 | small nucleolar RNA, C/D box 10 | -1.8 | 0.000 | 0.010 | HS_5_vs_HS_0 |
| H2AC15 | 614970 | None | -1.2 | 0.000 | 0.010 | HS_5_vs_HS_0 |
| H2AC10 | 618824 | H2A clustered histone 14 | -1.7 | 0.000 | 0.010 | HS_5_vs_HS_0 |
| H1-4 | 617854 | H1.4 linker histone, cluster member | -1.8 | 0.000 | 0.010 | HS_5_vs_HS_0 |
| MAGED1 | 512562 | MAGE family member D1 | -0.5 | 0.000 | 0.010 | HS_5_vs_HS_0 |
| METTL3 | 540339 | methyltransferase 3, N6-adenosine-methyltransferase complex catalytic subunit | 0.6 | 0.000 | 0.010 | HS_5_vs_HS_0 |
| NDUFAF1 | 541232 | NADH:ubiquinone oxidoreductase complex assembly factor 1 | 0.5 | 0.000 | 0.010 | HS_5_vs_HS_0 |
| MED6 | 505293 | None | 0.5 | 0.000 | 0.010 | HS_5_vs_HS_0 |
| ZNF829 | 790881 | zinc finger protein 829 | 0.7 | 0.000 | 0.010 | HS_5_vs_HS_0 |
| LOC100299712 | 100299712 | None | 0.6 | 0.000 | 0.010 | HS_5_vs_HS_0 |
| PREX1 | 527410 | phosphatidylinositol-3,4,5-trisphosphate dependent Rac exchange factor 1 | -0.7 | 0.000 | 0.010 | HS_5_vs_HS_0 |
| LOC112441687 | 112441687 | U6 spliceosomal RNA | 1.8 | 0.000 | 0.010 | HS_5_vs_HS_0 |
| LOC112446710 | 112446710 | None | -1.6 | 0.000 | 0.010 | HS_5_vs_HS_0 |
| H4C8 | 527388 | H4 histone 16 | -1.3 | 0.000 | 0.010 | HS_5_vs_HS_0 |
| LOC132344835 | 132344835 | None | 1.0 | 0.000 | 0.010 | HS_5_vs_HS_0 |
| LOC112446509 | 112446509 | U1 spliceosomal RNA | -2.4 | 0.000 | 0.011 | HS_5_vs_HS_0 |
| IDUA | 511050 | None | -0.5 | 0.000 | 0.011 | HS_5_vs_HS_0 |
| EED | 404183 | embryonic ectoderm development | 0.6 | 0.000 | 0.011 | HS_5_vs_HS_0 |
| NAA80 | 104968404 | None | -0.8 | 0.000 | 0.011 | HS_5_vs_HS_0 |
| LYSMD2 | 511013 | None | 0.5 | 0.000 | 0.011 | HS_5_vs_HS_0 |
| TRIM36 | 539023 | tripartite motif containing 36 | 0.7 | 0.000 | 0.011 | HS_5_vs_HS_0 |
| STAG3 | 515399 | STAG3 cohesin complex component | -0.6 | 0.000 | 0.011 | HS_5_vs_HS_0 |
| H2BC20 | 506306 | H2B clustered histone 19 | -0.8 | 0.000 | 0.011 | HS_5_vs_HS_0 |
| H4C3 | 115945166 | H4 clustered histone 3 | -1.2 | 0.000 | 0.011 | HS_5_vs_HS_0 |
| ZNF677 | 525522 | None | 0.6 | 0.000 | 0.011 | HS_5_vs_HS_0 |
| COPRS | 506999 | coordinator of PRMT5 and differentiation stimulator | -0.5 | 0.000 | 0.011 | HS_5_vs_HS_0 |
| LOC112442853 | 112442853 | U2 spliceosomal RNA | -2.2 | 0.000 | 0.011 | HS_5_vs_HS_0 |
| CFL2 | 539332 | cofilin 2 | 0.7 | 0.000 | 0.012 | HS_5_vs_HS_0 |
| H3C1 | 517139 | H3 clustered histone 6 | -1.3 | 0.000 | 0.012 | HS_5_vs_HS_0 |
| ZNF165 | 506398 | zinc finger protein 165 | 1.0 | 0.000 | 0.012 | HS_5_vs_HS_0 |
| ANKRD37 | 509139 | ankyrin repeat domain 37 | 0.9 | 0.000 | 0.012 | HS_5_vs_HS_0 |
| KLHDC2 | 535436 | kelch domain containing 2 | 0.7 | 0.000 | 0.012 | HS_5_vs_HS_0 |
| FAM25A | 781772 | None | -0.9 | 0.000 | 0.012 | HS_5_vs_HS_0 |
| CHORDC1 | 505144 | cysteine and histidine rich domain containing 1 | 0.8 | 0.000 | 0.012 | HS_5_vs_HS_0 |
| PHF6 | 518186 | PHD finger protein 6 | 0.7 | 0.000 | 0.012 | HS_5_vs_HS_0 |
| MYL5 | 101907739 | None | -0.8 | 0.000 | 0.012 | HS_5_vs_HS_0 |
| PTMS | 613777 | parathymosin | -0.9 | 0.000 | 0.012 | HS_5_vs_HS_0 |
| IGF2R | 281849 | insulin like growth factor 2 receptor | -0.7 | 0.000 | 0.012 | HS_5_vs_HS_0 |
| CDH3 | 281063 | cadherin 3 | -0.7 | 0.000 | 0.012 | HS_5_vs_HS_0 |
| NME4 | 789324 | None | -1.1 | 0.000 | 0.012 | HS_5_vs_HS_0 |
| COQ4 | 511987 | coenzyme Q4 | -0.5 | 0.000 | 0.012 | HS_5_vs_HS_0 |
| LOC112446653 | 112446653 | None | -1.0 | 0.000 | 0.012 | HS_5_vs_HS_0 |
| PARP10 | 510991 | poly(ADP-ribose) polymerase family member 10 | -0.6 | 0.000 | 0.013 | HS_5_vs_HS_0 |
| CPNE5 | 508482 | copine 5 | -1.2 | 0.000 | 0.013 | HS_5_vs_HS_0 |
| LOC112443614 | 112443614 | U1 spliceosomal RNA | -2.1 | 0.000 | 0.013 | HS_5_vs_HS_0 |
| TMEM234 | 100126052 | transmembrane protein 234 | -0.4 | 0.000 | 0.013 | HS_5_vs_HS_0 |
| LOC112441904 | 112441904 | Small nucleolar RNA SNORD78 | -1.2 | 0.000 | 0.013 | HS_5_vs_HS_0 |
| CNTNAP1 | 540997 | contactin associated protein 1 | -1.6 | 0.000 | 0.013 | HS_5_vs_HS_0 |
| LOC112442847 | 112442847 | U2 spliceosomal RNA | -2.3 | 0.000 | 0.013 | HS_5_vs_HS_0 |
| SLC6A6 | 282366 | solute carrier family 6 member 6 | -0.7 | 0.000 | 0.013 | HS_5_vs_HS_0 |
| LOC115945165 | 115945165 | H3 clustered histone 6 | -1.2 | 0.000 | 0.013 | HS_5_vs_HS_0 |
| LOC132344462 | 132344462 | P2Y receptor family member 8 | -0.8 | 0.000 | 0.013 | HS_5_vs_HS_0 |
| TOMM70 | 507707 | translocase of outer mitochondrial membrane 70 | 0.4 | 0.000 | 0.013 | HS_5_vs_HS_0 |
| ETF1 | 541077 | eukaryotic translation termination factor 1 | 0.5 | 0.000 | 0.013 | HS_5_vs_HS_0 |
| LOC112449037 | 112449037 | None | -1.1 | 0.000 | 0.013 | HS_5_vs_HS_0 |
| H3C6 | 115945167 | H3 clustered histone 6 | -1.4 | 0.000 | 0.013 | HS_5_vs_HS_0 |
| EXD3 | 618437 | exonuclease 3'-5' domain containing 3 | -1.0 | 0.000 | 0.013 | HS_5_vs_HS_0 |
| H2AC8 | 524808 | None | -1.6 | 0.000 | 0.013 | HS_5_vs_HS_0 |
| RAB11FIP4 | 100848380 | RAB11 family interacting protein 4 | -0.7 | 0.000 | 0.013 | HS_5_vs_HS_0 |
| MMP24OS | 112449330 | None | -0.6 | 0.000 | 0.013 | HS_5_vs_HS_0 |
| AMBRA1 | 517263 | autophagy and beclin 1 regulator 1 | -0.6 | 0.000 | 0.013 | HS_5_vs_HS_0 |
| PDK2 | 524075 | pyruvate dehydrogenase kinase 2 | -0.6 | 0.000 | 0.013 | HS_5_vs_HS_0 |
| LOC132344794 | 132344794 | None | -1.5 | 0.000 | 0.013 | HS_5_vs_HS_0 |
| ZDHHC21 | 535814 | zinc finger DHHC-type palmitoyltransferase 21 | 0.5 | 0.000 | 0.013 | HS_5_vs_HS_0 |
| LOC790009 | 790009 | None | 0.6 | 0.000 | 0.013 | HS_5_vs_HS_0 |
| THA1 | 507443 | None | -0.6 | 0.000 | 0.013 | HS_5_vs_HS_0 |
| ABCD1 | 515178 | ATP binding cassette subfamily D member 1 | -0.8 | 0.000 | 0.013 | HS_5_vs_HS_0 |
| KCTD10 | 540881 | potassium channel tetramerization domain containing 10 | 0.6 | 0.000 | 0.013 | HS_5_vs_HS_0 |
| EFHD2 | 514259 | EF-hand domain family member D2 | -0.8 | 0.000 | 0.014 | HS_5_vs_HS_0 |
| LOC112442110 | 112442110 | U4 spliceosomal RNA | -1.6 | 0.000 | 0.014 | HS_5_vs_HS_0 |
| LOC112442852 | 112442852 | U2 spliceosomal RNA | -1.8 | 0.000 | 0.014 | HS_5_vs_HS_0 |
| SEC61A1 | 505064 | SEC61 translocon subunit alpha 1 | -0.6 | 0.000 | 0.014 | HS_5_vs_HS_0 |
| GABBR1 | 513785 | None | -0.9 | 0.000 | 0.014 | HS_5_vs_HS_0 |
| TUBGCP2 | 781376 | None | -0.5 | 0.000 | 0.014 | HS_5_vs_HS_0 |
| ADIRF | 613941 | None | -1.1 | 0.000 | 0.014 | HS_5_vs_HS_0 |
| OSTC | 768033 | oligosaccharyltransferase complex non-catalytic subunit | 0.4 | 0.000 | 0.014 | HS_5_vs_HS_0 |
| ADRB2 | 281605 | adrenoceptor beta 2 | 1.0 | 0.000 | 0.014 | HS_5_vs_HS_0 |
| GPR171 | 767929 | G protein-coupled receptor 171 | 0.7 | 0.000 | 0.014 | HS_5_vs_HS_0 |
| RBM42 | 540172 | RNA binding motif protein 42 | -0.4 | 0.000 | 0.014 | HS_5_vs_HS_0 |
| EFCAB7 | 505160 | EF-hand calcium binding domain 7 | 0.6 | 0.000 | 0.014 | HS_5_vs_HS_0 |
| NOCT | 540641 | nocturnin | 0.5 | 0.000 | 0.014 | HS_5_vs_HS_0 |
| LOC112442843 | 112442843 | U2 spliceosomal RNA | -1.9 | 0.000 | 0.015 | HS_5_vs_HS_0 |
| HS3ST6 | 540893 | None | -1.0 | 0.000 | 0.015 | HS_5_vs_HS_0 |
| LOC112446131 | 112446131 | U1 spliceosomal RNA | -2.2 | 0.000 | 0.015 | HS_5_vs_HS_0 |
| YKT6 | 507409 | None | -0.8 | 0.000 | 0.015 | HS_5_vs_HS_0 |
| LOC112448415 | 112448415 | None | -0.9 | 0.000 | 0.015 | HS_5_vs_HS_0 |
| LOC107132982 | 107132982 | None | 0.8 | 0.000 | 0.015 | HS_5_vs_HS_0 |
| P4HA1 | 518288 | prolyl 4-hydroxylase subunit alpha 1 | 0.6 | 0.000 | 0.015 | HS_5_vs_HS_0 |
| C3H1orf122 | 132344942 | None | -0.4 | 0.000 | 0.015 | HS_5_vs_HS_0 |
| TSTD3 | 782657 | thiosulfate sulfurtransferase like domain containing 3 | -0.3 | 0.000 | 0.015 | HS_5_vs_HS_0 |
| CRYBB1 | 282205 | crystallin beta B1 | -0.7 | 0.000 | 0.015 | HS_5_vs_HS_0 |
| CSTPP1 | 505437 | centriolar satellite-associated tubulin polyglutamylase complex regulator 1 | -1.1 | 0.000 | 0.015 | HS_5_vs_HS_0 |
| DCLRE1C | 517886 | None | 0.7 | 0.000 | 0.015 | HS_5_vs_HS_0 |
| C11H2orf16 | 100141098 | None | -1.4 | 0.000 | 0.015 | HS_5_vs_HS_0 |
| C18H16orf46 | 614002 | chromosome 18 C16orf46 homolog | 0.9 | 0.000 | 0.015 | HS_5_vs_HS_0 |
| ALDOA | 509566 | aldolase, fructose-bisphosphate A | -0.7 | 0.000 | 0.015 | HS_5_vs_HS_0 |
| SLC9A8 | 617800 | solute carrier family 9 member A8 | -0.6 | 0.000 | 0.016 | HS_5_vs_HS_0 |
| ATG2A | 529808 | autophagy related 2A | -1.0 | 0.000 | 0.016 | HS_5_vs_HS_0 |
| ZNF596 | 511959 | zinc finger protein 596 | 0.7 | 0.000 | 0.016 | HS_5_vs_HS_0 |
| NCK2 | 526430 | NCK adaptor protein 2 | 0.4 | 0.000 | 0.016 | HS_5_vs_HS_0 |
| CAPN10 | 789674 | calpain 10 | -0.6 | 0.000 | 0.016 | HS_5_vs_HS_0 |
| EMID1 | 506282 | EMI domain containing 1 | -0.7 | 0.000 | 0.016 | HS_5_vs_HS_0 |
| H1-3 | 509275 | None | -1.2 | 0.000 | 0.016 | HS_5_vs_HS_0 |
| LOC104976279 | 104976279 | None | -1.3 | 0.000 | 0.016 | HS_5_vs_HS_0 |
| NCBP1 | 100124428 | nuclear cap binding protein subunit 1 | 0.7 | 0.000 | 0.016 | HS_5_vs_HS_0 |
| CD40 | 286849 | CD40 molecule | 0.5 | 0.000 | 0.016 | HS_5_vs_HS_0 |
| ABL1 | 540876 | ABL proto-onco 1, non-receptor tyrosine kinase | -0.9 | 0.000 | 0.016 | HS_5_vs_HS_0 |
| ACTMAP | 509803 | actin maturation protease | -0.5 | 0.000 | 0.016 | HS_5_vs_HS_0 |
| LOC112442784 | 112442784 | U1 spliceosomal RNA | -1.9 | 0.000 | 0.016 | HS_5_vs_HS_0 |
| PLSCR3 | 510355 | phospholipid scramblase 3 | -0.5 | 0.000 | 0.016 | HS_5_vs_HS_0 |
| LOC100335608 | 100335608 | None | -0.6 | 0.000 | 0.016 | HS_5_vs_HS_0 |
| PIDD1 | 100137737 | p53-induced death domain protein 1 | -0.6 | 0.000 | 0.016 | HS_5_vs_HS_0 |
| DPM2 | 523737 | None | -0.5 | 0.000 | 0.016 | HS_5_vs_HS_0 |
| ANKRD63 | 100140532 | None | -1.0 | 0.000 | 0.016 | HS_5_vs_HS_0 |
| TBC1D8B | 100337282 | TBC1 domain family member 8B | 0.9 | 0.000 | 0.016 | HS_5_vs_HS_0 |
| BAG2 | 506107 | None | 0.7 | 0.000 | 0.016 | HS_5_vs_HS_0 |
| ADD1 | 507193 | adducin 1 | -0.6 | 0.000 | 0.017 | HS_5_vs_HS_0 |
| NBEAL2 | 788207 | None | -0.7 | 0.000 | 0.017 | HS_5_vs_HS_0 |
| LOC104974667 | 104974667 | None | -0.9 | 0.000 | 0.017 | HS_5_vs_HS_0 |
| PLEKHG2 | 504530 | pleckstrin homology and RhoGEF domain containing G2 | -0.7 | 0.000 | 0.017 | HS_5_vs_HS_0 |
| H3C8 | 115945169 | H3 clustered histone 6 | -1.3 | 0.000 | 0.017 | HS_5_vs_HS_0 |
| MSRB1 | 618441 | None | -0.9 | 0.000 | 0.017 | HS_5_vs_HS_0 |
| MIR484 | 790989 | bta-mir-484 | -0.8 | 0.000 | 0.017 | HS_5_vs_HS_0 |
| KIF3A | 541246 | kinesin family member 3A | 0.6 | 0.000 | 0.017 | HS_5_vs_HS_0 |
| TLE1 | 512888 | None | -0.5 | 0.000 | 0.017 | HS_5_vs_HS_0 |
| CHRNB3 | 521702 | cholinergic receptor nicotinic beta 3 subunit | -0.9 | 0.000 | 0.017 | HS_5_vs_HS_0 |
| LOC112446125 | 112446125 | U1 spliceosomal RNA | -2.5 | 0.000 | 0.017 | HS_5_vs_HS_0 |
| LOC101910192 | 101910192 | None | -1.8 | 0.000 | 0.017 | HS_5_vs_HS_0 |
| RYK | 781353 | None | 0.5 | 0.000 | 0.017 | HS_5_vs_HS_0 |
| MED15 | 618232 | mediator complex subunit 15 | -0.4 | 0.000 | 0.017 | HS_5_vs_HS_0 |
| SARS2 | 282060 | None | -0.4 | 0.000 | 0.017 | HS_5_vs_HS_0 |
| BANP | 513446 | None | 0.7 | 0.000 | 0.018 | HS_5_vs_HS_0 |
| H3C12 | 616800 | H3 clustered histone 6 | -1.5 | 0.000 | 0.018 | HS_5_vs_HS_0 |
| ZNF32 | 512392 | None | 0.3 | 0.000 | 0.018 | HS_5_vs_HS_0 |
| PAPOLG | 529071 | poly(A) polymerase gamma | 0.4 | 0.000 | 0.018 | HS_5_vs_HS_0 |
| LOC112443636 | 112443636 | U1 spliceosomal RNA | -2.0 | 0.000 | 0.018 | HS_5_vs_HS_0 |
| AFAP1 | 534032 | actin filament associated protein 1 | -0.8 | 0.000 | 0.018 | HS_5_vs_HS_0 |
| EFCAB11 | 617365 | None | -0.5 | 0.000 | 0.018 | HS_5_vs_HS_0 |
| FANCA | 618375 | FA complementation group A | -0.5 | 0.000 | 0.018 | HS_5_vs_HS_0 |
| BREH1 | 497207 | None | -1.1 | 0.000 | 0.018 | HS_5_vs_HS_0 |
| MRPS18B | 510824 | None | -0.4 | 0.000 | 0.018 | HS_5_vs_HS_0 |
| LOC115945171 | 115945171 | H3 clustered histone 6 | -1.4 | 0.000 | 0.018 | HS_5_vs_HS_0 |
| INTS5 | 514150 | integrator complex subunit 5 | -0.7 | 0.000 | 0.018 | HS_5_vs_HS_0 |
| L1CAM | 516017 | L1 cell adhesion molecule | -0.8 | 0.000 | 0.018 | HS_5_vs_HS_0 |
| ZNG1A | 100300568 | Zn regulated GTPase metalloprotein activator 1 | 0.6 | 0.000 | 0.018 | HS_5_vs_HS_0 |
| FKRP | 539701 | fukutin related protein | 0.6 | 0.000 | 0.018 | HS_5_vs_HS_0 |
| CR1L | 100138386 | None | 1.3 | 0.000 | 0.018 | HS_5_vs_HS_0 |
| BNIP2 | 516077 | BCL2 interacting protein 2 | 0.6 | 0.000 | 0.018 | HS_5_vs_HS_0 |
| ZBTB42 | 789062 | zinc finger and BTB domain containing 42 | -0.5 | 0.000 | 0.018 | HS_5_vs_HS_0 |
| PVRIG | 514026 | PVR related immunoglobulin domain containing | -0.7 | 0.000 | 0.018 | HS_5_vs_HS_0 |
| XRCC2 | 789410 | X-ray repair cross complementing 2 | 0.8 | 0.000 | 0.018 | HS_5_vs_HS_0 |
| TCF7 | 782690 | transcription factor 7 | -0.5 | 0.000 | 0.018 | HS_5_vs_HS_0 |
| SOX13 | 788459 | None | -1.0 | 0.000 | 0.018 | HS_5_vs_HS_0 |
| FXYD5 | 505584 | FXYD domain containing ion transport regulator 5 | -0.5 | 0.000 | 0.018 | HS_5_vs_HS_0 |
| LIME1 | 100140162 | None | -0.7 | 0.000 | 0.018 | HS_5_vs_HS_0 |
| LOC789148 | 789148 | RAP1B, member of RAS onco family | 1.0 | 0.000 | 0.018 | HS_5_vs_HS_0 |
| HINT2 | 281816 | histidine triad nucleotide binding protein 2 | -0.5 | 0.000 | 0.018 | HS_5_vs_HS_0 |
| UFD1 | 507124 | None | -0.4 | 0.000 | 0.018 | HS_5_vs_HS_0 |
| ARL6 | 519014 | ADP ribosylation factor like GTPase 6 | 0.8 | 0.000 | 0.018 | HS_5_vs_HS_0 |
| ARL8B | 511009 | ADP ribosylation factor like GTPase 8B | 0.7 | 0.000 | 0.018 | HS_5_vs_HS_0 |
| SPATA7 | 510352 | spermatosis associated 7 | -0.4 | 0.000 | 0.018 | HS_5_vs_HS_0 |
| TCP1 | 512043 | t-complex 1 | 0.7 | 0.000 | 0.018 | HS_5_vs_HS_0 |
| BCL2L12 | 533338 | BCL2 like 12 | -0.5 | 0.000 | 0.019 | HS_5_vs_HS_0 |
| YBEY | 768063 | None | -0.5 | 0.000 | 0.019 | HS_5_vs_HS_0 |
| STK11IP | 515395 | serine/threonine kinase 11 interacting protein | -0.5 | 0.000 | 0.019 | HS_5_vs_HS_0 |
| ENTPD2 | 100126045 | ectonucleoside triphosphate diphosphohydrolase 2 | -0.7 | 0.000 | 0.019 | HS_5_vs_HS_0 |
| PRPF6 | 534228 | pre-mRNA processing factor 6 | -0.7 | 0.000 | 0.019 | HS_5_vs_HS_0 |
| ALYREF | 537706 | Aly/REF export factor | -0.5 | 0.000 | 0.019 | HS_5_vs_HS_0 |
| LOC132343370 | 132343370 | None | -1.7 | 0.000 | 0.019 | HS_5_vs_HS_0 |
| CCDC12 | 617519 | coiled-coil domain containing 12 | -0.3 | 0.000 | 0.019 | HS_5_vs_HS_0 |
| TNXB | 282654 | None | -1.0 | 0.000 | 0.019 | HS_5_vs_HS_0 |
| LOC107131750 | 107131750 | H3 clustered histone 6 | -1.1 | 0.000 | 0.019 | HS_5_vs_HS_0 |
| SIGIRR | 531801 | None | -0.5 | 0.000 | 0.019 | HS_5_vs_HS_0 |
| LOC617475 | 617475 | None | -0.7 | 0.000 | 0.019 | HS_5_vs_HS_0 |
| GDAP2 | 508774 | ganglioside induced differentiation associated protein 2 | 0.6 | 0.000 | 0.019 | HS_5_vs_HS_0 |
| C9orf72 | 506309 | None | 0.8 | 0.000 | 0.019 | HS_5_vs_HS_0 |
| GNPNAT1 | 512299 | glucosamine-phosphate N-acetyltransferase 1 | 0.6 | 0.000 | 0.019 | HS_5_vs_HS_0 |
| RAD54B | 533414 | None | 0.7 | 0.000 | 0.019 | HS_5_vs_HS_0 |
| H2AC13 | 783767 | None | -1.4 | 0.000 | 0.019 | HS_5_vs_HS_0 |
| RABIF | 616779 | RAB interacting factor | -0.7 | 0.000 | 0.019 | HS_5_vs_HS_0 |
| E2F5 | 539427 | None | 0.7 | 0.000 | 0.019 | HS_5_vs_HS_0 |
| CMAH | 537017 | None | 0.4 | 0.000 | 0.019 | HS_5_vs_HS_0 |
| SPATA32 | 767871 | None | -2.8 | 0.000 | 0.019 | HS_5_vs_HS_0 |
| STUB1 | 504565 | None | -1.3 | 0.000 | 0.019 | HS_5_vs_HS_0 |
| LOC112444276 | 112444276 | None | -0.5 | 0.000 | 0.019 | HS_5_vs_HS_0 |
| BOLA1 | 509530 | bolA family member 1 | -0.6 | 0.000 | 0.019 | HS_5_vs_HS_0 |
| ACAP3 | 515444 | ArfGAP with coiled-coil, ankyrin repeat and PH domains 3 | -1.0 | 0.000 | 0.020 | HS_5_vs_HS_0 |
| EFNB1 | 534413 | ephrin B1 | -1.0 | 0.000 | 0.020 | HS_5_vs_HS_0 |
| LOC505306 | 505306 | None | -1.3 | 0.000 | 0.020 | HS_5_vs_HS_0 |
| ALAS2 | 511791 | 5'-aminolevulinate synthase 2 | -1.1 | 0.000 | 0.020 | HS_5_vs_HS_0 |
| SMPD4 | 507207 | sphingomyelin phosphodiesterase 4 | -0.7 | 0.000 | 0.020 | HS_5_vs_HS_0 |
| PTGS2 | 282023 | prostaglandin-endoperoxide synthase 2 | 1.4 | 0.000 | 0.020 | HS_5_vs_HS_0 |
| FBXL20 | 511007 | F-box and leucine rich repeat protein 20 | 0.5 | 0.000 | 0.020 | HS_5_vs_HS_0 |
| LOC101904947 | 101904947 | None | -0.4 | 0.000 | 0.020 | HS_5_vs_HS_0 |
| PODNL1 | 534549 | podocan like 1 | -0.6 | 0.000 | 0.020 | HS_5_vs_HS_0 |
| LOC101905262 | 101905262 | None | -0.7 | 0.000 | 0.020 | HS_5_vs_HS_0 |
| PLCB2 | 508888 | phospholipase C beta 2 | -0.4 | 0.000 | 0.020 | HS_5_vs_HS_0 |
| FAM83H | 524974 | family with sequence similarity 83 member H | -0.8 | 0.000 | 0.020 | HS_5_vs_HS_0 |
| GAS8 | 504318 | None | -0.5 | 0.000 | 0.020 | HS_5_vs_HS_0 |
| CCNK | 530744 | cyclin K | -0.9 | 0.000 | 0.020 | HS_5_vs_HS_0 |
| LOC112444214 | 112444214 | small Cajal body-specific RNA 18 | -0.8 | 0.000 | 0.020 | HS_5_vs_HS_0 |
| EPB41L4B | 519310 | erythrocyte membrane protein band 4.1 like 4B | 0.6 | 0.000 | 0.020 | HS_5_vs_HS_0 |
| KCNE3 | 527762 | None | 1.0 | 0.000 | 0.020 | HS_5_vs_HS_0 |
| ADPRM | 534038 | None | 0.5 | 0.000 | 0.020 | HS_5_vs_HS_0 |
| DAPK1 | 540873 | None | -0.6 | 0.000 | 0.020 | HS_5_vs_HS_0 |
| MYH9 | 404108 | myosin heavy chain 9 | -0.7 | 0.000 | 0.021 | HS_5_vs_HS_0 |
| PRSS50 | 518845 | None | -1.1 | 0.000 | 0.021 | HS_5_vs_HS_0 |
| GLT8D1 | 512192 | glycosyltransferase 8 domain containing 1 | 0.5 | 0.000 | 0.021 | HS_5_vs_HS_0 |
| NUDT13 | 504993 | nudix hydrolase 13 | 1.0 | 0.000 | 0.021 | HS_5_vs_HS_0 |
| LOC112449590 | 112449590 | None | -0.8 | 0.000 | 0.021 | HS_5_vs_HS_0 |
| GSTZ1 | 514822 | glutathione S-transferase zeta 1 | -0.7 | 0.000 | 0.021 | HS_5_vs_HS_0 |
| CHAC2 | 511605 | ChaC glutathione specific gamma-glutamylcyclotransferase 2 | 0.5 | 0.000 | 0.021 | HS_5_vs_HS_0 |
| LOC132344829 | 132344829 | None | -0.7 | 0.000 | 0.021 | HS_5_vs_HS_0 |
| CDH10 | 541259 | cadherin 10 | 1.5 | 0.000 | 0.022 | HS_5_vs_HS_0 |
| LOC132342072 | 132342072 | None | -0.6 | 0.000 | 0.022 | HS_5_vs_HS_0 |
| SUGP1 | 533971 | SURP and G-patch domain containing 1 | -0.3 | 0.000 | 0.022 | HS_5_vs_HS_0 |
| LOC101902444 | 101902444 | None | -0.6 | 0.000 | 0.022 | HS_5_vs_HS_0 |
| LOC132342368 | 132342368 | None | -0.7 | 0.001 | 0.022 | HS_5_vs_HS_0 |
| LOC101905357 | 101905357 | None | -0.9 | 0.000 | 0.022 | HS_5_vs_HS_0 |
| H3C2 | 788250 | H3 clustered histone 6 | -1.0 | 0.000 | 0.022 | HS_5_vs_HS_0 |
| TYSND1 | 101903772 | None | -0.5 | 0.001 | 0.022 | HS_5_vs_HS_0 |
| PCNX4 | 539495 | None | 0.5 | 0.000 | 0.022 | HS_5_vs_HS_0 |
| ZNF383 | 112442193 | zinc finger protein 383 | 0.7 | 0.000 | 0.022 | HS_5_vs_HS_0 |
| COX15 | 517811 | cytochrome c oxidase assembly homolog COX15 | 0.5 | 0.000 | 0.022 | HS_5_vs_HS_0 |
| GPR35 | 505056 | G protein-coupled receptor 35 | -0.5 | 0.001 | 0.022 | HS_5_vs_HS_0 |
| H4C16 | 530773 | H4 histone 16 | -1.0 | 0.001 | 0.022 | HS_5_vs_HS_0 |
| RGMB | 540954 | repulsive guidance molecule BMP co-receptor b | 1.0 | 0.001 | 0.022 | HS_5_vs_HS_0 |
| LOC100847495 | 100847495 | None | 0.6 | 0.001 | 0.022 | HS_5_vs_HS_0 |
| LZTS1 | 539634 | leucine zipper tumor suppressor 1 | -1.0 | 0.001 | 0.022 | HS_5_vs_HS_0 |
| H2AC4 | 104975684 | None | -1.2 | 0.001 | 0.022 | HS_5_vs_HS_0 |
| ZCWPW1 | 514550 | None | -0.8 | 0.001 | 0.022 | HS_5_vs_HS_0 |
| RNF187 | 618753 | ring finger protein 187 | -0.4 | 0.001 | 0.022 | HS_5_vs_HS_0 |
| HSF1 | 506235 | heat shock transcription factor 1 | -0.7 | 0.001 | 0.022 | HS_5_vs_HS_0 |
| LOC112444215 | 112444215 | small Cajal body-specific RNA 17 | -0.8 | 0.001 | 0.022 | HS_5_vs_HS_0 |
| RNPEPL1 | 511497 | arginyl aminopeptidase like 1 | -0.7 | 0.001 | 0.023 | HS_5_vs_HS_0 |
| LOC132345555 | 132345555 | None | -0.4 | 0.001 | 0.023 | HS_5_vs_HS_0 |
| HP1BP3 | 510194 | heterochromatin protein 1 binding protein 3 | -0.3 | 0.001 | 0.023 | HS_5_vs_HS_0 |
| NUMA1 | 513091 | None | -0.5 | 0.001 | 0.023 | HS_5_vs_HS_0 |
| C1QTNF12 | 506102 | C1q and TNF related 12 | -0.4 | 0.001 | 0.023 | HS_5_vs_HS_0 |
| ALKBH6 | 539257 | None | -0.6 | 0.001 | 0.023 | HS_5_vs_HS_0 |
| LOC112442858 | 112442858 | U2 spliceosomal RNA | -1.6 | 0.001 | 0.023 | HS_5_vs_HS_0 |
| SLC25A10 | 100848134 | solute carrier family 25 member 10 | -0.5 | 0.001 | 0.023 | HS_5_vs_HS_0 |
| PLXNB1 | 616798 | plexin B1 | -0.9 | 0.001 | 0.023 | HS_5_vs_HS_0 |
| PLXND1 | 781625 | plexin D1 | -1.2 | 0.001 | 0.023 | HS_5_vs_HS_0 |
| H4C12 | 115945172 | H4 histone 16 | -1.1 | 0.001 | 0.023 | HS_5_vs_HS_0 |
| LOC112444222 | 112444222 | U2 spliceosomal RNA | -1.9 | 0.001 | 0.023 | HS_5_vs_HS_0 |
| ITGAL | 281874 | integrin subunit alpha L | -0.7 | 0.001 | 0.023 | HS_5_vs_HS_0 |
| GNB2 | 281202 | G protein subunit beta 2 | -0.4 | 0.001 | 0.023 | HS_5_vs_HS_0 |
| TSPAN14 | 539055 | tetraspanin 14 | -0.4 | 0.001 | 0.023 | HS_5_vs_HS_0 |
| ZFPL1 | 613442 | zinc finger protein like 1 | -2.2 | 0.001 | 0.023 | HS_5_vs_HS_0 |
| RPF1 | 513081 | ribosome production factor 1 homolog | 0.5 | 0.001 | 0.023 | HS_5_vs_HS_0 |
| SRP14 | 512792 | signal recognition particle 14 | 0.4 | 0.001 | 0.023 | HS_5_vs_HS_0 |
| ART1 | 539042 | ADP-ribosyltransferase 1 | 1.1 | 0.001 | 0.023 | HS_5_vs_HS_0 |
| PPID | 281420 | peptidylprolyl isomerase D | 0.5 | 0.001 | 0.023 | HS_5_vs_HS_0 |
| GLO1 | 540335 | glyoxalase I | 0.5 | 0.001 | 0.023 | HS_5_vs_HS_0 |
| STIP1 | 617109 | stress induced phosphoprotein 1 | 0.5 | 0.001 | 0.023 | HS_5_vs_HS_0 |
| PCBD1 | 530736 | None | 0.6 | 0.001 | 0.023 | HS_5_vs_HS_0 |
| IZUMO4 | 510569 | IZUMO family member 4 | -1.0 | 0.001 | 0.023 | HS_5_vs_HS_0 |
| MSRB2 | 613475 | None | -0.5 | 0.001 | 0.023 | HS_5_vs_HS_0 |
| CENPT | 513195 | centromere protein T | -0.4 | 0.001 | 0.023 | HS_5_vs_HS_0 |
| LOC132343352 | 132343352 | None | -0.9 | 0.001 | 0.023 | HS_5_vs_HS_0 |
| PPP1R10 | 510825 | protein phosphatase 1 regulatory subunit 10 | -0.6 | 0.001 | 0.023 | HS_5_vs_HS_0 |
| PYGM | 327664 | glycogen phosphorylase, muscle associated | -0.7 | 0.001 | 0.023 | HS_5_vs_HS_0 |
| UNC50 | 513629 | None | 0.5 | 0.001 | 0.023 | HS_5_vs_HS_0 |
| LOC132346917 | 132346917 | None | 0.9 | 0.001 | 0.023 | HS_5_vs_HS_0 |
| HYKK | 530270 | hydroxylysine kinase | 1.0 | 0.001 | 0.023 | HS_5_vs_HS_0 |
| LOC101904090 | 101904090 | None | -1.2 | 0.001 | 0.023 | HS_5_vs_HS_0 |
| VPS28 | 618785 | None | -0.6 | 0.001 | 0.023 | HS_5_vs_HS_0 |
| PRKD2 | 782793 | protein kinase D2 | -0.7 | 0.001 | 0.023 | HS_5_vs_HS_0 |
| DNAJB9 | 614588 | DnaJ heat shock protein family (Hsp40) member B9 | 0.9 | 0.001 | 0.023 | HS_5_vs_HS_0 |
| LOC101906008 | 101906008 | None | -0.9 | 0.001 | 0.023 | HS_5_vs_HS_0 |
| MTX2 | 615424 | None | 0.6 | 0.001 | 0.023 | HS_5_vs_HS_0 |
| MYL6B | 515421 | myosin light chain 6B | -0.5 | 0.001 | 0.023 | HS_5_vs_HS_0 |
| CDCA3 | 614434 | cell division cycle associated 3 | -0.5 | 0.001 | 0.023 | HS_5_vs_HS_0 |
| PABPC4 | 534576 | None | -0.6 | 0.001 | 0.023 | HS_5_vs_HS_0 |
| ZBTB43 | 509462 | zinc finger and BTB domain containing 43 | 0.6 | 0.001 | 0.023 | HS_5_vs_HS_0 |
| LCORL | 540095 | None | 0.5 | 0.001 | 0.023 | HS_5_vs_HS_0 |
| ORC6 | 515476 | origin recognition complex subunit 6 | 0.5 | 0.001 | 0.023 | HS_5_vs_HS_0 |
| ELOVL5 | 617293 | ELOVL fatty acid elongase 5 | 0.6 | 0.001 | 0.023 | HS_5_vs_HS_0 |
| LOC787234 |  |  | 1.1 | 0.001 | 0.023 | HS_5_vs_HS_0 |
| LOC507787 | 507787 | None | 0.7 | 0.001 | 0.023 | HS_5_vs_HS_0 |
| ABCE1 | 514991 | ATP binding cassette subfamily E member 1 | 0.5 | 0.001 | 0.023 | HS_5_vs_HS_0 |
| EXOC8 | 540237 | exocyst complex component 8 | 0.6 | 0.001 | 0.023 | HS_5_vs_HS_0 |
| ALKBH3 | 514579 | alkB homolog 3, alpha-ketoglutarate dependent dioxygenase | -0.4 | 0.001 | 0.024 | HS_5_vs_HS_0 |
| ADCYAP1R1 | 319095 | ADCYAP receptor type I | -0.9 | 0.001 | 0.024 | HS_5_vs_HS_0 |
| LOC112449350 | 112449350 | None | -1.4 | 0.001 | 0.024 | HS_5_vs_HS_0 |
| POLR1G | 617937 | RNA polymerase I subunit G | -0.4 | 0.001 | 0.024 | HS_5_vs_HS_0 |
| CBX4 | 767865 | chromobox 4 | -0.4 | 0.001 | 0.024 | HS_5_vs_HS_0 |
| H2AC16 | 529277 | H2A clustered histone 14 | -1.6 | 0.001 | 0.024 | HS_5_vs_HS_0 |
| DHX15 | 512327 | DEAH-box helicase 15 | 0.4 | 0.001 | 0.024 | HS_5_vs_HS_0 |
| LOC112449602 | 112449602 | None | -0.5 | 0.001 | 0.024 | HS_5_vs_HS_0 |
| COMMD8 | 507292 | COMM domain containing 8 | 0.4 | 0.001 | 0.024 | HS_5_vs_HS_0 |
| LOC112447188 | 112447188 | small Cajal body-specific RNA 18 | -1.0 | 0.001 | 0.024 | HS_5_vs_HS_0 |
| LOC132346328 | 132346328 | None | -1.2 | 0.001 | 0.024 | HS_5_vs_HS_0 |
| COPS3 | 507932 | COP9 signalosome subunit 3 | -0.3 | 0.001 | 0.024 | HS_5_vs_HS_0 |
| ATXN7L3 | 525252 | ataxin 7 like 3 | -0.7 | 0.001 | 0.024 | HS_5_vs_HS_0 |
| ZP3 | 280964 | zona pellucida glycoprotein 3 | -0.7 | 0.001 | 0.024 | HS_5_vs_HS_0 |
| G6PD | 281179 | glucose-6-phosphate dehydrogenase | -1.1 | 0.001 | 0.024 | HS_5_vs_HS_0 |
| FBXO36 | 617339 | F-box protein 36 | -1.2 | 0.001 | 0.024 | HS_5_vs_HS_0 |
| LOC510185 | 510185 | None | 0.8 | 0.001 | 0.024 | HS_5_vs_HS_0 |
| CLECL1 | 618591 | C-type lectin like 1 | 1.0 | 0.001 | 0.024 | HS_5_vs_HS_0 |
| USE1 | 512890 | None | -0.5 | 0.001 | 0.024 | HS_5_vs_HS_0 |
| LOC100140403 | 100140403 | None | -1.3 | 0.001 | 0.024 | HS_5_vs_HS_0 |
| LOC101902944 | 101902944 | None | -0.9 | 0.001 | 0.024 | HS_5_vs_HS_0 |
| INO80C | 533426 | INO80 complex subunit C | -0.4 | 0.001 | 0.024 | HS_5_vs_HS_0 |
| ZNF48 | 512477 | zinc finger protein 48 | -0.8 | 0.001 | 0.024 | HS_5_vs_HS_0 |
| ETFBKMT | 530461 | electron transfer flavoprotein subunit beta lysine methyltransferase | 0.5 | 0.001 | 0.024 | HS_5_vs_HS_0 |
| ZNF239 | 100848535 | None | 0.5 | 0.001 | 0.024 | HS_5_vs_HS_0 |
| TOR4A | 618444 | torsin family 4 member A | -0.5 | 0.001 | 0.025 | HS_5_vs_HS_0 |
| ARHGAP1 | 512817 | Rho GTPase activating protein 1 | -0.5 | 0.001 | 0.025 | HS_5_vs_HS_0 |
| LOC112443180 | 112443180 | None | -0.9 | 0.001 | 0.025 | HS_5_vs_HS_0 |
| OSTN | 511114 | osteocrin | 1.2 | 0.001 | 0.025 | HS_5_vs_HS_0 |
| STARD4 | 100847750 | None | 0.5 | 0.001 | 0.025 | HS_5_vs_HS_0 |
| STARD4 | 132342078 | StAR related lipid transfer domain containing 4 | 0.5 | 0.001 | 0.025 | HS_5_vs_HS_0 |
| TNFSF15 | 514239 | TNF superfamily member 15 | 0.9 | 0.001 | 0.025 | HS_5_vs_HS_0 |
| BABAM2 | 614152 | BRISC and BRCA1 A complex member 2 | -0.3 | 0.001 | 0.025 | HS_5_vs_HS_0 |
| TMEM94 | 512110 | transmembrane protein 94 | -0.4 | 0.001 | 0.025 | HS_5_vs_HS_0 |
| LOC786726 | 786726 | None | 0.7 | 0.001 | 0.025 | HS_5_vs_HS_0 |
| SNX11 | 534567 | sorting nexin 11 | -1.2 | 0.001 | 0.025 | HS_5_vs_HS_0 |
| NMNAT1 | 522863 | nicotinamide nucleotide adenylyltransferase 1 | 0.7 | 0.001 | 0.025 | HS_5_vs_HS_0 |
| EME2 | 515662 | None | -0.5 | 0.001 | 0.025 | HS_5_vs_HS_0 |
| LOC619094 | 619094 | None | -1.4 | 0.001 | 0.025 | HS_5_vs_HS_0 |
| SAMD10 | 540999 | None | -0.6 | 0.001 | 0.025 | HS_5_vs_HS_0 |
| TRAF3 | 506182 | TNF receptor associated factor 3 | -0.4 | 0.001 | 0.025 | HS_5_vs_HS_0 |
| PAIP1 | 510246 | poly(A) binding protein interacting protein 1 | 0.5 | 0.001 | 0.025 | HS_5_vs_HS_0 |
| ELOVL4 | 532015 | ELOVL fatty acid elongase 4 | 0.9 | 0.001 | 0.025 | HS_5_vs_HS_0 |
| SEC14L2 | 282469 | SEC14 like lipid binding 2 | -0.8 | 0.001 | 0.025 | HS_5_vs_HS_0 |
| RAB8A | 100125881 | RAB8A, member RAS onco family | -0.8 | 0.001 | 0.025 | HS_5_vs_HS_0 |
| MAST1 | 539825 | microtubule associated serine/threonine kinase 1 | -0.9 | 0.001 | 0.025 | HS_5_vs_HS_0 |
| LOC112448627 | 112448627 | U1 spliceosomal RNA | -1.7 | 0.001 | 0.025 | HS_5_vs_HS_0 |
| PRR3 | 525344 | proline rich 3 | -0.4 | 0.001 | 0.025 | HS_5_vs_HS_0 |
| H2BC9 | 104968456 | H2B clustered histone 19 | -0.7 | 0.001 | 0.025 | HS_5_vs_HS_0 |
| TNRC18 | 526616 | trinucleotide repeat containing 18 | -0.8 | 0.001 | 0.025 | HS_5_vs_HS_0 |
| UQCRB | 616871 | ubiquinol-cytochrome c reductase binding protein | 0.5 | 0.001 | 0.025 | HS_5_vs_HS_0 |
| BOD1 | 540063 | None | 0.5 | 0.001 | 0.025 | HS_5_vs_HS_0 |
| CEP20 | 782821 | myosin heavy chain 11 | 0.4 | 0.001 | 0.025 | HS_5_vs_HS_0 |
| TAF4 | 789854 | TATA-box binding protein associated factor 4 | -0.7 | 0.001 | 0.025 | HS_5_vs_HS_0 |
| TCF25 | 100848970 | transcription factor 25 | -0.5 | 0.001 | 0.025 | HS_5_vs_HS_0 |
| ZFP28 | 100301148 | ZFP28 zinc finger protein | 0.5 | 0.001 | 0.025 | HS_5_vs_HS_0 |
| TMEM126B | 504229 | transmembrane protein 126B | 0.4 | 0.001 | 0.025 | HS_5_vs_HS_0 |
| KCNH8 | 100336609 | potassium voltage-gated channel subfamily H member 8 | -0.9 | 0.001 | 0.025 | HS_5_vs_HS_0 |
| SH2D1B | 617485 | SH2 domain containing 1B | 0.6 | 0.001 | 0.026 | HS_5_vs_HS_0 |
| PDCD10 | 506411 | programmed cell death 10 | 0.4 | 0.001 | 0.026 | HS_5_vs_HS_0 |
| GPR52 | 506159 | None | -1.5 | 0.001 | 0.026 | HS_5_vs_HS_0 |
| LOC101903068 | 101903068 | None | -0.9 | 0.001 | 0.026 | HS_5_vs_HS_0 |
| H4C6 | 115945168 | H4 histone 16 | -1.2 | 0.001 | 0.026 | HS_5_vs_HS_0 |
| TCAM1 | 616168 | testicular cell adhesion molecule 1 | 1.1 | 0.001 | 0.026 | HS_5_vs_HS_0 |
| OGDH | 534599 | None | -0.6 | 0.001 | 0.026 | HS_5_vs_HS_0 |
| ILF3 | 614936 | interleukin enhancer binding factor 3 | -0.5 | 0.001 | 0.026 | HS_5_vs_HS_0 |
| GSK3A | 536561 | None | -0.4 | 0.001 | 0.026 | HS_5_vs_HS_0 |
| MIR339A | 100313035 | bta-mir-339a | -0.7 | 0.001 | 0.026 | HS_5_vs_HS_0 |
| PGLYRP2 | 510803 | None | -0.6 | 0.001 | 0.026 | HS_5_vs_HS_0 |
| DBN1 | 505406 | drebrin 1 | -0.6 | 0.001 | 0.026 | HS_5_vs_HS_0 |
| ARRB2 | 281638 | None | -0.5 | 0.001 | 0.026 | HS_5_vs_HS_0 |
| TADA1 | 521918 | transcriptional adaptor 1 | 0.5 | 0.001 | 0.026 | HS_5_vs_HS_0 |
| P2RX3 | 530468 | purinergic receptor P2X 3 | -0.6 | 0.001 | 0.026 | HS_5_vs_HS_0 |
| LOC112442856 | 112442856 | U2 spliceosomal RNA | -2.2 | 0.001 | 0.026 | HS_5_vs_HS_0 |
| KCND1 | 518384 | potassium voltage-gated channel subfamily D member 1 | -0.5 | 0.001 | 0.026 | HS_5_vs_HS_0 |
| LIMS2 | 515401 | LIM zinc finger domain containing 2 | -0.8 | 0.001 | 0.026 | HS_5_vs_HS_0 |
| FAAP20 | 508039 | None | -0.6 | 0.001 | 0.026 | HS_5_vs_HS_0 |
| UCP2 | 281562 | uncoupling protein 2 | -0.7 | 0.001 | 0.026 | HS_5_vs_HS_0 |
| WRAP53 | 509631 | None | -0.3 | 0.001 | 0.026 | HS_5_vs_HS_0 |
| LOC112445076 | 112445076 | None | -0.9 | 0.001 | 0.026 | HS_5_vs_HS_0 |
| C29H11orf54 | 530784 | chromosome 29 C11orf54 homolog | 0.6 | 0.001 | 0.026 | HS_5_vs_HS_0 |
| U2AF1L4 | 615198 | U2 small nuclear RNA auxiliary factor 1 like 4 | -0.4 | 0.001 | 0.026 | HS_5_vs_HS_0 |
| TUBB3 | 768070 | None | -0.6 | 0.001 | 0.027 | HS_5_vs_HS_0 |
| MLXIP | 783217 | MLX interacting protein | -0.5 | 0.001 | 0.027 | HS_5_vs_HS_0 |
| NICN1 | 614730 | nicolin 1, tubulin polyglutamylase complex subunit | -0.3 | 0.001 | 0.027 | HS_5_vs_HS_0 |
| H4C2 | 280691 | H4 histone 16 | -1.0 | 0.001 | 0.027 | HS_5_vs_HS_0 |
| GMDS | 617688 | GDP-mannose 4,6-dehydratase | -0.5 | 0.001 | 0.027 | HS_5_vs_HS_0 |
| LOC112444896 | 112444896 | None | 3.3 | 0.001 | 0.027 | HS_5_vs_HS_0 |
| TCTA | 616169 | None | -0.3 | 0.001 | 0.027 | HS_5_vs_HS_0 |
| CPSF7 | 504925 | cleavage and polyadenylation specific factor 7 | -0.5 | 0.001 | 0.027 | HS_5_vs_HS_0 |
| UBXN1 | 506676 | UBX domain protein 1 | -0.4 | 0.001 | 0.027 | HS_5_vs_HS_0 |
| CD2BP2 | 513857 | CD2 cytoplasmic tail binding protein 2 | -0.3 | 0.001 | 0.027 | HS_5_vs_HS_0 |
| XRRA1 | 369019 | X-ray radiation resistance associated 1 | -0.8 | 0.001 | 0.027 | HS_5_vs_HS_0 |
| DDT | 615999 | None | -0.6 | 0.001 | 0.027 | HS_5_vs_HS_0 |
| THOC3 | 529231 | THO complex subunit 3 | 0.4 | 0.001 | 0.027 | HS_5_vs_HS_0 |
| FICD | 505923 | FIC domain protein adenylyltransferase | 0.5 | 0.001 | 0.027 | HS_5_vs_HS_0 |
| FBRS | 615050 | fibrosin | -0.6 | 0.001 | 0.027 | HS_5_vs_HS_0 |
| C1GALT1 | 539417 | core 1 synthase, glycoprotein-N-acetylgalactosamine 3-beta-galactosyltransferase 1 | 0.7 | 0.001 | 0.027 | HS_5_vs_HS_0 |
| PIANP | 614601 | PILR alpha associated neural protein | -0.7 | 0.001 | 0.028 | HS_5_vs_HS_0 |
| FBXO48 | 786890 | F-box protein 48 | 0.9 | 0.001 | 0.028 | HS_5_vs_HS_0 |
| NDST2 | 286838 | N-deacetylase and N-sulfotransferase 2 | 0.5 | 0.001 | 0.028 | HS_5_vs_HS_0 |
| AKNA | 539274 | AT-hook transcription factor | -0.4 | 0.001 | 0.028 | HS_5_vs_HS_0 |
| LOC132343398 | 132343398 | None | -0.6 | 0.001 | 0.028 | HS_5_vs_HS_0 |
| ZNF226 | 789006 | None | 0.6 | 0.001 | 0.028 | HS_5_vs_HS_0 |
| HSH2D | 100848273 | None | -0.5 | 0.001 | 0.028 | HS_5_vs_HS_0 |
| QSOX1 | 522986 | None | -0.5 | 0.001 | 0.028 | HS_5_vs_HS_0 |
| H3C13 | 504599 | H3 clustered histone 13 | -1.0 | 0.001 | 0.028 | HS_5_vs_HS_0 |
| TP73 | 515105 | tumor protein p73 | -0.5 | 0.001 | 0.028 | HS_5_vs_HS_0 |
| C25H7orf50 | 522840 | chromosome 25 C7orf50 homolog | -0.4 | 0.001 | 0.028 | HS_5_vs_HS_0 |
| TMEM260 | 614796 | transmembrane protein 260 | 0.5 | 0.001 | 0.028 | HS_5_vs_HS_0 |
| C3H1orf52 | 509947 | None | 0.3 | 0.001 | 0.028 | HS_5_vs_HS_0 |
| CYSRT1 | 618457 | cysteine rich tail 1 | -1.2 | 0.001 | 0.028 | HS_5_vs_HS_0 |
| CNTROB | 539106 | centrobin, centriole duplication and spindle assembly protein | -0.4 | 0.001 | 0.028 | HS_5_vs_HS_0 |
| ALPK3 | 516866 | alpha kinase 3 | -0.9 | 0.001 | 0.028 | HS_5_vs_HS_0 |
| TMEM187 | 508380 | transmembrane protein 187 | -0.5 | 0.001 | 0.028 | HS_5_vs_HS_0 |
| ZNF484 | 538489 | None | 0.6 | 0.001 | 0.028 | HS_5_vs_HS_0 |
| TMPRSS2 | 511037 | None | -1.0 | 0.001 | 0.028 | HS_5_vs_HS_0 |
| SLC50A1 | 520463 | solute carrier family 50 member 1 | -0.3 | 0.001 | 0.028 | HS_5_vs_HS_0 |
| MRPL41 | 506521 | mitochondrial ribosomal protein L41 | -0.5 | 0.001 | 0.028 | HS_5_vs_HS_0 |
| LOC112442855 | 112442855 | U2 spliceosomal RNA | -2.1 | 0.001 | 0.028 | HS_5_vs_HS_0 |
| B3GALT4 | 768038 | beta-1,3-galactosyltransferase 4 | -0.6 | 0.001 | 0.028 | HS_5_vs_HS_0 |
| GIMAP4 | 510751 | GTPase, IMAP family member 4 | 0.4 | 0.001 | 0.028 | HS_5_vs_HS_0 |
| LOC112443328 | 112443328 | Small nucleolar RNA SNORA3/SNORA45 family | 0.7 | 0.001 | 0.028 | HS_5_vs_HS_0 |
| RASA1 | 282032 | RAS p21 protein activator 1 | 0.3 | 0.001 | 0.028 | HS_5_vs_HS_0 |
| KIF18A | 537566 | kinesin family member 18A | 0.7 | 0.001 | 0.028 | HS_5_vs_HS_0 |
| LMF1 | 505124 | lipase maturation factor 1 | -0.4 | 0.001 | 0.028 | HS_5_vs_HS_0 |
| METTL21A | 615773 | None | -0.3 | 0.001 | 0.028 | HS_5_vs_HS_0 |
| ADAT3 | 132342112 | None | -0.3 | 0.001 | 0.028 | HS_5_vs_HS_0 |
| LOC112442265 | 112442265 | None | -0.8 | 0.001 | 0.028 | HS_5_vs_HS_0 |
| MRM1 | 526282 | None | -0.4 | 0.001 | 0.028 | HS_5_vs_HS_0 |
| FBXO33 | 539998 | F-box protein 33 | -1.2 | 0.001 | 0.028 | HS_5_vs_HS_0 |
| STAT4 | 515988 | signal transducer and activator of transcription 4 | 0.5 | 0.001 | 0.028 | HS_5_vs_HS_0 |
| BCDIN3D | 505650 | None | 0.6 | 0.001 | 0.028 | HS_5_vs_HS_0 |
| CSNK2A1 | 282419 | casein kinase 2 alpha 1 | 0.3 | 0.001 | 0.028 | HS_5_vs_HS_0 |
| LOC132342863 | 132342863 | None | 1.1 | 0.001 | 0.028 | HS_5_vs_HS_0 |
| LOC100848895 | 100848895 | None | 0.4 | 0.001 | 0.028 | HS_5_vs_HS_0 |
| CLK1 | 613808 | CDC like kinase 1 | 0.8 | 0.001 | 0.028 | HS_5_vs_HS_0 |
| MEMO1 | 615803 | None | 0.9 | 0.001 | 0.028 | HS_5_vs_HS_0 |
| KYNU | 520327 | kynureninase | 0.6 | 0.001 | 0.029 | HS_5_vs_HS_0 |
| NFATC2 | 530401 | nuclear factor of activated T cells 2 | -0.5 | 0.001 | 0.029 | HS_5_vs_HS_0 |
| PRDM16 | 100137803 | PR/SET domain 16 | -1.1 | 0.001 | 0.029 | HS_5_vs_HS_0 |
| LOC100847159 | 100847159 | None | -1.4 | 0.001 | 0.029 | HS_5_vs_HS_0 |
| LOC104969378 | 104969378 | None | 0.6 | 0.001 | 0.029 | HS_5_vs_HS_0 |
| H2AC25 | 538911 | H2A clustered histone 25 | -0.7 | 0.001 | 0.029 | HS_5_vs_HS_0 |
| SINHCAF | 538649 | SIN3-HDAC complex associated factor | 0.6 | 0.001 | 0.029 | HS_5_vs_HS_0 |
| SLC35A1 | 536838 | solute carrier family 35 member A1 | 0.5 | 0.001 | 0.029 | HS_5_vs_HS_0 |
| NANOS1 | 786356 | nanos C2HC-type zinc finger 1 | 0.7 | 0.001 | 0.029 | HS_5_vs_HS_0 |
| SYT5 | 539616 | synaptotagmin 5 | -1.1 | 0.001 | 0.029 | HS_5_vs_HS_0 |
| LOC112443000 | 112443000 | None | 0.6 | 0.001 | 0.029 | HS_5_vs_HS_0 |
| RBM4B | 505756 | RNA binding motif protein 4B | 0.4 | 0.001 | 0.029 | HS_5_vs_HS_0 |
| KDM4B | 508141 | lysine demethylase 4B | -0.4 | 0.001 | 0.029 | HS_5_vs_HS_0 |
| SLF1 | 520250 | SMC5-SMC6 complex localization factor 1 | -0.4 | 0.001 | 0.029 | HS_5_vs_HS_0 |
| POMC | 281416 | proopiomelanocortin | -0.5 | 0.001 | 0.029 | HS_5_vs_HS_0 |
| TRAPPC9 | 533451 | trafficking protein particle complex subunit 9 | -0.4 | 0.001 | 0.029 | HS_5_vs_HS_0 |
| LIMD2 | 508942 | None | -0.4 | 0.001 | 0.029 | HS_5_vs_HS_0 |
| IDH3G | 614145 | isocitrate dehydrogenase (NAD(+)) 3 non-catalytic subunit gamma | -0.4 | 0.001 | 0.029 | HS_5_vs_HS_0 |
| FCER1A | 506783 | Fc epsilon receptor Ia | 1.1 | 0.001 | 0.029 | HS_5_vs_HS_0 |
| VPS50 | 535248 | VPS50 subunit of EARP/GARPII complex | 0.5 | 0.001 | 0.029 | HS_5_vs_HS_0 |
| IPPK | 521083 | None | 0.7 | 0.001 | 0.029 | HS_5_vs_HS_0 |
| RFXAP | 540760 | None | 0.7 | 0.001 | 0.029 | HS_5_vs_HS_0 |
| SNX16 | 507462 | sorting nexin 16 | 0.4 | 0.001 | 0.029 | HS_5_vs_HS_0 |
| MTMR2 | 536810 | myotubularin related protein 2 | 0.5 | 0.001 | 0.029 | HS_5_vs_HS_0 |
| KCTD6 | 613649 | potassium channel tetramerization domain containing 6 | 0.3 | 0.001 | 0.029 | HS_5_vs_HS_0 |
| LOC112444184 | 112444184 | None | 1.5 | 0.001 | 0.029 | HS_5_vs_HS_0 |
| ABAT | 280969 | None | 1.1 | 0.001 | 0.029 | HS_5_vs_HS_0 |
| CCT6A | 521540 | chaperonin containing TCP1 subunit 6A | 0.6 | 0.001 | 0.029 | HS_5_vs_HS_0 |
| ST6GALNAC4 | 404124 | ST6 N-acetylgalactosaminide alpha-2,6-sialyltransferase 4 | -0.5 | 0.001 | 0.029 | HS_5_vs_HS_0 |
| TP53 | 281542 | tumor protein p53 | -0.3 | 0.001 | 0.029 | HS_5_vs_HS_0 |
| LOC112442846 | 112442846 | U2 spliceosomal RNA | -1.7 | 0.001 | 0.029 | HS_5_vs_HS_0 |
| ORC5 | 519409 | origin recognition complex subunit 5 | 0.6 | 0.001 | 0.029 | HS_5_vs_HS_0 |
| SIMC1 | 100336733 | None | 0.6 | 0.001 | 0.029 | HS_5_vs_HS_0 |
| LOC107133209 | 107133209 | None | -0.9 | 0.001 | 0.029 | HS_5_vs_HS_0 |
| LOC100847190 | 100847190 | None | -0.4 | 0.001 | 0.029 | HS_5_vs_HS_0 |
| SF3B2 | 531931 | splicing factor 3b subunit 2 | -0.4 | 0.001 | 0.029 | HS_5_vs_HS_0 |
| TARDBP | 540632 | TAR DNA binding protein | 0.4 | 0.001 | 0.029 | HS_5_vs_HS_0 |
| ZNF461 | 100296502 | zinc finger protein 461 | 0.7 | 0.001 | 0.029 | HS_5_vs_HS_0 |
| EPPK1 | 100337278 | None | -0.7 | 0.001 | 0.029 | HS_5_vs_HS_0 |
| LOC132342869 | 132342869 | None | -1.1 | 0.001 | 0.029 | HS_5_vs_HS_0 |
| C16H1orf74 | 511599 | chromosome 16 C1orf74 homolog | 0.7 | 0.001 | 0.029 | HS_5_vs_HS_0 |
| ASXL1 | 522091 | None | -0.5 | 0.001 | 0.029 | HS_5_vs_HS_0 |
| LOC619000 | 619000 | None | 0.6 | 0.001 | 0.030 | HS_5_vs_HS_0 |
| FANCM | 513626 | FA complementation group M | 0.4 | 0.001 | 0.030 | HS_5_vs_HS_0 |
| LOC515042 | 515042 | None | -0.3 | 0.001 | 0.030 | HS_5_vs_HS_0 |
| HROB | 615299 | homologous recombination factor with OB-fold | -0.9 | 0.001 | 0.030 | HS_5_vs_HS_0 |
| ARL8A | 101906089 | ADP ribosylation factor like GTPase 8A | -0.6 | 0.001 | 0.030 | HS_5_vs_HS_0 |
| SMG6 | 516411 | None | -0.4 | 0.001 | 0.030 | HS_5_vs_HS_0 |
| MIR2887-1 | 100498812 | bta-mir-2887-1 | -2.4 | 0.001 | 0.030 | HS_5_vs_HS_0 |
| AREL1 | 539472 | apoptosis resistant E3 ubiquitin protein ligase 1 | -0.4 | 0.001 | 0.030 | HS_5_vs_HS_0 |
| MAPT | 281296 | microtubule associated protein tau | -1.0 | 0.001 | 0.030 | HS_5_vs_HS_0 |
| SPTBN2 | 132344210 | spectrin beta, non-erythrocytic 2 | -0.6 | 0.001 | 0.030 | HS_5_vs_HS_0 |
| KCNAB2 | 541597 | None | -0.4 | 0.001 | 0.030 | HS_5_vs_HS_0 |
| GPR137B | 613826 | None | 0.5 | 0.001 | 0.030 | HS_5_vs_HS_0 |
| TMEM19 | 538974 | transmembrane protein 19 | 0.6 | 0.001 | 0.031 | HS_5_vs_HS_0 |
| LOC101907622 | 101907622 | None | 0.8 | 0.001 | 0.031 | HS_5_vs_HS_0 |
| MPST | 507313 | mercaptopyruvate sulfurtransferase | -0.3 | 0.001 | 0.031 | HS_5_vs_HS_0 |
| H2AJ | 618489 | H2A.J histone | -0.5 | 0.001 | 0.031 | HS_5_vs_HS_0 |
| PRKN | 530858 | parkin RBR E3 ubiquitin protein ligase | -0.8 | 0.001 | 0.031 | HS_5_vs_HS_0 |
| DCP2 | 539770 | decapping mRNA 2 | -0.7 | 0.001 | 0.031 | HS_5_vs_HS_0 |
| H2BC11 | 522960 | H2B clustered histone 11 | -0.8 | 0.001 | 0.031 | HS_5_vs_HS_0 |
| TTYH3 | 512271 | tweety family member 3 | -0.7 | 0.001 | 0.031 | HS_5_vs_HS_0 |
| SCIN | 281478 | None | 0.8 | 0.001 | 0.031 | HS_5_vs_HS_0 |
| ZNF93 | 510078 | None | 0.5 | 0.001 | 0.031 | HS_5_vs_HS_0 |
| LOC132344119 | 132344119 | None | 1.2 | 0.001 | 0.031 | HS_5_vs_HS_0 |
| ADGRA1 | 518385 | None | -1.0 | 0.001 | 0.031 | HS_5_vs_HS_0 |
| LOC132342615 | 132342615 | None | -0.7 | 0.001 | 0.031 | HS_5_vs_HS_0 |
| LOC100848642 | 100848642 | None | -0.7 | 0.001 | 0.031 | HS_5_vs_HS_0 |
| MRPL22 | 614639 | mitochondrial ribosomal protein L22 | 0.3 | 0.001 | 0.031 | HS_5_vs_HS_0 |
| C11H2orf49 | 540024 | None | 0.4 | 0.001 | 0.031 | HS_5_vs_HS_0 |
| SOCS5 | 514773 | suppressor of cytokine signaling 5 | 0.4 | 0.001 | 0.031 | HS_5_vs_HS_0 |
| ZNF467 | 527301 | zinc finger protein 467 | -0.4 | 0.001 | 0.031 | HS_5_vs_HS_0 |
| PDE9A | 511665 | None | -0.8 | 0.001 | 0.031 | HS_5_vs_HS_0 |
| TAF13 | 513065 | TATA-box binding protein associated factor 13 | 0.7 | 0.001 | 0.031 | HS_5_vs_HS_0 |
| TMEM165 | 532600 | transmembrane protein 165 | 0.4 | 0.001 | 0.031 | HS_5_vs_HS_0 |
| OTUB2 | 504880 | OTU deubiquitinase, ubiquitin aldehyde binding 2 | 1.0 | 0.001 | 0.031 | HS_5_vs_HS_0 |
| LOC112447196 | 112447196 | U1 spliceosomal RNA | -1.7 | 0.001 | 0.031 | HS_5_vs_HS_0 |
| GPX3 | 281210 | None | -1.0 | 0.001 | 0.031 | HS_5_vs_HS_0 |
| LOC616782 | 616782 | None | -1.0 | 0.001 | 0.031 | HS_5_vs_HS_0 |
| ZWILCH | 534811 | zwilch kinetochore protein | 0.4 | 0.001 | 0.031 | HS_5_vs_HS_0 |
| SLC19A2 | 532860 | solute carrier family 19 member 2 | 0.7 | 0.001 | 0.031 | HS_5_vs_HS_0 |
| JUP | 445543 | junction plakoglobin | -0.7 | 0.001 | 0.031 | HS_5_vs_HS_0 |
| HORMAD1 | 529615 | HORMA domain containing 1 | 1.1 | 0.001 | 0.031 | HS_5_vs_HS_0 |
| GON7 | 505131 | GON7 subunit of KEOPS complex | 0.4 | 0.001 | 0.031 | HS_5_vs_HS_0 |
| FBXO6 | 513023 | F-box protein 6 | -0.4 | 0.001 | 0.032 | HS_5_vs_HS_0 |
| OBI1 | 782050 | ORC ubiquitin ligase 1 | 0.8 | 0.001 | 0.032 | HS_5_vs_HS_0 |
| LOC787397 | 787397 | None | 0.7 | 0.001 | 0.032 | HS_5_vs_HS_0 |
| LOC101906226 | 101906226 | None | -0.3 | 0.001 | 0.032 | HS_5_vs_HS_0 |
| LOC107133207 | 107133207 | None | -0.7 | 0.001 | 0.032 | HS_5_vs_HS_0 |
| ZNF892 | 132342086 | zinc finger protein 892 | 0.7 | 0.001 | 0.032 | HS_5_vs_HS_0 |
| STXBP6 | 534718 | None | 1.2 | 0.001 | 0.032 | HS_5_vs_HS_0 |
| RGS9 | 281453 | None | -0.5 | 0.001 | 0.032 | HS_5_vs_HS_0 |
| TAF3 | 506674 | TATA-box binding protein associated factor 3 | -0.6 | 0.001 | 0.033 | HS_5_vs_HS_0 |
| PRPF40B | 513782 | pre-mRNA processing factor 40 homolog B | -0.5 | 0.001 | 0.033 | HS_5_vs_HS_0 |
| ARAP1 | 511889 | ArfGAP with RhoGAP domain, ankyrin repeat and PH domain 1 | -0.4 | 0.001 | 0.033 | HS_5_vs_HS_0 |
| KLHL22 | 540134 | kelch like family member 22 | -0.4 | 0.001 | 0.033 | HS_5_vs_HS_0 |
| RBM10 | 505749 | RNA binding motif protein 10 | -0.5 | 0.001 | 0.033 | HS_5_vs_HS_0 |
| C3H1orf109 | 508021 | AFG2 interacting ribosome maturation factor | 0.5 | 0.001 | 0.033 | HS_5_vs_HS_0 |
| HACD4 | 618814 | 3-hydroxyacyl-CoA dehydratase 4 | 0.6 | 0.001 | 0.033 | HS_5_vs_HS_0 |
| AAGAB | 507035 | alpha and gamma adaptin binding protein | 0.5 | 0.001 | 0.033 | HS_5_vs_HS_0 |
| ZNF770 | 786537 | None | 0.6 | 0.001 | 0.033 | HS_5_vs_HS_0 |
| HSPA14 | 534751 | heat shock protein family A (Hsp70) member 14 | 0.5 | 0.001 | 0.033 | HS_5_vs_HS_0 |
| SBNO1 | 540582 | strawberry notch homolog 1 | 0.3 | 0.001 | 0.033 | HS_5_vs_HS_0 |
| LOC100848357 | 100848357 | None | 0.5 | 0.001 | 0.033 | HS_5_vs_HS_0 |
| ERI1 | 540728 | exoribonuclease 1 | 0.6 | 0.001 | 0.033 | HS_5_vs_HS_0 |
| FAM89A | 616421 | family with sequence similarity 89 member A | 0.7 | 0.001 | 0.033 | HS_5_vs_HS_0 |
| CPM | 513281 | carboxypeptidase M | 0.8 | 0.001 | 0.033 | HS_5_vs_HS_0 |
| WDR89 | 539045 | WD repeat domain 89 | 0.7 | 0.001 | 0.033 | HS_5_vs_HS_0 |
| AHSA1 | 539220 | activator of HSP90 ATPase activity 1 | 0.5 | 0.001 | 0.033 | HS_5_vs_HS_0 |
| LOC788724 | 788724 | None | -1.3 | 0.001 | 0.033 | HS_5_vs_HS_0 |
| LOC786796 | 786796 | None | -0.9 | 0.001 | 0.033 | HS_5_vs_HS_0 |
| ZYX | 768226 | zyxin | -0.9 | 0.001 | 0.033 | HS_5_vs_HS_0 |
| NSD2 | 540769 | nuclear receptor binding SET domain protein 2 | -0.5 | 0.001 | 0.033 | HS_5_vs_HS_0 |
| DIP2C | 535136 | disco interacting C | -0.5 | 0.001 | 0.033 | HS_5_vs_HS_0 |
| LOC101905779 | 101905779 | None | -0.7 | 0.001 | 0.033 | HS_5_vs_HS_0 |
| TSPAN15 | 522371 | tetraspanin 15 | -0.5 | 0.001 | 0.033 | HS_5_vs_HS_0 |
| BTG3 | 541054 | BTG anti-proliferation factor 3 | 0.7 | 0.001 | 0.033 | HS_5_vs_HS_0 |
| CYP51A1 | 505060 | cytochrome P450 family 51 subfamily A member 1 | 0.5 | 0.001 | 0.033 | HS_5_vs_HS_0 |
| SLC41A2 | 524417 | solute carrier family 41 member 2 | 0.6 | 0.001 | 0.033 | HS_5_vs_HS_0 |
| LOC132346013 | 132346013 | None | 1.9 | 0.001 | 0.033 | HS_5_vs_HS_0 |
| LOC100140915 | 100140915 | None | 0.7 | 0.001 | 0.033 | HS_5_vs_HS_0 |
| TMEM100 | 613987 | transmembrane protein 100 | 1.1 | 0.001 | 0.033 | HS_5_vs_HS_0 |
| IL17RE | 783335 | interleukin 17 receptor E | 0.9 | 0.001 | 0.033 | HS_5_vs_HS_0 |
| SOCS1 | 518795 | suppressor of cytokine signaling 1 | 1.1 | 0.001 | 0.033 | HS_5_vs_HS_0 |
| SLC25A32 | 782162 | solute carrier family 25 member 32 | 0.4 | 0.001 | 0.033 | HS_5_vs_HS_0 |
| STAP1 | 507296 | signal transducing adaptor family member 1 | 0.5 | 0.001 | 0.033 | HS_5_vs_HS_0 |
| CISD2 | 781260 | None | 0.5 | 0.001 | 0.033 | HS_5_vs_HS_0 |
| MAK16 | 504683 | MAK16 homolog | 0.5 | 0.001 | 0.033 | HS_5_vs_HS_0 |
| TANC1 | 507983 | tetratricopeptide repeat, ankyrin repeat and coiled-coil containing 1 | -0.4 | 0.002 | 0.033 | HS_5_vs_HS_0 |
| COPS7A | 505079 | None | -0.4 | 0.001 | 0.033 | HS_5_vs_HS_0 |
| CHD4 | 506402 | chromodomain helicase DNA binding protein 4 | -0.8 | 0.001 | 0.033 | HS_5_vs_HS_0 |
| MICALL1 | 517259 | MICAL like 1 | -0.6 | 0.001 | 0.033 | HS_5_vs_HS_0 |
| ARHGEF18 | 522521 | None | -0.6 | 0.001 | 0.033 | HS_5_vs_HS_0 |
| GANC | 530330 | None | -0.5 | 0.001 | 0.033 | HS_5_vs_HS_0 |
| LOC112448841 | 112448841 | None | -0.7 | 0.001 | 0.033 | HS_5_vs_HS_0 |
| NDUFS3 | 287327 | NADH:ubiquinone oxidoreductase core subunit S3 | -0.3 | 0.002 | 0.033 | HS_5_vs_HS_0 |
| EMP3 | 535273 | epithelial membrane protein 3 | -0.7 | 0.001 | 0.033 | HS_5_vs_HS_0 |
| C19H17orf67 | 100295656 | chromosome 19 C17orf67 homolog | -0.8 | 0.002 | 0.033 | HS_5_vs_HS_0 |
| TTLL3 | 531866 | None | -0.3 | 0.002 | 0.033 | HS_5_vs_HS_0 |
| MOSPD3 | 506163 | None | -0.4 | 0.002 | 0.033 | HS_5_vs_HS_0 |
| DNAJC4 | 533579 | None | -0.5 | 0.001 | 0.033 | HS_5_vs_HS_0 |
| BTLA | 531767 | B and T lymphocyte associated | 0.7 | 0.002 | 0.033 | HS_5_vs_HS_0 |
| SLC49A4 | 783019 | solute carrier family 49 member 4 | 0.7 | 0.001 | 0.033 | HS_5_vs_HS_0 |
| LOC132345896 | 132345896 | None | 0.7 | 0.001 | 0.033 | HS_5_vs_HS_0 |
| LOC112442563 | 112442563 | None | 0.7 | 0.002 | 0.033 | HS_5_vs_HS_0 |
| MRPL51 | 513622 | mitochondrial ribosomal protein L51 | 0.3 | 0.001 | 0.033 | HS_5_vs_HS_0 |
| ZNF189 | 540816 | zinc finger protein 189 | 0.5 | 0.001 | 0.033 | HS_5_vs_HS_0 |
| HAUS2 | 508698 | HAUS augmin like complex subunit 2 | 0.6 | 0.001 | 0.033 | HS_5_vs_HS_0 |
| PPP4R3B | 516489 | protein phosphatase 4 regulatory subunit 3B | 0.5 | 0.001 | 0.033 | HS_5_vs_HS_0 |
| RNF139 | 788471 | None | 0.5 | 0.001 | 0.033 | HS_5_vs_HS_0 |
| DEGS1 | 507290 | delta 4-desaturase, sphingolipid 1 | 0.5 | 0.001 | 0.033 | HS_5_vs_HS_0 |
| CACYBP | 618428 | calcyclin binding protein | 0.5 | 0.001 | 0.033 | HS_5_vs_HS_0 |
| ZNF565 | 538504 | zinc finger protein 565 | 0.8 | 0.001 | 0.033 | HS_5_vs_HS_0 |
| ANKRD40 | 522429 | ankyrin repeat domain 40 | 0.6 | 0.002 | 0.033 | HS_5_vs_HS_0 |
| PRORP | 532995 | protein only RNase P catalytic subunit | 0.6 | 0.001 | 0.033 | HS_5_vs_HS_0 |
| ABHD3 | 539795 | abhydrolase domain containing 3, phospholipase | 0.5 | 0.002 | 0.033 | HS_5_vs_HS_0 |
| UPK1A | 282112 | None | -0.7 | 0.002 | 0.033 | HS_5_vs_HS_0 |
| CYGB | 510299 | cytoglobin | -0.8 | 0.002 | 0.033 | HS_5_vs_HS_0 |
| SENP1 | 538091 | SUMO specific peptidase 1 | 0.4 | 0.002 | 0.033 | HS_5_vs_HS_0 |
| TNFAIP8L3 | 523131 | TNF alpha induced protein 8 like 3 | 2.1 | 0.002 | 0.033 | HS_5_vs_HS_0 |
| NANOS3 | 112447338 | None | -1.2 | 0.002 | 0.033 | HS_5_vs_HS_0 |
| RNF8 | 515933 | ring finger protein 8 | 0.5 | 0.002 | 0.034 | HS_5_vs_HS_0 |
| ZSWIM7 | 514094 | None | -0.4 | 0.002 | 0.034 | HS_5_vs_HS_0 |
| NEK9 | 534652 | None | -1.1 | 0.002 | 0.034 | HS_5_vs_HS_0 |
| PAFAH1B2 | 282514 | platelet activating factor acetylhydrolase 1b catalytic subunit 2 | 0.4 | 0.002 | 0.034 | HS_5_vs_HS_0 |
| LOC132343640 | 132343640 | None | -0.8 | 0.002 | 0.034 | HS_5_vs_HS_0 |
| HERC3 | 510924 | HECT and RLD domain containing E3 ubiquitin protein ligase 3 | -0.4 | 0.002 | 0.034 | HS_5_vs_HS_0 |
| MDH2 | 281306 | None | -0.4 | 0.002 | 0.034 | HS_5_vs_HS_0 |
| LOC101907749 | 101907749 | None | 0.7 | 0.002 | 0.034 | HS_5_vs_HS_0 |
| TRPC4AP | 532834 | transient receptor potential cation channel subfamily C member 4 associated protein | -0.3 | 0.002 | 0.034 | HS_5_vs_HS_0 |
| PAIP2 | 536619 | poly(A) binding protein interacting protein 2 | 0.3 | 0.002 | 0.034 | HS_5_vs_HS_0 |
| RINT1 | 510686 | None | 0.5 | 0.002 | 0.034 | HS_5_vs_HS_0 |
| ZNF814 | 613566 | None | 0.5 | 0.002 | 0.034 | HS_5_vs_HS_0 |
| DAZAP1 | 614783 | None | -0.4 | 0.002 | 0.034 | HS_5_vs_HS_0 |
| MKKS | 614288 | MKKS centrosomal shuttling protein | 0.5 | 0.002 | 0.034 | HS_5_vs_HS_0 |
| LOC613444 | 613444 | None | 0.5 | 0.002 | 0.034 | HS_5_vs_HS_0 |
| ABCA1 | 535379 | ATP binding cassette subfamily A member 1 | -0.8 | 0.002 | 0.034 | HS_5_vs_HS_0 |
| GSE1 | 538506 | None | -0.5 | 0.002 | 0.034 | HS_5_vs_HS_0 |
| SEPTIN5 | 615408 | septin 5 | -0.7 | 0.002 | 0.034 | HS_5_vs_HS_0 |
| FOXL3 | 783396 | None | -1.1 | 0.002 | 0.034 | HS_5_vs_HS_0 |
| NAA10 | 613636 | None | -0.4 | 0.002 | 0.034 | HS_5_vs_HS_0 |
| SCAF8 | 100298738 | SR-related CTD associated factor 8 | -0.5 | 0.002 | 0.034 | HS_5_vs_HS_0 |
| ODR4 | 507724 | odr-4 GPCR localization factor homolog | 0.3 | 0.002 | 0.034 | HS_5_vs_HS_0 |
| TMEM80 | 613612 | transmembrane protein 80 | -0.5 | 0.002 | 0.035 | HS_5_vs_HS_0 |
| C1QTNF6 | 506413 | C1q and TNF related 6 | -0.7 | 0.002 | 0.035 | HS_5_vs_HS_0 |
| PPM1N | 530233 | protein phosphatase, Mg2+/Mn2+ dependent 1N (putative) | -0.7 | 0.002 | 0.035 | HS_5_vs_HS_0 |
| C21H15orf39 | 784903 | chromosome 21 C15orf39 homolog | -0.5 | 0.002 | 0.035 | HS_5_vs_HS_0 |
| FXR1 | 536793 | FMR1 autosomal homolog 1 | 0.3 | 0.002 | 0.035 | HS_5_vs_HS_0 |
| VIM | 280955 | vimentin | -0.4 | 0.002 | 0.035 | HS_5_vs_HS_0 |
| ARL4A | 767906 | ADP ribosylation factor like GTPase 4A | 0.4 | 0.002 | 0.035 | HS_5_vs_HS_0 |
| UBA1 | 282869 | None | -0.3 | 0.002 | 0.035 | HS_5_vs_HS_0 |
| LOC132342366 | 132342366 | None | -1.0 | 0.002 | 0.035 | HS_5_vs_HS_0 |
| PLEC | 786966 | plectin | -0.6 | 0.002 | 0.035 | HS_5_vs_HS_0 |
| WDR86 | 100140069 | WD repeat domain 86 | -0.7 | 0.002 | 0.035 | HS_5_vs_HS_0 |
| CD27 | 512514 | CD27 molecule | -0.3 | 0.002 | 0.035 | HS_5_vs_HS_0 |
| LOC789029 | 789029 | None | -1.0 | 0.002 | 0.035 | HS_5_vs_HS_0 |
| ABI3 | 529835 | ABI family member 3 | -0.8 | 0.002 | 0.035 | HS_5_vs_HS_0 |
| XCR1 | 617880 | X-C motif chemokine receptor 1 | -0.8 | 0.002 | 0.035 | HS_5_vs_HS_0 |
| ARSJ | 540514 | None | 1.3 | 0.002 | 0.035 | HS_5_vs_HS_0 |
| TMED7 | 100125926 | transmembrane p24 trafficking protein 7 | 0.3 | 0.002 | 0.035 | HS_5_vs_HS_0 |
| NAA20 | 540212 | N-alpha-acetyltransferase 20, NatB catalytic subunit | 0.4 | 0.002 | 0.035 | HS_5_vs_HS_0 |
| ZNF729 | 768043 | None | 0.7 | 0.002 | 0.035 | HS_5_vs_HS_0 |
| B4GALT2 | 100125390 | beta-1,4-galactosyltransferase 2 | -0.6 | 0.002 | 0.035 | HS_5_vs_HS_0 |
| USP30 | 100140210 | ubiquitin specific peptidase 30 | -0.6 | 0.002 | 0.035 | HS_5_vs_HS_0 |
| LOC504773 | 504773 | None | -0.7 | 0.002 | 0.035 | HS_5_vs_HS_0 |
| ATP2A3 | 512313 | ATPase sarcoplasmic/endoplasmic reticulum Ca2+ transporting 3 | -0.8 | 0.002 | 0.036 | HS_5_vs_HS_0 |
| LZTS2 | 504411 | leucine zipper tumor suppressor 2 | -0.5 | 0.002 | 0.036 | HS_5_vs_HS_0 |
| LOC789996 | 789996 | None | 0.6 | 0.002 | 0.036 | HS_5_vs_HS_0 |
| SLC38A5 | 512495 | solute carrier family 38 member 5 | -0.4 | 0.002 | 0.036 | HS_5_vs_HS_0 |
| LOC504548 | 504548 | None | 0.6 | 0.002 | 0.036 | HS_5_vs_HS_0 |
| LOC789027 | 789027 | None | 0.4 | 0.002 | 0.036 | HS_5_vs_HS_0 |
| ELL | 506578 | None | -0.4 | 0.002 | 0.036 | HS_5_vs_HS_0 |
| PPCDC | 614957 | phosphopantothenoylcysteine decarboxylase | -0.4 | 0.002 | 0.036 | HS_5_vs_HS_0 |
| MBNL2 | 527679 | muscleblind like splicing regulator 2 | 0.5 | 0.002 | 0.036 | HS_5_vs_HS_0 |
| ACCS | 505649 | 1-aminocyclopropane-1-carboxylate synthase homolog (inactive) | -0.6 | 0.002 | 0.036 | HS_5_vs_HS_0 |
| IL6R | 507359 | interleukin 6 receptor | -0.3 | 0.002 | 0.036 | HS_5_vs_HS_0 |
| UBN1 | 525895 | None | -0.3 | 0.002 | 0.036 | HS_5_vs_HS_0 |
| TES | 534965 | testin LIM domain protein | 0.3 | 0.002 | 0.036 | HS_5_vs_HS_0 |
| PRNP | 281427 | prion protein | 0.6 | 0.002 | 0.036 | HS_5_vs_HS_0 |
| PRMT9 | 532021 | protein arginine methyltransferase 9 | 0.5 | 0.002 | 0.036 | HS_5_vs_HS_0 |
| ZNF473 | 784785 | None | 0.5 | 0.002 | 0.036 | HS_5_vs_HS_0 |
| CACNA1S | 100337204 | calcium voltage-gated channel subunit alpha1 S | -0.7 | 0.002 | 0.036 | HS_5_vs_HS_0 |
| SNRNP35 | 512142 | small nuclear ribonucleoprotein U11/U12 subunit 35 | -0.3 | 0.002 | 0.036 | HS_5_vs_HS_0 |
| H4C7 | 115945170 | H4 histone 16 | -0.9 | 0.002 | 0.036 | HS_5_vs_HS_0 |
| PIGQ | 508048 | None | -0.4 | 0.002 | 0.036 | HS_5_vs_HS_0 |
| DLGAP4 | 520521 | DLG associated protein 4 | -0.5 | 0.002 | 0.037 | HS_5_vs_HS_0 |
| CD82 | 506713 | CD82 molecule | -0.7 | 0.002 | 0.037 | HS_5_vs_HS_0 |
| PLEKHM1 | 523424 | pleckstrin homology and RUN domain containing M1 | -0.4 | 0.002 | 0.037 | HS_5_vs_HS_0 |
| LOC132346579 | 132346579 | None | 1.6 | 0.002 | 0.037 | HS_5_vs_HS_0 |
| HTT | 615059 | huntingtin | -0.5 | 0.002 | 0.037 | HS_5_vs_HS_0 |
| IAH1 | 614320 | None | -0.4 | 0.002 | 0.037 | HS_5_vs_HS_0 |
| AGRN | 525795 | agrin | -1.3 | 0.002 | 0.037 | HS_5_vs_HS_0 |
| KLF8 | 525558 | KLF transcription factor 8 | -0.6 | 0.002 | 0.037 | HS_5_vs_HS_0 |
| HMGCS1 | 407767 | 3-hydroxy-3-methylglutaryl-CoA synthase 1 | 0.5 | 0.002 | 0.037 | HS_5_vs_HS_0 |
| TAF8 | 539938 | TATA-box binding protein associated factor 8 | 0.5 | 0.002 | 0.037 | HS_5_vs_HS_0 |
| MSMO1 | 504481 | methylsterol monooxygenase 1 | 0.6 | 0.002 | 0.037 | HS_5_vs_HS_0 |
| TMEM170B | 101906104 | transmembrane protein 170B | 0.4 | 0.002 | 0.037 | HS_5_vs_HS_0 |
| FANCD2OS | 768064 | FANCD2 opposite strand | -0.8 | 0.002 | 0.037 | HS_5_vs_HS_0 |
| POT1 | 511292 | protection of telomeres 1 | 0.5 | 0.002 | 0.037 | HS_5_vs_HS_0 |
| MAD1L1 | 517837 | None | -0.4 | 0.002 | 0.037 | HS_5_vs_HS_0 |
| OMG | 407186 | oligodendrocyte myelin glycoprotein | -0.6 | 0.002 | 0.037 | HS_5_vs_HS_0 |
| RORC | 527470 | RAR related orphan receptor C | -0.7 | 0.002 | 0.037 | HS_5_vs_HS_0 |
| GTF2F2 | 509259 | ral transcription factor IIF subunit 2 | 0.4 | 0.002 | 0.037 | HS_5_vs_HS_0 |
| RPP14 | 515208 | hydroxyacyl-thioester dehydratase type 2 | 0.4 | 0.002 | 0.037 | HS_5_vs_HS_0 |
| PDHA1 | 407109 | pyruvate dehydrogenase E1 subunit alpha 1 | 0.6 | 0.002 | 0.037 | HS_5_vs_HS_0 |
| SDK1 | 516333 | None | -0.7 | 0.002 | 0.038 | HS_5_vs_HS_0 |
| MTF2 | 615211 | metal response element binding transcription factor 2 | 0.4 | 0.002 | 0.038 | HS_5_vs_HS_0 |
| SNX2 | 509769 | sorting nexin 2 | 0.5 | 0.002 | 0.038 | HS_5_vs_HS_0 |
| HSF2 | 510557 | heat shock transcription factor 2 | 0.6 | 0.002 | 0.038 | HS_5_vs_HS_0 |
| RARS2 | 525894 | arginyl-tRNA synthetase 2, mitochondrial | 0.3 | 0.002 | 0.038 | HS_5_vs_HS_0 |
| LOC104972363 | 104972363 | None | -0.7 | 0.002 | 0.038 | HS_5_vs_HS_0 |
| LOC101902301 | 101902301 | None | 0.6 | 0.002 | 0.038 | HS_5_vs_HS_0 |
| AHSA2 | 531017 | activator of HSP90 ATPase homolog 2 | 0.6 | 0.002 | 0.038 | HS_5_vs_HS_0 |
| WASF2 | 504482 | WASP family member 2 | -0.6 | 0.002 | 0.038 | HS_5_vs_HS_0 |
| SMARCC2 | 509060 | SWI/SNF related, matrix associated, actin dependent regulator of chromatin subfamily c member 2 | -0.4 | 0.002 | 0.038 | HS_5_vs_HS_0 |
| LOC101906086 | 101906086 | None | -0.7 | 0.002 | 0.038 | HS_5_vs_HS_0 |
| BAZ1B | 508442 | bromodomain adjacent to zinc finger domain 1B | -0.4 | 0.002 | 0.038 | HS_5_vs_HS_0 |
| UQCRC2 | 282394 | ubiquinol-cytochrome c reductase core protein 2 | 0.4 | 0.002 | 0.038 | HS_5_vs_HS_0 |
| LOC132346145 | 132346145 | None | -0.7 | 0.002 | 0.038 | HS_5_vs_HS_0 |
| ZNF394 | 522836 | zinc finger protein 394 | 0.3 | 0.002 | 0.038 | HS_5_vs_HS_0 |
| ST3GAL2 | 444879 | ST3 beta-galactoside alpha-2,3-sialyltransferase 2 | -0.4 | 0.002 | 0.038 | HS_5_vs_HS_0 |
| GRHL1 | 617248 | grainyhead like transcription factor 1 | 1.0 | 0.002 | 0.038 | HS_5_vs_HS_0 |
| PPP1R3F | 513859 | protein phosphatase 1 regulatory subunit 3F | -0.6 | 0.002 | 0.038 | HS_5_vs_HS_0 |
| CSKMT | 512401 | citrate synthase lysine methyltransferase | -0.5 | 0.002 | 0.038 | HS_5_vs_HS_0 |
| TIGD5 | 101903505 | tigger transposable element derived 5 | -0.5 | 0.002 | 0.038 | HS_5_vs_HS_0 |
| COPS4 | 540223 | COP9 signalosome subunit 4 | 0.4 | 0.002 | 0.038 | HS_5_vs_HS_0 |
| NCR3LG1 | 523303 | natural killer cell cytotoxicity receptor 3 ligand 1 | 0.7 | 0.002 | 0.038 | HS_5_vs_HS_0 |
| LOC787287 | 787287 | zinc finger protein 471 | 0.7 | 0.002 | 0.038 | HS_5_vs_HS_0 |
| CARS1 | 515715 | cysteinyl-tRNA synthetase 1 | -0.7 | 0.002 | 0.038 | HS_5_vs_HS_0 |
| ZC3H12D | 615863 | zinc finger CCCH-type containing 12D | 0.4 | 0.002 | 0.038 | HS_5_vs_HS_0 |
| GREB1 | 508204 | growth regulating estrogen receptor binding 1 | -1.0 | 0.002 | 0.038 | HS_5_vs_HS_0 |
| HM13 | 512534 | histocompatibility minor 13 | -0.4 | 0.002 | 0.038 | HS_5_vs_HS_0 |
| SHF | 512726 | Src homology 2 domain containing F | -1.0 | 0.002 | 0.039 | HS_5_vs_HS_0 |
| ZNF580 | 507619 | None | -1.0 | 0.002 | 0.039 | HS_5_vs_HS_0 |
| POLD1 | 281990 | None | -0.6 | 0.002 | 0.039 | HS_5_vs_HS_0 |
| ADSS1 | 784089 | None | -1.9 | 0.002 | 0.039 | HS_5_vs_HS_0 |
| PPP2R1A | 535321 | None | -0.5 | 0.002 | 0.039 | HS_5_vs_HS_0 |
| HNRNPA3 | 782669 | heteroous nuclear ribonucleoprotein A3 | -0.3 | 0.002 | 0.039 | HS_5_vs_HS_0 |
| PCNX3 | 536942 | pecanex 3 | -0.4 | 0.002 | 0.039 | HS_5_vs_HS_0 |
| MIOX | 508591 | None | -0.8 | 0.002 | 0.039 | HS_5_vs_HS_0 |
| MRPL10 | 515014 | mitochondrial ribosomal protein L10 | -0.4 | 0.002 | 0.039 | HS_5_vs_HS_0 |
| CTSD | 282883 | cathepsin D | -0.9 | 0.002 | 0.039 | HS_5_vs_HS_0 |
| IFITM10 | 613464 | None | -1.1 | 0.002 | 0.039 | HS_5_vs_HS_0 |
| ZZZ3 | 538498 | zinc finger ZZ-type containing 3 | 0.4 | 0.002 | 0.039 | HS_5_vs_HS_0 |
| LRP2BP | 536490 | LRP2 binding protein | -0.8 | 0.002 | 0.039 | HS_5_vs_HS_0 |
| LOC112446016 | 112446016 | None | -0.8 | 0.002 | 0.039 | HS_5_vs_HS_0 |
| WNK1 | 506433 | WNK lysine deficient protein kinase 1 | -0.4 | 0.002 | 0.039 | HS_5_vs_HS_0 |
| ADCK1 | 533372 | None | -0.4 | 0.002 | 0.039 | HS_5_vs_HS_0 |
| SFSWAP | 512326 | splicing factor SWAP | -0.3 | 0.002 | 0.039 | HS_5_vs_HS_0 |
| TIMM50 | 505489 | translocase of inner mitochondrial membrane 50 | -0.4 | 0.002 | 0.039 | HS_5_vs_HS_0 |
| U2AF2 | 507895 | U2 small nuclear RNA auxiliary factor 2 | -1.4 | 0.002 | 0.039 | HS_5_vs_HS_0 |
| QARS1 | 514586 | glutaminyl-tRNA synthetase 1 | -0.3 | 0.002 | 0.039 | HS_5_vs_HS_0 |
| CEP19 | 613916 | centrosomal protein 19 | 0.4 | 0.002 | 0.039 | HS_5_vs_HS_0 |
| PLEKHA5 | 532887 | pleckstrin homology domain containing A5 | 0.5 | 0.002 | 0.039 | HS_5_vs_HS_0 |
| NHLRC2 | 534327 | None | 0.4 | 0.002 | 0.039 | HS_5_vs_HS_0 |
| TTC9C | 786577 | tetratricopeptide repeat domain 9C | 0.6 | 0.002 | 0.039 | HS_5_vs_HS_0 |
| PIH1D1 | 509212 | None | -0.4 | 0.002 | 0.039 | HS_5_vs_HS_0 |
| HNRNPDL | 534770 | heteroous nuclear ribonucleoprotein D like | -0.3 | 0.002 | 0.039 | HS_5_vs_HS_0 |
| KANSL2 | 540194 | KAT8 regulatory NSL complex subunit 2 | 0.5 | 0.002 | 0.040 | HS_5_vs_HS_0 |
| GNL3 | 506152 | G protein nucleolar 3 | 0.5 | 0.002 | 0.040 | HS_5_vs_HS_0 |
| CRACR2B | 615055 | None | -0.7 | 0.002 | 0.040 | HS_5_vs_HS_0 |
| RAB33A | 510101 | RAB33A, member RAS onco family | -0.5 | 0.002 | 0.040 | HS_5_vs_HS_0 |
| CHMP6 | 510596 | None | -0.3 | 0.002 | 0.040 | HS_5_vs_HS_0 |
| ZNF207 | 505177 | zinc finger protein 207 | 0.3 | 0.002 | 0.040 | HS_5_vs_HS_0 |
| CLNS1A | 613968 | chloride nucleotide-sensitive channel 1A | 0.4 | 0.002 | 0.040 | HS_5_vs_HS_0 |
| SCML1 | 786670 | Scm polycomb group protein like 1 | 1.0 | 0.002 | 0.040 | HS_5_vs_HS_0 |
| RWDD3 | 614557 | None | -0.4 | 0.002 | 0.040 | HS_5_vs_HS_0 |
| LOC132345742 | 132345742 | None | 2.2 | 0.002 | 0.040 | HS_5_vs_HS_0 |
| TBC1D17 | 514858 | TBC1 domain family member 17 | -0.3 | 0.002 | 0.040 | HS_5_vs_HS_0 |
| ZNF687 | 787675 | zinc finger protein 687 | -0.7 | 0.002 | 0.040 | HS_5_vs_HS_0 |
| GPR132 | 539146 | None | -0.5 | 0.002 | 0.040 | HS_5_vs_HS_0 |
| ZNF646 | 783820 | zinc finger protein 646 | -0.6 | 0.002 | 0.040 | HS_5_vs_HS_0 |
| IFITM1 | 353510 | None | -0.7 | 0.002 | 0.040 | HS_5_vs_HS_0 |
| CCT8 | 281047 | chaperonin containing TCP1 subunit 8 | 0.6 | 0.002 | 0.040 | HS_5_vs_HS_0 |
| CFAP54 | 787705 | cilia and flagella associated protein 54 | -0.5 | 0.002 | 0.040 | HS_5_vs_HS_0 |
| LOC112441916 | 112441916 | U5 spliceosomal RNA | -1.4 | 0.002 | 0.040 | HS_5_vs_HS_0 |
| UBE2V2 | 286803 | None | 0.3 | 0.002 | 0.040 | HS_5_vs_HS_0 |
| SPIN2 | 506451 | spindlin family, member 2 | 0.7 | 0.002 | 0.040 | HS_5_vs_HS_0 |
| MYL3 | 618352 | myosin light chain 3 | -1.2 | 0.002 | 0.040 | HS_5_vs_HS_0 |
| CIPC | 506130 | CLOCK interacting pacemaker | -0.5 | 0.002 | 0.041 | HS_5_vs_HS_0 |
| NOSIP | 511234 | nitric oxide synthase interacting protein | -0.4 | 0.002 | 0.041 | HS_5_vs_HS_0 |
| EAF2 | 613523 | ELL associated factor 2 | 0.5 | 0.002 | 0.041 | HS_5_vs_HS_0 |
| C24H18orf25 | 615069 | ST8 alpha-N-acetyl-neuraminide alpha-2,8-sialyltransferase 5 | 0.4 | 0.002 | 0.041 | HS_5_vs_HS_0 |
| LOC132343079 | 132343079 | None | -1.0 | 0.002 | 0.041 | HS_5_vs_HS_0 |
| R3HDM4 | 614948 | R3H domain containing 4 | -0.5 | 0.002 | 0.041 | HS_5_vs_HS_0 |
| SNAI3 | 526694 | snail family transcriptional repressor 3 | -0.7 | 0.002 | 0.041 | HS_5_vs_HS_0 |
| RPE | 533764 | ribulose-5-phosphate-3-epimerase | 0.4 | 0.002 | 0.041 | HS_5_vs_HS_0 |
| ADAP2 | 534106 | None | 0.9 | 0.002 | 0.041 | HS_5_vs_HS_0 |
| ZNF341 | 523204 | zinc finger protein 341 | -0.3 | 0.002 | 0.041 | HS_5_vs_HS_0 |
| METTL26 | 514636 | methyltransferase like 26 | -0.4 | 0.002 | 0.041 | HS_5_vs_HS_0 |
| CTU1 | 522824 | cytosolic thiouridylase subunit 1 | -0.4 | 0.002 | 0.041 | HS_5_vs_HS_0 |
| LOC132342993 | 132342993 | None | -0.8 | 0.002 | 0.041 | HS_5_vs_HS_0 |
| PLEKHM3 | 533312 | pleckstrin homology domain containing M3 | 0.4 | 0.002 | 0.041 | HS_5_vs_HS_0 |
| LOC101906734 | 101906734 | None | -1.7 | 0.002 | 0.041 | HS_5_vs_HS_0 |
| LOC104969670 | 104969670 | None | 0.8 | 0.002 | 0.041 | HS_5_vs_HS_0 |
| MSH2 | 533115 | None | 0.4 | 0.002 | 0.041 | HS_5_vs_HS_0 |
| NCBP2AS2 | 783161 | NCBP2 antisense 2 (head to head) | -0.6 | 0.002 | 0.041 | HS_5_vs_HS_0 |
| SLC13A5 | 507000 | solute carrier family 13 member 5 | -0.6 | 0.002 | 0.041 | HS_5_vs_HS_0 |
| KIF22 | 506294 | None | -0.4 | 0.002 | 0.041 | HS_5_vs_HS_0 |
| DNAJA1 | 528862 | DnaJ heat shock protein family (Hsp40) member A1 | 0.9 | 0.002 | 0.041 | HS_5_vs_HS_0 |
| ARL1 | 517345 | ADP ribosylation factor like GTPase 1 | 0.5 | 0.002 | 0.041 | HS_5_vs_HS_0 |
| LOC112447080 | 112447080 | None | -0.9 | 0.002 | 0.042 | HS_5_vs_HS_0 |
| ACTG1 | 404122 | None | -0.4 | 0.002 | 0.042 | HS_5_vs_HS_0 |
| CSE1L | 518622 | chromosome segregation 1 like | 0.4 | 0.002 | 0.042 | HS_5_vs_HS_0 |
| TRAPPC4 | 785345 | trafficking protein particle complex subunit 4 | 0.3 | 0.002 | 0.042 | HS_5_vs_HS_0 |
| LOC112441500 | 112441500 | None | 0.6 | 0.002 | 0.042 | HS_5_vs_HS_0 |
| RHOT1 | 511257 | ras homolog family member T1 | 0.3 | 0.002 | 0.042 | HS_5_vs_HS_0 |
| TXLNA | 511331 | taxilin alpha | -0.9 | 0.002 | 0.042 | HS_5_vs_HS_0 |
| LOC112443660 | 112443660 | U11 spliceosomal RNA | -1.4 | 0.002 | 0.042 | HS_5_vs_HS_0 |
| UROD | 504914 | uroporphyrinogen decarboxylase | -0.3 | 0.002 | 0.042 | HS_5_vs_HS_0 |
| LOC513969 | 513969 | None | -0.6 | 0.002 | 0.042 | HS_5_vs_HS_0 |
| PEDS1 | 507694 | plasmanylethanolamine desaturase 1 | -0.4 | 0.002 | 0.042 | HS_5_vs_HS_0 |
| UBTF | 497012 | upstream binding transcription factor | -0.7 | 0.002 | 0.042 | HS_5_vs_HS_0 |
| RIN3 | 539951 | Ras and Rab interactor 3 | -1.0 | 0.002 | 0.042 | HS_5_vs_HS_0 |
| C23H6orf226 | 100140540 | None | -0.4 | 0.002 | 0.042 | HS_5_vs_HS_0 |
| SMAD7 | 535916 | SMAD family member 7 | -0.9 | 0.002 | 0.042 | HS_5_vs_HS_0 |
| TLR10 | 539791 | toll like receptor 10 | 0.5 | 0.002 | 0.042 | HS_5_vs_HS_0 |
| CTNNAL1 | 515749 | catenin alpha like 1 | 0.6 | 0.002 | 0.042 | HS_5_vs_HS_0 |
| DNAL1 | 538164 | dynein axonemal light chain 1 | 0.6 | 0.002 | 0.042 | HS_5_vs_HS_0 |
| SC5D | 525154 | None | 0.6 | 0.002 | 0.042 | HS_5_vs_HS_0 |
| AK6 | 102216273 | None | 0.3 | 0.002 | 0.042 | HS_5_vs_HS_0 |
| NUDC | 513277 | nuclear distribution C, dynein complex regulator | -0.5 | 0.002 | 0.042 | HS_5_vs_HS_0 |
| BLK | 532587 | BLK proto-onco, Src family tyrosine kinase | -0.5 | 0.002 | 0.042 | HS_5_vs_HS_0 |
| PML | 100138545 | PML nuclear body scaffold | -0.4 | 0.002 | 0.042 | HS_5_vs_HS_0 |
| SCO1 | 508586 | synthesis of cytochrome C oxidase 1 | 0.4 | 0.002 | 0.042 | HS_5_vs_HS_0 |
| RHBDD3 | 507674 | rhomboid domain containing 3 | -0.5 | 0.002 | 0.042 | HS_5_vs_HS_0 |
| SIN3A | 518504 | None | -0.5 | 0.002 | 0.042 | HS_5_vs_HS_0 |
| TMEM184C | 504966 | None | 0.7 | 0.002 | 0.042 | HS_5_vs_HS_0 |
| LOC100140121 | 100140121 | None | -1.0 | 0.002 | 0.042 | HS_5_vs_HS_0 |
| ECE1 | 281133 | endothelin converting enzyme 1 | -0.4 | 0.003 | 0.042 | HS_5_vs_HS_0 |
| MMADHC | 521533 | None | 0.4 | 0.003 | 0.042 | HS_5_vs_HS_0 |
| PBK | 534781 | PDZ binding kinase | 0.5 | 0.002 | 0.042 | HS_5_vs_HS_0 |
| EIF4G2 | 286870 | eukaryotic translation initiation factor 4 gamma 2 | 0.4 | 0.003 | 0.042 | HS_5_vs_HS_0 |
| ZKSCAN7 | 539552 | zinc finger with KRAB and SCAN domains 7 | 0.5 | 0.003 | 0.042 | HS_5_vs_HS_0 |
| LOC132345170 | 132345170 | None | -1.0 | 0.003 | 0.042 | HS_5_vs_HS_0 |
| NANP | 516539 | N-acetylneuraminic acid phosphatase | 0.6 | 0.003 | 0.042 | HS_5_vs_HS_0 |
| PVR | 526865 | None | 0.8 | 0.003 | 0.042 | HS_5_vs_HS_0 |
| POLR3K | 618296 | None | 0.3 | 0.003 | 0.042 | HS_5_vs_HS_0 |
| PALM | 786096 | paralemmin | -0.5 | 0.003 | 0.042 | HS_5_vs_HS_0 |
| TMEM250 | 784805 | None | -0.5 | 0.003 | 0.042 | HS_5_vs_HS_0 |
| DAP | 616066 | death associated protein | -0.3 | 0.003 | 0.042 | HS_5_vs_HS_0 |
| DAXX | 504336 | death domain associated protein | -0.5 | 0.003 | 0.042 | HS_5_vs_HS_0 |
| RASGRP2 | 512056 | None | -0.3 | 0.003 | 0.042 | HS_5_vs_HS_0 |
| RAB10 | 783373 | RAB10, member RAS onco family | 0.3 | 0.003 | 0.042 | HS_5_vs_HS_0 |
| ZCCHC18 | 786510 | None | 0.4 | 0.003 | 0.042 | HS_5_vs_HS_0 |
| ACAD9 | 617428 | acyl-CoA dehydrogenase family member 9 | -0.3 | 0.003 | 0.043 | HS_5_vs_HS_0 |
| KHSRP | 505564 | KH-type splicing regulatory protein | -1.0 | 0.003 | 0.043 | HS_5_vs_HS_0 |
| CHMP4B | 616164 | charged multivesicular body protein 4B | -1.1 | 0.003 | 0.043 | HS_5_vs_HS_0 |
| MOB3C | 614275 | MOB kinase activator 3C | 0.6 | 0.003 | 0.043 | HS_5_vs_HS_0 |
| ANLN | 518274 | anillin, actin binding protein | 0.5 | 0.003 | 0.043 | HS_5_vs_HS_0 |
| GOLT1A | 508464 | None | -1.0 | 0.003 | 0.043 | HS_5_vs_HS_0 |
| ATP2B1 | 282641 | ATPase plasma membrane Ca2+ transporting 1 | 0.5 | 0.003 | 0.043 | HS_5_vs_HS_0 |
| C11H9orf50 | 617008 | chromosome 11 C9orf50 homolog | -0.8 | 0.003 | 0.043 | HS_5_vs_HS_0 |
| GANAB | 540155 | glucosidase II alpha subunit | -0.5 | 0.003 | 0.043 | HS_5_vs_HS_0 |
| SNRNP48 | 513601 | small nuclear ribonucleoprotein U11/U12 subunit 48 | 0.4 | 0.003 | 0.043 | HS_5_vs_HS_0 |
| CCHCR1 | 514215 | coiled-coil alpha-helical rod protein 1 | -0.5 | 0.003 | 0.043 | HS_5_vs_HS_0 |
| ROR2 | 785924 | receptor tyrosine kinase like orphan receptor 2 | 1.2 | 0.003 | 0.043 | HS_5_vs_HS_0 |
| ZKSCAN1 | 100140434 | zinc finger with KRAB and SCAN domains 1 | -0.7 | 0.003 | 0.044 | HS_5_vs_HS_0 |
| GMFB | 615255 | glia maturation factor beta | 0.5 | 0.003 | 0.044 | HS_5_vs_HS_0 |
| ZFP30 | 100124429 | ZFP30 zinc finger protein | 0.6 | 0.003 | 0.044 | HS_5_vs_HS_0 |
| ESS2 | 513758 | None | -0.5 | 0.003 | 0.044 | HS_5_vs_HS_0 |
| SSB | 338071 | None | 0.4 | 0.003 | 0.044 | HS_5_vs_HS_0 |
| TEX14 | 522810 | testis expressed 14, intercellular bridge forming factor | -1.1 | 0.003 | 0.044 | HS_5_vs_HS_0 |
| MSANTD4 | 511226 | Myb/SANT DNA binding domain containing 4 with coiled-coils | 0.5 | 0.003 | 0.044 | HS_5_vs_HS_0 |
| HAGHL | 511836 | None | 0.4 | 0.003 | 0.044 | HS_5_vs_HS_0 |
| RWDD4 | 509865 | RWD domain containing 4 | 0.3 | 0.003 | 0.044 | HS_5_vs_HS_0 |
| ZNF33B | 520684 | zinc finger protein 33B | 0.6 | 0.003 | 0.044 | HS_5_vs_HS_0 |
| SRSF5 | 510474 | serine and arginine rich splicing factor 5 | -0.4 | 0.003 | 0.044 | HS_5_vs_HS_0 |
| RXYLT1 | 515147 | ribitol xylosyltransferase 1 | 0.6 | 0.003 | 0.044 | HS_5_vs_HS_0 |
| FDFT1 | 281767 | farnesyl-diphosphate farnesyltransferase 1 | 0.4 | 0.003 | 0.044 | HS_5_vs_HS_0 |
| KDM3B | 100848816 | lysine demethylase 3B | -0.5 | 0.003 | 0.044 | HS_5_vs_HS_0 |
| DNAL4 | 618379 | dynein axonemal light chain 4 | -0.6 | 0.003 | 0.044 | HS_5_vs_HS_0 |
| LOC112449095 | 112449095 | None | -1.0 | 0.003 | 0.044 | HS_5_vs_HS_0 |
| MCOLN2 | 532671 | mucolipin TRP cation channel 2 | 0.4 | 0.003 | 0.044 | HS_5_vs_HS_0 |
| THNSL1 | 788561 | threonine synthase like 1 | 0.5 | 0.003 | 0.044 | HS_5_vs_HS_0 |
| LINS1 | 539267 | lines homolog 1 | 0.5 | 0.003 | 0.044 | HS_5_vs_HS_0 |
| LOC112447418 | 112447418 | None | -0.5 | 0.003 | 0.044 | HS_5_vs_HS_0 |
| LGALS12 | 518931 | None | -0.3 | 0.003 | 0.044 | HS_5_vs_HS_0 |
| STK16 | 521237 | serine/threonine kinase 16 | -0.2 | 0.003 | 0.044 | HS_5_vs_HS_0 |
| GIMAP6 | 528288 | None | -0.4 | 0.003 | 0.044 | HS_5_vs_HS_0 |
| MCM5 | 506970 | minichromosome maintenance complex component 5 | -0.4 | 0.003 | 0.044 | HS_5_vs_HS_0 |
| PARP1 | 286764 | None | -0.6 | 0.003 | 0.044 | HS_5_vs_HS_0 |
| LOC101907807 | 101907807 | None | -1.0 | 0.003 | 0.044 | HS_5_vs_HS_0 |
| LOC112442867 | 112442867 | Small nucleolar RNA U3 | -1.4 | 0.003 | 0.044 | HS_5_vs_HS_0 |
| TECPR2 | 530121 | tectonin beta-propeller repeat containing 2 | -0.5 | 0.003 | 0.044 | HS_5_vs_HS_0 |
| CMTR1 | 509620 | cap methyltransferase 1 | -0.7 | 0.003 | 0.044 | HS_5_vs_HS_0 |
| XYLT1 | 541295 | xylosyltransferase 1 | -0.4 | 0.003 | 0.044 | HS_5_vs_HS_0 |
| AGFG2 | 510361 | ArfGAP with FG repeats 2 | -0.6 | 0.003 | 0.044 | HS_5_vs_HS_0 |
| POLR3F | 529410 | RNA polymerase III subunit F | 0.4 | 0.003 | 0.044 | HS_5_vs_HS_0 |
| TTLL10 | 100336825 | tubulin tyrosine ligase like 10 | 2.9 | 0.003 | 0.044 | HS_5_vs_HS_0 |
| HIP1R | 508291 | huntingtin interacting protein 1 related | -0.4 | 0.003 | 0.044 | HS_5_vs_HS_0 |
| BSCL2 | 513558 | BSCL2 lipid droplet biosis associated, seipin | -0.4 | 0.003 | 0.044 | HS_5_vs_HS_0 |
| SLC3A2 | 507107 | solute carrier family 3 member 2 | -0.4 | 0.003 | 0.044 | HS_5_vs_HS_0 |
| VMAC | 515212 | vimentin type intermediate filament associated coiled-coil protein | -0.5 | 0.003 | 0.044 | HS_5_vs_HS_0 |
| TRIM41 | 540265 | None | -0.4 | 0.003 | 0.044 | HS_5_vs_HS_0 |
| HSPB6 | 534551 | None | -0.7 | 0.003 | 0.044 | HS_5_vs_HS_0 |
| EEF1G | 326581 | None | -0.5 | 0.003 | 0.044 | HS_5_vs_HS_0 |
| TXK | 504782 | TXK tyrosine kinase | 0.4 | 0.003 | 0.044 | HS_5_vs_HS_0 |
| LOC132342570 | 132342570 | None | 0.8 | 0.003 | 0.044 | HS_5_vs_HS_0 |
| KLHL28 | 525011 | kelch like family member 28 | 0.4 | 0.003 | 0.044 | HS_5_vs_HS_0 |
| ACOT13 | 504870 | None | 0.5 | 0.003 | 0.044 | HS_5_vs_HS_0 |
| RAD23A | 540564 | RAD23 homolog A, nucleotide excision repair protein | -0.8 | 0.003 | 0.044 | HS_5_vs_HS_0 |
| DPP4 | 281122 | None | 0.5 | 0.003 | 0.045 | HS_5_vs_HS_0 |
| MRPS2 | 505681 | mitochondrial ribosomal protein S2 | -0.6 | 0.003 | 0.045 | HS_5_vs_HS_0 |
| MOB4 | 781884 | MOB family member 4, phocein | 0.4 | 0.003 | 0.045 | HS_5_vs_HS_0 |
| LOC112448903 | 112448903 | Small nucleolar RNA SNORD36 | 0.9 | 0.003 | 0.045 | HS_5_vs_HS_0 |
| LOC132342190 | 132342190 | None | 1.7 | 0.003 | 0.045 | HS_5_vs_HS_0 |
| ALAD | 510679 | aminolevulinate dehydratase | -0.5 | 0.003 | 0.045 | HS_5_vs_HS_0 |
| LOC132345128 | 132345128 | None | 1.5 | 0.003 | 0.045 | HS_5_vs_HS_0 |
| AEBP1 | 317693 | AE binding protein 1 | -0.9 | 0.003 | 0.045 | HS_5_vs_HS_0 |
| LOC132346380 | 132346380 | None | -1.0 | 0.003 | 0.045 | HS_5_vs_HS_0 |
| BRAT1 | 532550 | BRCA1 associated ATM activator 1 | -0.7 | 0.003 | 0.045 | HS_5_vs_HS_0 |
| DONSON | 522248 | None | 0.5 | 0.003 | 0.045 | HS_5_vs_HS_0 |
| FASTKD5 | 788680 | None | 0.9 | 0.003 | 0.045 | HS_5_vs_HS_0 |
| ZNF181 | 767826 | zinc finger protein 181 | 0.6 | 0.003 | 0.045 | HS_5_vs_HS_0 |
| CUPIN1 | 112441476 | cupin superfamily member 1 | 1.6 | 0.003 | 0.045 | HS_5_vs_HS_0 |
| TNK2 | 280710 | tyrosine kinase non receptor 2 | -0.5 | 0.003 | 0.045 | HS_5_vs_HS_0 |
| ACTR6 | 613596 | actin related protein 6 | 0.5 | 0.003 | 0.045 | HS_5_vs_HS_0 |
| C16H1orf159 | 509263 | chromosome 16 C1orf159 homolog | -0.3 | 0.003 | 0.045 | HS_5_vs_HS_0 |
| MID1IP1 | 615572 | MID1 interacting protein 1 | 0.4 | 0.003 | 0.045 | HS_5_vs_HS_0 |
| PPP1R3E | 527187 | None | 0.4 | 0.003 | 0.045 | HS_5_vs_HS_0 |
| MORC2 | 504634 | MORC family CW-type zinc finger 2 | -0.3 | 0.003 | 0.045 | HS_5_vs_HS_0 |
| WDR47 | 512354 | None | 0.7 | 0.003 | 0.045 | HS_5_vs_HS_0 |
| SVOPL | 518832 | ATPase H+ transporting V0 subunit a4 | 0.8 | 0.003 | 0.045 | HS_5_vs_HS_0 |
| RTCB | 525106 | RNA 2',3'-cyclic phosphate and 5'-OH ligase | 0.6 | 0.003 | 0.045 | HS_5_vs_HS_0 |
| KLRJ1 | 444861 | killer cell lectin-like receptor family J member 1 | 2.4 | 0.003 | 0.045 | HS_5_vs_HS_0 |
| LOC781726 | 781726 | None | -0.8 | 0.003 | 0.045 | HS_5_vs_HS_0 |
| MIIP | 614193 | migration and invasion inhibitory protein | -0.3 | 0.003 | 0.045 | HS_5_vs_HS_0 |
| DBP | 503577 | D-box binding PAR bZIP transcription factor | -0.6 | 0.003 | 0.045 | HS_5_vs_HS_0 |
| LOC132343948 | 132343948 | None | -1.0 | 0.003 | 0.045 | HS_5_vs_HS_0 |
| CERS6 | 616901 | ceramide synthase 6 | 0.6 | 0.003 | 0.045 | HS_5_vs_HS_0 |
| LOC112443530 | 112443530 | U2 spliceosomal RNA | -1.7 | 0.003 | 0.045 | HS_5_vs_HS_0 |
| MYO9B | 513493 | myosin IXB | -0.4 | 0.003 | 0.045 | HS_5_vs_HS_0 |
| PLIN3 | 767984 | None | -0.4 | 0.003 | 0.045 | HS_5_vs_HS_0 |
| CLN8 | 530874 | None | -0.4 | 0.003 | 0.045 | HS_5_vs_HS_0 |
| MED4 | 515299 | mediator complex subunit 4 | 0.3 | 0.003 | 0.045 | HS_5_vs_HS_0 |
| LARP1 | 505690 | None | -0.6 | 0.003 | 0.045 | HS_5_vs_HS_0 |
| LOC112446130 | 112446130 | U1 spliceosomal RNA | -1.4 | 0.003 | 0.045 | HS_5_vs_HS_0 |
| MMP28 | 519276 | matrix metallopeptidase 28 | -0.5 | 0.003 | 0.045 | HS_5_vs_HS_0 |
| PDAP1 | 100139074 | None | -0.6 | 0.003 | 0.045 | HS_5_vs_HS_0 |
| LOC112448867 | 112448867 | None | 1.3 | 0.003 | 0.045 | HS_5_vs_HS_0 |
| TMEM243 | 614280 | None | 0.3 | 0.003 | 0.045 | HS_5_vs_HS_0 |
| PTMA | 786336 | prothymosin alpha | -0.6 | 0.003 | 0.045 | HS_5_vs_HS_0 |
| NAP1L5 | 508508 | None | 0.5 | 0.003 | 0.045 | HS_5_vs_HS_0 |
| TRAF6 | 539124 | TNF receptor associated factor 6 | 0.5 | 0.003 | 0.045 | HS_5_vs_HS_0 |
| ZMIZ1 | 616740 | zinc finger MIZ-type containing 1 | -0.6 | 0.003 | 0.045 | HS_5_vs_HS_0 |
| ATP1B3 | 532844 | ATPase Na+/K+ transporting subunit beta 3 | 0.4 | 0.003 | 0.045 | HS_5_vs_HS_0 |
| LOC132345761 | 132345761 | None | -0.7 | 0.003 | 0.046 | HS_5_vs_HS_0 |
| MCM7 | 539924 | minichromosome maintenance complex component 7 | -0.3 | 0.003 | 0.046 | HS_5_vs_HS_0 |
| KBTBD8 | 530516 | None | 0.5 | 0.003 | 0.046 | HS_5_vs_HS_0 |
| LOC522845 | 522845 | None | 0.4 | 0.003 | 0.046 | HS_5_vs_HS_0 |
| PGLS | 616120 | 6-phosphogluconolactonase | -0.3 | 0.003 | 0.046 | HS_5_vs_HS_0 |
| CENPL | 615527 | centromere protein L | 0.5 | 0.003 | 0.046 | HS_5_vs_HS_0 |
| IQCE | 618833 | IQ motif containing E | -0.6 | 0.003 | 0.047 | HS_5_vs_HS_0 |
| KLC2 | 518136 | kinesin light chain 2 | -0.8 | 0.003 | 0.047 | HS_5_vs_HS_0 |
| ARHGDIA | 338054 | None | -1.2 | 0.003 | 0.047 | HS_5_vs_HS_0 |
| WDFY1 | 614729 | WD repeat and FYVE domain containing 1 | -0.5 | 0.003 | 0.047 | HS_5_vs_HS_0 |
| TPRA1 | 617772 | transmembrane protein adipocyte associated 1 | -0.8 | 0.003 | 0.047 | HS_5_vs_HS_0 |
| EHMT2 | 514062 | euchromatic histone lysine methyltransferase 2 | -0.8 | 0.003 | 0.047 | HS_5_vs_HS_0 |
| CRIPT | 617723 | CXXC repeat containing interactor of PDZ3 domain | 0.3 | 0.003 | 0.047 | HS_5_vs_HS_0 |
| NAXE | 404132 | None | -0.3 | 0.003 | 0.047 | HS_5_vs_HS_0 |
| RTN2 | 359720 | reticulon 2 | -0.7 | 0.003 | 0.047 | HS_5_vs_HS_0 |
| CALM3 | 520277 | calmodulin 3 | -0.6 | 0.003 | 0.047 | HS_5_vs_HS_0 |
| CD7 | 510073 | CD7 molecule | -0.6 | 0.003 | 0.047 | HS_5_vs_HS_0 |
| LOC132345358 | 132345358 | None | -0.7 | 0.003 | 0.047 | HS_5_vs_HS_0 |
| NR1D2 | 532076 | nuclear receptor subfamily 1 group D member 2 | -0.4 | 0.003 | 0.047 | HS_5_vs_HS_0 |
| MTFR1L | 508852 | mitochondrial fission regulator 1 like | 0.4 | 0.003 | 0.047 | HS_5_vs_HS_0 |
| CRTC2 | 540959 | CREB regulated transcription coactivator 2 | -0.4 | 0.003 | 0.047 | HS_5_vs_HS_0 |
| MBD3 | 616090 | methyl-CpG binding domain protein 3 | -1.0 | 0.003 | 0.047 | HS_5_vs_HS_0 |
| MYCBPAP | 505551 | MYCBP associated protein | -0.9 | 0.003 | 0.047 | HS_5_vs_HS_0 |
| TSC22D4 | 524543 | TSC22 domain family member 4 | -0.7 | 0.003 | 0.047 | HS_5_vs_HS_0 |
| CD99 | 509230 | CD99 molecule | -0.5 | 0.003 | 0.047 | HS_5_vs_HS_0 |
| CTH | 539159 | cystathionine gamma-lyase | 0.7 | 0.003 | 0.047 | HS_5_vs_HS_0 |
| TNFAIP1 | 539674 | TNF alpha induced protein 1 | 0.8 | 0.003 | 0.047 | HS_5_vs_HS_0 |
| GPR82 | 101904496 | G protein-coupled receptor 82 | 0.9 | 0.003 | 0.047 | HS_5_vs_HS_0 |
| EPHA1 | 525946 | EPH receptor A1 | -1.4 | 0.003 | 0.047 | HS_5_vs_HS_0 |
| DNPH1 | 613560 | None | -0.4 | 0.003 | 0.047 | HS_5_vs_HS_0 |
| SPA17 | 616974 | None | -0.6 | 0.003 | 0.047 | HS_5_vs_HS_0 |
| VPS45 | 541230 | None | 0.5 | 0.003 | 0.047 | HS_5_vs_HS_0 |
| NUDT1 | 525496 | nudix hydrolase 1 | -0.6 | 0.003 | 0.047 | HS_5_vs_HS_0 |
| RDH14 | 505949 | retinol dehydrogenase 14 | 0.3 | 0.003 | 0.047 | HS_5_vs_HS_0 |
| RIOK1 | 516289 | RIO kinase 1 | 0.4 | 0.003 | 0.047 | HS_5_vs_HS_0 |
| CEP57 | 353245 | None | 0.3 | 0.003 | 0.047 | HS_5_vs_HS_0 |
| ZNF260 | 112442192 | zinc finger protein 260 | 0.5 | 0.003 | 0.047 | HS_5_vs_HS_0 |
| LOC104976020 | 104976020 | None | 0.6 | 0.003 | 0.047 | HS_5_vs_HS_0 |
| ELMO1 | 509821 | engulfment and cell motility 1 | -0.3 | 0.003 | 0.048 | HS_5_vs_HS_0 |
| PBX2 | 100139739 | PBX homeobox 2 | -0.7 | 0.003 | 0.048 | HS_5_vs_HS_0 |
| LOC132343892 | 132343892 | None | -0.4 | 0.003 | 0.048 | HS_5_vs_HS_0 |
| LOC132342469 | 132342469 | None | 0.7 | 0.003 | 0.048 | HS_5_vs_HS_0 |
| LOC112446886 | 112446886 | U12 minor spliceosomal RNA | -1.2 | 0.003 | 0.048 | HS_5_vs_HS_0 |
| SKI | 506127 | None | -0.5 | 0.003 | 0.048 | HS_5_vs_HS_0 |
| EIF4EBP1 | 509613 | eukaryotic translation initiation factor 4E binding protein 1 | -0.8 | 0.003 | 0.048 | HS_5_vs_HS_0 |
| PRKRA | 282875 | protein activator of interferon induced protein kinase EIF2AK2 | 0.6 | 0.003 | 0.048 | HS_5_vs_HS_0 |
| ERGIC2 | 512481 | None | 0.4 | 0.003 | 0.048 | HS_5_vs_HS_0 |
| LOC132346101 | 132346101 | None | 1.8 | 0.003 | 0.048 | HS_5_vs_HS_0 |
| KCTD21 | 539645 | potassium channel tetramerization domain containing 21 | 0.5 | 0.003 | 0.048 | HS_5_vs_HS_0 |
| PRRC2B | 505073 | proline rich coiled-coil 2B | -0.5 | 0.003 | 0.048 | HS_5_vs_HS_0 |
| SCLY | 790815 | selenocysteine lyase | -0.4 | 0.003 | 0.048 | HS_5_vs_HS_0 |
| SS18L1 | 768207 | SS18L1 subunit of BAF chromatin remodeling complex | 0.5 | 0.003 | 0.048 | HS_5_vs_HS_0 |
| BCAS2 | 507944 | BCAS2 pre-mRNA processing factor | 0.3 | 0.003 | 0.048 | HS_5_vs_HS_0 |
| ZNF12 | 511194 | zinc finger protein 12 | 0.4 | 0.003 | 0.048 | HS_5_vs_HS_0 |
| NDUFB7 | 338065 | NADH:ubiquinone oxidoreductase subunit B7 | -0.5 | 0.003 | 0.048 | HS_5_vs_HS_0 |
| KLF15 | 407241 | KLF transcription factor 15 | 1.0 | 0.003 | 0.048 | HS_5_vs_HS_0 |
| HDDC2 | 509282 | None | -0.3 | 0.003 | 0.048 | HS_5_vs_HS_0 |
| LOC520336 |  |  | 0.6 | 0.003 | 0.048 | HS_5_vs_HS_0 |
| SYCE2 | 100141142 | None | -0.6 | 0.003 | 0.048 | HS_5_vs_HS_0 |
| MMP25 | 531092 | matrix metallopeptidase 25 | -0.4 | 0.003 | 0.048 | HS_5_vs_HS_0 |
| CCT4 | 613336 | chaperonin containing TCP1 subunit 4 | 0.4 | 0.003 | 0.048 | HS_5_vs_HS_0 |
| ZNF628 | 615021 | zinc finger protein 628 | -1.1 | 0.003 | 0.048 | HS_5_vs_HS_0 |
| SYS1 | 614236 | SYS1 golgi trafficking protein | -0.4 | 0.003 | 0.049 | HS_5_vs_HS_0 |
| C16H1orf53 | 112441873 | chromosome 16 C1orf53 homolog | -0.4 | 0.003 | 0.049 | HS_5_vs_HS_0 |
| SPR | 533836 | None | 0.5 | 0.003 | 0.049 | HS_5_vs_HS_0 |
| THTPA | 282090 | thiamine triphosphatase | -0.8 | 0.003 | 0.049 | HS_5_vs_HS_0 |
| THBS1 | 281530 | thrombospondin 1 | -1.0 | 0.003 | 0.049 | HS_5_vs_HS_0 |
| PRR14 | 533235 | proline rich 14 | -0.4 | 0.003 | 0.049 | HS_5_vs_HS_0 |
| BRCC3 | 519513 | BRCA1/BRCA2-containing complex subunit 3 | 0.4 | 0.003 | 0.049 | HS_5_vs_HS_0 |
| LOC786798 | 786798 | None | 0.9 | 0.003 | 0.049 | HS_5_vs_HS_0 |
| LOC282255 | 282255 | None | -1.4 | 0.003 | 0.049 | HS_5_vs_HS_0 |
| CDK7 | 515462 | cyclin dependent kinase 7 | 0.5 | 0.004 | 0.049 | HS_5_vs_HS_0 |
| LOC100299845 | 100299845 | None | -0.8 | 0.004 | 0.050 | HS_5_vs_HS_0 |
| RAB11B | 532723 | RAB11B, member RAS onco family | -0.5 | 0.004 | 0.050 | HS_5_vs_HS_0 |
| NIBAN2 | 613747 | None | -0.7 | 0.004 | 0.050 | HS_5_vs_HS_0 |
| FAM98A | 530070 | family with sequence similarity 98 member A | 0.4 | 0.004 | 0.050 | HS_5_vs_HS_0 |
| DOK7 | 524590 | None | -1.6 | 0.004 | 0.050 | HS_5_vs_HS_0 |
| LOC100139247 | 100139247 | None | -1.4 | 0.004 | 0.050 | HS_5_vs_HS_0 |
| FARS2 | 505390 | phenylalanyl-tRNA synthetase 2, mitochondrial | -0.4 | 0.004 | 0.050 | HS_5_vs_HS_0 |
| DHRS12 | 507276 | dehydrogenase/reductase 12 | -0.7 | 0.004 | 0.050 | HS_5_vs_HS_0 |
| PEF1 | 506928 | penta-EF-hand domain containing 1 | -0.4 | 0.004 | 0.050 | HS_5_vs_HS_0 |
| HADH | 532785 | hydroxyacyl-CoA dehydrogenase | -0.2 | 0.004 | 0.050 | HS_5_vs_HS_0 |
