# Supplementary material for: Transcriptome changes in leukocytes of dairy calves exposed to heat stress
Source: Transl Anim Sci. 2026 Mar 15;10:txag029. doi: 10.1093/tas/txag029 (PMC13152581; doi:10.1093/tas/txag029)
Supplement: txag029_Supplementary_Data [file txag029_supplementary_data.zip › Additional Table 10.docx]

**Additional Table 10.** Summary of the RNA-seq mapping statistics

| sample | hours_exposure | calf_id | total_sequences_raw | total_sequences_trimmed | percent_reads_survive_trimming | reads_mapped | percent_reads_mapped | total_alignments | assigned_to_genes_alignments | percent_alignments_assigned_to_genes |
| --- | --- | --- | --- | --- | --- | --- | --- | --- | --- | --- |
| tube_113 | 12 | 12 | 46871510 | 46232115 | 99 | 42167545 | 91 | 51443768 | 31145359 | 61 |
| tube_116 | 12 | 19 | 105330236 | 104658337 | 99 | 96362749 | 92 | 115999978 | 72719765 | 63 |
| tube_117 | 12 | 23 | 141178135 | 139723724 | 99 | 121494707 | 87 | 159481927 | 90982732 | 57 |
| tube_120 | 12 | 41 | 58063349 | 57527527 | 99 | 52906825 | 92 | 63709151 | 38312874 | 60 |
| tube_201 | 108 | 12 | 56013108 | 55451499 | 99 | 50086732 | 90 | 61102992 | 37285813 | 61 |
| tube_204 | 108 | 19 | 108548715 | 107837383 | 99 | 99468195 | 92 | 118892585 | 72931456 | 61 |
| tube_205 | 108 | 23 | 112268102 | 111333560 | 99 | 102786448 | 92 | 123890608 | 76944230 | 62 |
| tube_208 | 108 | 41 | 44531042 | 44160597 | 99 | 41467556 | 94 | 48413994 | 30931611 | 64 |
| tube_281 | 0 | 3 | 36492139 | 36014499 | 99 | 33315555 | 93 | 40119755 | 25781524 | 64 |
| tube_283 | 0 | 14 | 51854118 | 51212291 | 99 | 46367933 | 91 | 57405483 | 32758600 | 57 |
| tube_287 | 0 | 32 | 147500738 | 146131714 | 99 | 130769247 | 89 | 165797970 | 93078810 | 56 |
| tube_313 | 12 | 3 | 43348033 | 42880872 | 99 | 40085841 | 93 | 47340736 | 31129437 | 66 |
| tube_315 | 12 | 14 | 150504433 | 149148735 | 99 | 133582707 | 90 | 168269651 | 89233007 | 53 |
| tube_319 | 12 | 32 | 32021518 | 31528624 | 98 | 29422199 | 93 | 34626002 | 23437676 | 68 |
| tube_409 | 108 | 3 | 71846397 | 71199075 | 99 | 66650226 | 94 | 78634344 | 50583811 | 64 |
| tube_411 | 108 | 14 | 81680402 | 80884356 | 99 | 74332259 | 92 | 89744447 | 54893576 | 61 |
| tube_413 | 108 | 17 | 57373653 | 56974393 | 99 | 52795020 | 93 | 62772640 | 36752000 | 59 |
| tube_415 | 108 | 32 | 77265177 | 76440297 | 99 | 70645429 | 92 | 84634025 | 53030478 | 63 |
| tube_89 | 0 | 12 | 72745678 | 71854544 | 99 | 65289158 | 91 | 80751927 | 49244107 | 61 |
| tube_92 | 0 | 19 | 42809164 | 42321896 | 99 | 38445526 | 91 | 46872523 | 29426360 | 63 |
| tube_93 | 0 | 23 | 146250931 | 145011204 | 99 | 128671895 | 89 | 167111520 | 92868866 | 56 |
| tube_96 | 0 | 41 | 56054541 | 55427332 | 99 | 47937781 | 86 | 61877938 | 34836949 | 56 |

Additional Table 10 footnotes: Notice that the 'total_alignments' column is greater than the 'reads_mapped' column. This is because the 'total_alignments' column includes things like multimapping reads which will inflate the total number of alignments as compared to the number of reads mapped.
