# Supplementary material for: Transcriptome changes in leukocytes of dairy calves exposed to heat stress
Source: Transl Anim Sci. 2026 Mar 15;10:txag029. doi: 10.1093/tas/txag029 (PMC13152581; doi:10.1093/tas/txag029)
Supplement: txag029_Supplementary_Data [file txag029_supplementary_data.zip › Additional Table 3.docx]

**Additional Table 3**

| **feature** | **ncbi_gene_id** | **description** | **logFC** | **P.Value** | **adj.P.Val** |
| --- | --- | --- | --- | --- | --- |
| LOC101907327 | 101907327 | uncharacterized LOC101907327 | -3.01 | 5.91E-09 | 9.35E-05 |
| H2BC26 | 520120 | H2B clustered histone 26 | -2.97 | 3.81E-05 | 0.007083 |
| SPATA32 | 767871 | spermatogenesis associated 32 | -2.79 | 0.0004 | 0.019305 |
| LOC112446125 | 112446125 | U1 spliceosomal RNA | -2.49 | 0.000293 | 0.017142 |
| MIR2887-1 | 100498812 | bta-mir-2887-1 | -2.41 | 0.001163 | 0.030014 |
| LOC112446509 | 112446509 | U1 spliceosomal RNA | -2.38 | 0.000108 | 0.010616 |
| LOC112448640 | 112448640 | U5 spliceosomal RNA | -2.36 | 9.3E-06 | 0.003862 |
| LOC112442854 | 112442854 | U2 spliceosomal RNA | -2.35 | 9.08E-06 | 0.003862 |
| LOC112442847 | 112442847 | U2 spliceosomal RNA | -2.33 | 0.000154 | 0.012705 |
| LOC112449629 | 112449629 | U1 spliceosomal RNA | -2.30 | 1.64E-05 | 0.004992 |
| LOC112443327 | 112443327 | U1 spliceosomal RNA | -2.28 | 4.27E-05 | 0.007543 |
| LOC112444678 | 112444678 | Small nucleolar RNA U13 | -2.25 | 3.84E-07 | 0.000759 |
| LOC112446131 | 112446131 | U1 spliceosomal RNA | -2.21 | 0.000219 | 0.01499 |
| LOC112442845 | 112442845 | U2 spliceosomal RNA | -2.20 | 6.13E-05 | 0.00904 |
| LOC112442856 | 112442856 | U2 spliceosomal RNA | -2.19 | 0.000819 | 0.025908 |
| LOC112448940 | 112448940 | Small nucleolar RNA U13 | -2.15 | 1.02E-05 | 0.003934 |
| ZFPL1 | 613442 | zinc finger protein like 1 | -2.15 | 0.000566 | 0.022597 |
| LOC112442853 | 112442853 | U2 spliceosomal RNA | -2.15 | 0.000123 | 0.011422 |
| LOC112443614 | 112443614 | U1 spliceosomal RNA | -2.13 | 0.000157 | 0.012705 |
| LOC112442855 | 112442855 | U2 spliceosomal RNA | -2.12 | 0.000965 | 0.028005 |
| LOC112448638 | 112448638 | U5 spliceosomal RNA | -2.09 | 2.3E-06 | 0.002274 |
| H2BC7 | 505183 | H2B clustered histone 7 | -2.06 | 9.77E-06 | 0.003862 |
| LOC112442857 | 112442857 | U2 spliceosomal RNA | -2.05 | 7.05E-05 | 0.009586 |
| LOC112443159 | 112443159 | uncharacterized LOC112443159 | -2.05 | 7.34E-07 | 0.001054 |
| LOC107132278 | 107132278 | U1 spliceosomal RNA | -2.01 | 4.94E-06 | 0.003252 |
| LOC112443342 | 112443342 | U1 spliceosomal RNA | -1.98 | 5.14E-05 | 0.008378 |
| LOC112443636 | 112443636 | U1 spliceosomal RNA | -1.95 | 0.000326 | 0.017809 |
| LOC112444222 | 112444222 | U2 spliceosomal RNA | -1.95 | 0.000563 | 0.022597 |
| LOC112442843 | 112442843 | U2 spliceosomal RNA | -1.94 | 0.00021 | 0.014718 |
| ADSS1 | 784089 | adenylosuccinate synthase 1 | -1.94 | 0.002065 | 0.038893 |
| LOC112446153 | 112446153 | U5 spliceosomal RNA | -1.91 | 3.02E-05 | 0.006286 |
| LOC112442784 | 112442784 | U1 spliceosomal RNA | -1.88 | 0.000262 | 0.016343 |
| LOC112442807 | 112442807 | U1 spliceosomal RNA | -1.86 | 1.3E-05 | 0.004479 |
| LOC101910192 | 101910192 | prolactin-inducible protein homolog | -1.84 | 0.000297 | 0.017325 |
| LOC112442111 | 112442111 | U4 spliceosomal RNA | -1.84 | 5.25E-05 | 0.008378 |
| LOC132343001 | 132343001 | uncharacterized LOC132343001 | -1.80 | 9.12E-06 | 0.003862 |
| LOC112442852 | 112442852 | U2 spliceosomal RNA | -1.80 | 0.000193 | 0.013898 |
| LOC112442840 | 112442840 | small nucleolar RNA, C/D box 10 | -1.79 | 8.87E-05 | 0.009785 |
| H1-4 | 617854 | H1.4 linker histone, cluster member | -1.79 | 9.24E-05 | 0.009785 |
| H3C10 | 616819 | H3 clustered histone 10 | -1.76 | 3.99E-05 | 0.007324 |
| LOC112442846 | 112442846 | U2 spliceosomal RNA | -1.75 | 0.001097 | 0.029165 |
| LOC101906734 | 101906734 | uncharacterized LOC101906734 | -1.71 | 0.002352 | 0.041352 |
| LOC112447196 | 112447196 | U1 spliceosomal RNA | -1.68 | 0.001266 | 0.031249 |
| H2AC10 | 618824 | H2A clustered histone 10 | -1.67 | 8.97E-05 | 0.009785 |
| LOC112448627 | 112448627 | U1 spliceosomal RNA | -1.66 | 0.000758 | 0.025204 |
| LOC132343370 | 132343370 | uncharacterized LOC132343370 | -1.66 | 0.000371 | 0.018767 |
| LOC112443530 | 112443530 | U2 spliceosomal RNA | -1.66 | 0.002967 | 0.045305 |
| LOC100850875 | 100850875 | uncharacterized LOC100850875 | -1.64 | 2.04E-06 | 0.002152 |
| LOC112443671 | 112443671 | U4atac minor spliceosomal RNA | -1.63 | 5.35E-05 | 0.008378 |
| CNTNAP1 | 540997 | contactin associated protein 1 | -1.61 | 0.000156 | 0.012705 |
| H2BC18 | 615091 | H2B clustered histone 18 | -1.61 | 2.32E-07 | 0.000612 |
| H2BC8 | 787581 | H2B clustered histone 8 | -1.61 | 7.67E-05 | 0.009586 |
| MIR2291 | 100313118 | bta-mir-2291 | -1.60 | 1.91E-05 | 0.005289 |
| H2AC16 | 529277 | H2A clustered histone 16 | -1.60 | 0.000649 | 0.023727 |
| LOC112442858 | 112442858 | U2 spliceosomal RNA | -1.59 | 0.000544 | 0.022597 |
| DOK7 | 524590 | docking protein 7 | -1.59 | 0.00355 | 0.04974 |
| H2AC12 | 616634 | H2A clustered histone 12 | -1.57 | 2.75E-07 | 0.00062 |
| LOC112446710 | 112446710 | uncharacterized LOC112446710 | -1.57 | 0.000103 | 0.010343 |
| LOC112442110 | 112442110 | U4 spliceosomal RNA | -1.57 | 0.000188 | 0.013898 |
| H2AC8 | 524808 | H2A clustered histone 8 | -1.56 | 0.000164 | 0.012858 |
| H4C1 | 617905 | H4 clustered histone 1 | -1.56 | 3E-05 | 0.006286 |
| H2BC17 | 616776 | H2B clustered histone 17 | -1.54 | 7.13E-06 | 0.003633 |
| LOC132344794 | 132344794 | collagen alpha-1(I) chain-like | -1.54 | 0.000169 | 0.013096 |
| FAM131B | 617268 | family with sequence similarity 131 member B | -1.53 | 1.23E-05 | 0.004479 |
| H2BC12 | 616627 | H2B clustered histone 12 | -1.53 | 1.75E-05 | 0.005026 |
| CCDC85B | 540332 | coiled-coil domain containing 85B | -1.52 | 6.85E-05 | 0.009576 |
| H2AC17 | 104968446 | H2A clustered histone 17 | -1.52 | 1.28E-05 | 0.004479 |
| H3C12 | 616800 | H3 clustered histone 12 | -1.51 | 0.000312 | 0.01779 |
| GPR52 | 506159 | G protein-coupled receptor 52 | -1.50 | 0.000785 | 0.02553 |
| H2AC11 | 616611 | H2A clustered histone 11 | -1.49 | 1.69E-05 | 0.004992 |
| BVD1.23 | 619142 | T cell receptor delta chain variable region BVd1.23 | -1.49 | 9.12E-05 | 0.009785 |
| DNAJB13 | 520270 | DnaJ heat shock protein family (Hsp40) member B13 | -1.48 | 3.55E-05 | 0.00688 |
| JUND | 517192 | JunD proto-oncogene, AP-1 transcription factor subunit | -1.47 | 3.33E-06 | 0.002635 |
| LOC115945171 | 115945171 | H3 clustered histone 6 | -1.44 | 0.00032 | 0.017809 |
| LOC282255 | 282255 | interferon induced transmembrane protein 3 (1-8U) | -1.44 | 0.003499 | 0.049326 |
| U2AF2 | 507895 | U2 small nuclear RNA auxiliary factor 2 | -1.44 | 0.002124 | 0.039204 |
| C11H2orf16 | 100141098 | chromosome 11 C2orf16 homolog | -1.42 | 0.000229 | 0.015296 |
| LOC100139247 | 100139247 | T cell receptor alpha variable 26-1-like | -1.42 | 0.003548 | 0.04974 |
| H1-5 | 527304 | H1.5 linker histone, cluster member | -1.41 | 3.66E-05 | 0.006975 |
| LOC112449350 | 112449350 | uncharacterized LOC112449350 | -1.41 | 0.000643 | 0.023727 |
| H3C6 | 115945167 | H3 clustered histone 6 | -1.40 | 0.000162 | 0.012854 |
| LOC100847159 | 100847159 | uncharacterized LOC100847159 | -1.40 | 0.001031 | 0.028795 |
| LOC112441916 | 112441916 | U5 spliceosomal RNA | -1.40 | 0.002235 | 0.04016 |
| LOC101907887 | 101907887 | uncharacterized LOC101907887 | -1.38 | 6.41E-06 | 0.003433 |
| BAIAP3 | 132342081 | BAI1 associated protein 3 | -1.37 | 5.29E-05 | 0.008378 |
| H2AC13 | 783767 | H2A clustered histone 13 | -1.37 | 0.000386 | 0.018929 |
| LOC112446130 | 112446130 | U1 spliceosomal RNA | -1.36 | 0.002999 | 0.045397 |
| LOC112443660 | 112443660 | U11 spliceosomal RNA | -1.36 | 0.002444 | 0.041846 |
| EPHA1 | 525946 | EPH receptor A1 | -1.36 | 0.003214 | 0.047075 |
| LOC112442867 | 112442867 | Small nucleolar RNA U3 | -1.36 | 0.002774 | 0.044198 |
| LOC619094 | 619094 | histone H4-like | -1.35 | 0.000734 | 0.024941 |
| H2AC7 | 104975683 | H2A clustered histone 7 | -1.35 | 1.22E-05 | 0.004479 |
| ASIC1 | 538244 | acid sensing ion channel subunit 1 | -1.35 | 1.27E-06 | 0.001545 |
| LOC505306 | 505306 | T cell receptor delta variable 1 | -1.34 | 0.000422 | 0.020109 |
| LOC100847791 | 100847791 | uncharacterized LOC100847791 | -1.34 | 2.77E-07 | 0.004378 |
| C17H5orf52 | 787653 | chromosome 17 C5orf52 homolog | -1.33 | 1.68E-07 | 0.000575 |
| STUB1 | 504565 | STIP1 homology and U-box containing protein 1 | -1.32 | 0.000401 | 0.019305 |
| H2BC6 | 787465 | H2B clustered histone 6 | -1.31 | 9.49E-08 | 0.000575 |
| LOC104976279 | 104976279 | uncharacterized LOC104976279 | -1.30 | 0.000249 | 0.01586 |
| LOC788724 | 788724 | histone H2A type 2-A | -1.30 | 0.001402 | 0.032897 |
| AGRN | 525795 | agrin | -1.27 | 0.001851 | 0.036665 |
| LOC132345979 | 132345979 | uncharacterized LOC132345979 | -1.27 | 8.99E-05 | 0.009785 |
| H3C8 | 115945169 | H3 clustered histone 8 | -1.27 | 0.000281 | 0.016662 |
| H1-0 | 617975 | H1.0 linker histone | -1.26 | 7.38E-05 | 0.009586 |
| H4C8 | 527388 | H4 clustered histone 8 | -1.26 | 0.000103 | 0.010343 |
| LOC100140403 | 100140403 | uncharacterized LOC100140403 | -1.26 | 0.000698 | 0.024489 |
| H3C1 | 517139 | H3 clustered histone 1 | -1.26 | 0.000131 | 0.011906 |
| MAP1A | 515593 | microtubule associated protein 1A | -1.26 | 3.51E-06 | 0.002639 |
| PAQR6 | 516498 | progestin and adipoQ receptor family member 6 | -1.25 | 1.17E-06 | 0.001539 |
| H2AC15 | 614970 | H2A clustered histone 15 | -1.24 | 9.17E-05 | 0.009785 |
| H1-12 | 515957 | H1.12 linker histone, cluster member | -1.24 | 2.63E-05 | 0.005943 |
| H1-2 | 513971 | H1.2 linker histone, cluster member | -1.23 | 1.46E-07 | 0.000575 |
| LOC101904090 | 101904090 | uncharacterized LOC101904090 | -1.23 | 0.000594 | 0.022771 |
| LOC132346328 | 132346328 | uncharacterized LOC132346328 | -1.22 | 0.000675 | 0.024428 |
| CYSRT1 | 618457 | cysteine rich tail 1 | -1.22 | 0.000955 | 0.027954 |
| CPNE5 | 508482 | copine 5 | -1.22 | 0.000149 | 0.012596 |
| ZNF512B | 614014 | zinc finger protein 512B | -1.22 | 2.91E-05 | 0.006286 |
| H1-3 | 509275 | H1.3 linker histone, cluster member | -1.22 | 0.000245 | 0.01586 |
| H2BC3 | 525512 | H2B clustered histone 3 | -1.21 | 2.39E-05 | 0.005635 |
| LOC132344334 | 132344334 | uncharacterized LOC132344334 | -1.21 | 3.02E-05 | 0.006286 |
| H4C6 | 115945168 | H4 clustered histone 6 | -1.21 | 0.00079 | 0.025542 |
| C17H5orf52 | 787653 | chromosome 17 C5orf52 homolog | -1.21 | 9.1E-06 | 0.043531 |
| LOC112446886 | 112446886 | U12 minor spliceosomal RNA | -1.21 | 0.003296 | 0.04765 |
| SNX11 | 534567 | sorting nexin 11 | -1.20 | 0.000727 | 0.02488 |
| H2AC4 | 104975684 | H2A clustered histone 4 | -1.20 | 0.000524 | 0.022152 |
| ARHGDIA | 338054 | Rho GDP dissociation inhibitor alpha | -1.20 | 0.003127 | 0.046663 |
| CIC | 538483 | capicua transcriptional repressor | -1.19 | 8.47E-05 | 0.009724 |
| LOC115945165 | 115945165 | H3 clustered histone 6 | -1.18 | 0.000158 | 0.012705 |
| LOC112449404 | 112449404 | small nucleolar RNA, C/D box 12C | -1.18 | 2.69E-05 | 0.005994 |
| LOC132346654 | 132346654 | uncharacterized LOC132346654 | -1.18 | 1.59E-05 | 0.004992 |
| MYL3 | 618352 | myosin light chain 3 | -1.18 | 0.002252 | 0.040344 |
| LOC112441904 | 112441904 | Small nucleolar RNA SNORD78 | -1.17 | 0.000154 | 0.012705 |
| PLXND1 | 781625 | plexin D1 | -1.17 | 0.000569 | 0.022597 |
| SOX4 | 768313 | SRY-box transcription factor 4 | -1.17 | 7.54E-05 | 0.009586 |
| FBXO36 | 617339 | F-box protein 36 | -1.16 | 0.000688 | 0.024487 |
| FBXO33 | 539998 | F-box protein 33 | -1.16 | 0.001006 | 0.02844 |
| H4C3 | 115945166 | H4 clustered histone 3 | -1.16 | 0.000114 | 0.010753 |
| NANOS3 | 112447338 | nanos C2HC-type zinc finger 3 | -1.15 | 0.001532 | 0.03345 |
| SYT5 | 539616 | synaptotagmin 5 | -1.14 | 0.001055 | 0.028984 |
| BREH1 | 497207 | retinyl ester hydrolase type 1 | -1.14 | 0.000322 | 0.017809 |
| IFITM10 | 613464 | interferon induced transmembrane protein 10 | -1.14 | 0.002083 | 0.039013 |
| LOC781785 | 781785 | T-cell receptor alpha chain V region 2B4-like | -1.14 | 4.67E-06 | 0.003207 |
| LOC132342869 | 132342869 | uncharacterized LOC132342869 | -1.13 | 0.001117 | 0.029214 |
| LOC107131750 | 107131750 | H3 clustered histone 6 | -1.13 | 0.000366 | 0.018767 |
| FOXL3 | 783396 | forkhead box L3 | -1.12 | 0.001614 | 0.034283 |
| H4C12 | 115945172 | H4 clustered histone 12 | -1.12 | 0.000556 | 0.022597 |
| G6PD | 281179 | glucose-6-phosphate dehydrogenase | -1.12 | 0.000682 | 0.024458 |
| ATN1 | 513125 | atrophin 1 | -1.12 | 8.05E-05 | 0.009646 |
| NEK9 | 534652 | NIMA related kinase 9 | -1.12 | 0.001551 | 0.033712 |
| LOC112444557 | 112444557 | small Cajal body-specific RNA 18 | -1.11 | 2.23E-05 | 0.005417 |
| CCER2 | 614600 | coiled-coil glutamate rich protein 2 | -1.11 | 8.36E-06 | 0.003862 |
| NME4 | 789324 | NME/NM23 nucleoside diphosphate kinase 4 | -1.10 | 0.000141 | 0.012178 |
| PRSS50 | 518845 | serine protease 50 | -1.10 | 0.000459 | 0.020708 |
| CHMP4B | 616164 | charged multivesicular body protein 4B | -1.09 | 0.002574 | 0.042788 |
| TEX14 | 522810 | testis expressed 14, intercellular bridge forming factor | -1.08 | 0.002674 | 0.043586 |
| LOC112449037 | 112449037 | uncharacterized LOC112449037 | -1.08 | 0.000161 | 0.012849 |
| ZNF628 | 615021 | zinc finger protein 628 | -1.08 | 0.003407 | 0.048415 |
| PRDM16 | 100137803 | PR/SET domain 16 | -1.07 | 0.001032 | 0.028795 |
| CSTPP1 | 505437 | centriolar satellite-associated tubulin polyglutamylase complex regulator 1 | -1.07 | 0.000228 | 0.015293 |
| ALAS2 | 511791 | 5'-aminolevulinate synthase 2 | -1.06 | 0.000424 | 0.020122 |
| ADIRF | 613941 | adipogenesis regulatory factor | -1.05 | 0.000192 | 0.013898 |
| TNXB | 282654 | tenascin XB | -1.05 | 0.000372 | 0.018767 |
| GOLT1A | 508464 | golgi transport 1A | -1.05 | 0.002604 | 0.043142 |
| JAZF1 | 616701 | JAZF zinc finger 1 | -1.05 | 7.59E-05 | 0.009586 |
| LOC132343079 | 132343079 | uncharacterized LOC132343079 | -1.05 | 0.002289 | 0.04078 |
| ACAP3 | 515444 | ArfGAP with coiled-coil, ankyrin repeat and PH domains 3 | -1.05 | 0.000411 | 0.019669 |
| GPX3 | 281210 | glutathione peroxidase 3 | -1.05 | 0.001273 | 0.031343 |
| EXD3 | 618437 | exonuclease 3'-5' domain containing 3 | -1.05 | 0.000163 | 0.012858 |
| HS3ST6 | 540893 | heparan sulfate-glucosamine 3-sulfotransferase 6 | -1.05 | 0.00021 | 0.014718 |
| LOC100847791 | 100847791 | uncharacterized LOC100847791 | -1.04 | 5.34E-07 | 0.000844 |
| LOC112446653 | 112446653 | uncharacterized LOC112446653 | -1.04 | 0.000146 | 0.012433 |
| ADGRA1 | 518385 | adhesion G protein-coupled receptor A1 | -1.04 | 0.001233 | 0.030973 |
| MBD3 | 616090 | methyl-CpG binding domain protein 3 | -1.04 | 0.003201 | 0.046996 |
| MIDN | 523615 | midnolin | -1.04 | 8.17E-06 | 0.003862 |
| LOC789029 | 789029 | T-cell receptor alpha chain V region HPB-MLT-like | -1.03 | 0.001706 | 0.035362 |
| EEIG1 | 615907 | estrogen-induced osteoclastogenesis regulator 1 | -1.02 | 7.51E-05 | 0.009586 |
| LOC132346380 | 132346380 | uncharacterized LOC132346380 | -1.02 | 0.002868 | 0.044702 |
| H3C13 | 504599 | H3 clustered histone 13 | -1.02 | 0.000935 | 0.027707 |
| LOC112449095 | 112449095 | uncharacterized LOC112449095 | -1.02 | 0.002705 | 0.043668 |
| H4C16 | 530773 | H4 histone 16 | -1.02 | 0.000515 | 0.021952 |
| THBS1 | 281530 | thrombospondin 1 | -1.02 | 0.003474 | 0.049102 |
| LZTS1 | 539634 | leucine zipper tumor suppressor 1 | -1.02 | 0.000521 | 0.022152 |
| LOC100140121 | 100140121 | uncharacterized LOC100140121 | -1.01 | 0.002486 | 0.042157 |
| H4C2 | 280691 | H4 clustered histone 2 | -1.01 | 0.00087 | 0.026803 |
| LOC132345170 | 132345170 | uncharacterized LOC132345170 | -1.01 | 0.00252 | 0.042326 |
| GATA3 | 505169 | GATA binding protein 3 | -1.01 | 2.12E-05 | 0.005349 |
| ATG2A | 529808 | autophagy related 2A | -1.01 | 0.000243 | 0.015798 |
| LOC132343948 | 132343948 | myosin IC heavy chain | -1.01 | 0.002964 | 0.045299 |
| LOC616782 | 616782 | uncharacterized LOC616782 | -1.00 | 0.001272 | 0.031343 |
| RIN3 | 539951 | Ras and Rab interactor 3 | -1.00 | 0.002439 | 0.041846 |
| H3C2 | 788250 | H3 clustered histone 2 | -1.00 | 0.000495 | 0.021578 |
| LOC112447188 | 112447188 | small Cajal body-specific RNA 18 | -0.99 | 0.000676 | 0.024428 |
| ANKRD63 | 100140532 | ankyrin repeat domain 63 | -0.99 | 0.000267 | 0.016387 |
| ZNF526 | 514418 | zinc finger protein 526 | -0.99 | 1.38E-06 | 0.001557 |
| CABIN1 | 530023 | calcineurin binding protein 1 | -0.99 | 4.17E-05 | 0.007513 |
| LOC132345501 | 132345501 | uncharacterized LOC132345501 | -0.98 | 1.25E-05 | 0.004479 |
| KHSRP | 505564 | KH-type splicing regulatory protein | -0.97 | 0.002578 | 0.042788 |
| MAPT | 281296 | microtubule associated protein tau | -0.97 | 0.001167 | 0.030029 |
| SHF | 512726 | Src homology 2 domain containing F | -0.97 | 0.002039 | 0.038553 |
| LOC132342366 | 132342366 | uncharacterized LOC132342366 | -0.96 | 0.001686 | 0.035292 |
| LOC101907807 | 101907807 | uncharacterized LOC101907807 | -0.96 | 0.002769 | 0.044198 |
| GREB1 | 508204 | growth regulating estrogen receptor binding 1 | -0.96 | 0.00203 | 0.038473 |
| IZUMO4 | 510569 | IZUMO family member 4 | -0.96 | 0.000575 | 0.022654 |
| SOX13 | 788459 | SRY-box transcription factor 13 | -0.96 | 0.000341 | 0.018157 |
| EFNB1 | 534413 | ephrin B1 | -0.95 | 0.000412 | 0.019669 |
| ZNF580 | 507619 | zinc finger protein 580 | -0.95 | 0.002044 | 0.038583 |
| TMPRSS2 | 511037 | transmembrane serine protease 2 | -0.95 | 0.000968 | 0.028005 |
| FAM170B | 615859 | family with sequence similarity 170 member B | -0.95 | 3.38E-05 | 0.006857 |
| H2AC21 | 614974 | H2A clustered histone 21 | -0.95 | 6.98E-05 | 0.009586 |
| MAST1 | 539825 | microtubule associated serine/threonine kinase 1 | -0.95 | 0.000762 | 0.025204 |
| LOC101905357 | 101905357 | uncharacterized LOC101905357 | -0.94 | 0.000494 | 0.021578 |
| CCNK | 530744 | cyclin K | -0.93 | 0.000442 | 0.020431 |
| ZYX | 768226 | zyxin | -0.92 | 0.001425 | 0.032903 |
| LOC112448415 | 112448415 | T cell receptor delta variable 1-like | -0.92 | 0.000219 | 0.01499 |
| SMAD7 | 535916 | SMAD family member 7 | -0.92 | 0.002415 | 0.041846 |
| TXLNA | 511331 | taxilin alpha | -0.91 | 0.002423 | 0.041846 |
| LOC101902944 | 101902944 | uncharacterized LOC101902944 | -0.91 | 0.00069 | 0.024489 |
| LOC112445076 | 112445076 | polyadenylate-binding protein 1-like | -0.91 | 0.000838 | 0.026175 |
| CHRNB3 | 521702 | cholinergic receptor nicotinic beta 3 subunit | -0.91 | 0.000288 | 0.016905 |
| LOC101903068 | 101903068 | uncharacterized LOC101903068 | -0.90 | 0.00079 | 0.025542 |
| LOC107133209 | 107133209 | uncharacterized LOC107133209 | -0.90 | 0.001109 | 0.029169 |
| FAM25A | 781772 | family with sequence similarity 25 member A | -0.90 | 0.000136 | 0.012038 |
| LOC112447080 | 112447080 | uncharacterized LOC112447080 | -0.90 | 0.002377 | 0.041515 |
| PLXNB1 | 616798 | plexin B1 | -0.90 | 0.000546 | 0.022597 |
| KCNH8 | 100336609 | potassium voltage-gated channel subfamily H member 8 | -0.90 | 0.000778 | 0.02545 |
| DYNLT4 | 516323 | dynein light chain Tctex-type 4 | -0.90 | 3.16E-05 | 0.006475 |
| PTMS | 613777 | parathymosin | -0.89 | 0.00014 | 0.012178 |
| LOC786796 | 786796 | antigen WC1.1 | -0.89 | 0.001403 | 0.032897 |
| ALPK3 | 516866 | alpha kinase 3 | -0.89 | 0.000952 | 0.027954 |
| LOC100299277 | 100299277 | uncharacterized LOC100299277 | -0.89 | 8.86E-05 | 0.009785 |
| LOC101906008 | 101906008 | uncharacterized LOC101906008 | -0.89 | 0.000599 | 0.022803 |
| MSRB1 | 618441 | methionine sulfoxide reductase B1 | -0.88 | 0.00028 | 0.016662 |
| RPS6KA2 | 517953 | ribosomal protein S6 kinase A2 | -0.88 | 2.6E-05 | 0.005943 |
| HROB | 615299 | homologous recombination factor with OB-fold | -0.88 | 0.00115 | 0.02984 |
| LOC112443180 | 112443180 | collagen alpha-1(I) chain | -0.88 | 0.000712 | 0.024626 |
| H4C7 | 115945170 | H4 clustered histone 7 | -0.88 | 0.001816 | 0.036458 |
| GABBR1 | 513785 | gamma-aminobutyric acid type B receptor subunit 1 | -0.88 | 0.000193 | 0.013898 |
| CTSD | 282883 | cathepsin D | -0.88 | 0.00209 | 0.039013 |
| LOC132343352 | 132343352 | uncharacterized LOC132343352 | -0.87 | 0.000589 | 0.022759 |
| PGGHG | 511919 | protein-glucosylgalactosylhydroxylysine glucosidase | -0.87 | 9.72E-06 | 0.003862 |
| MYCBPAP | 505551 | MYCBP associated protein | -0.87 | 0.003199 | 0.046996 |
| TLE3 | 514326 | TLE family member 3, transcriptional corepressor | -0.87 | 3.73E-05 | 0.007012 |
| LOC112442700 | 112442700 | uncharacterized LOC112442700 | -0.86 | 1.97E-05 | 0.005289 |
| ABL1 | 540876 | ABL proto-onco 1, non-receptor tyrosine kinase | -0.86 | 0.000259 | 0.016343 |
| LOC104974667 | 104974667 | uncharacterized LOC104974667 | -0.86 | 0.000279 | 0.016662 |
| LOC521580 | 521580 | histone H2B type 2-F | -0.85 | 8.49E-05 | 0.009724 |
| AEBP1 | 317693 | AE binding protein 1 | -0.85 | 0.002881 | 0.044702 |
| ADCYAP1R1 | 319095 | ADCYAP receptor type I | -0.85 | 0.00065 | 0.023727 |
| SPINT2 | 507484 | serine peptidase inhibitor, Kunitz type 2 | -0.85 | 9.43E-05 | 0.009785 |
| LOC132342993 | 132342993 | uncharacterized LOC132342993 | -0.85 | 0.002342 | 0.041332 |
| H1-10 | 511901 | H1.10 linker histone | -0.85 | 2.13E-05 | 0.005349 |
| BAHCC1 | 786025 | BAH domain and coiled-coil containing 1 | -0.85 | 2.59E-05 | 0.005943 |
| LOC781726 | 781726 | T-cell surface glycoprotein CD1b-2-like | -0.85 | 0.002946 | 0.045196 |
| ZNF48 | 512477 | zinc finger protein 48 | -0.85 | 0.000698 | 0.024489 |
| LOC112449590 | 112449590 | uncharacterized LOC112449590 | -0.84 | 0.000467 | 0.020893 |
| IFITM2 | 615833 | interferon induced transmembrane protein 2 (1-8D) | -0.84 | 3.57E-05 | 0.00688 |
| C19H17orf67 | 100295656 | chromosome 19 C17orf67 homolog | -0.84 | 0.001507 | 0.033269 |
| ZCWPW1 | 514550 | zinc finger CW-type and PWWP domain containing 1 | -0.84 | 0.000524 | 0.022152 |
| PRKN | 530858 | parkin RBR E3 ubiquitin protein ligase | -0.84 | 0.00123 | 0.03097 |
| LOC112446016 | 112446016 | uncharacterized LOC112446016 | -0.84 | 0.002116 | 0.039204 |
| VSIR | 783068 | V-set immunoregulatory receptor | -0.83 | 2.92E-05 | 0.006286 |
| EIF4EBP1 | 509613 | eukaryotic translation initiation factor 4E binding protein 1 | -0.83 | 0.003313 | 0.047686 |
| AFAP1 | 534032 | actin filament associated protein 1 | -0.83 | 0.000322 | 0.017809 |
| PDE9A | 511665 | phosphodiesterase 9A | -0.83 | 0.001257 | 0.031221 |
| LOC112444214 | 112444214 | small Cajal body-specific RNA 18 | -0.83 | 0.000445 | 0.020431 |
| RAD23A | 540564 | RAD23 homolog A, nucleotide excision repair protein | -0.83 | 0.002821 | 0.044354 |
| THTPA | 282090 | thiamine triphosphatase | -0.82 | 0.00347 | 0.049102 |
| FANCD2OS | 768064 | FANCD2 opposite strand | -0.82 | 0.001877 | 0.036987 |
| LOC112442425 | 112442425 | small nucleolar RNA, C/D box 35A | -0.82 | 2.35E-05 | 0.005635 |
| C11H9orf50 | 617008 | chromosome 11 C9orf50 homolog | -0.82 | 0.002617 | 0.043268 |
| LRP2BP | 536490 | LRP2 binding protein | -0.82 | 0.002101 | 0.039155 |
| LOC100299845 | 100299845 | large ribosomal subunit protein uL18-like | -0.81 | 0.003519 | 0.049513 |
| SLC16A6 | 529171 | solute carrier family 16 member 6 | -0.81 | 2.13E-05 | 0.005349 |
| LOC112444215 | 112444215 | small Cajal body-specific RNA 17 | -0.81 | 0.00053 | 0.022235 |
| TPRA1 | 617772 | transmembrane protein adipocyte associated 1 | -0.81 | 0.003148 | 0.0468 |
| MIR484 | 790989 | bta-mir-484 | -0.81 | 0.000279 | 0.016662 |
| H2BC20 | 506306 | H2B clustered histone 20 | -0.81 | 0.000113 | 0.010753 |
| CHD4 | 506402 | chromodomain helicase DNA binding protein 4 | -0.81 | 0.001491 | 0.033269 |
| EHMT2 | 514062 | euchromatic histone lysine methyltransferase 2 | -0.81 | 0.003139 | 0.0468 |
| CIRBP | 507120 | cold inducible RNA binding protein | -0.80 | 1.82E-07 | 0.000575 |
| EFHD2 | 514259 | EF-hand domain family member D2 | -0.80 | 0.000193 | 0.013898 |
| H2BC11 | 522960 | H2B clustered histone 11 | -0.80 | 0.001218 | 0.03097 |
| L1CAM | 516017 | L1 cell adhesion molecule | -0.80 | 0.000323 | 0.017809 |
| TNRC18 | 526616 | trinucleotide repeat containing 18 | -0.80 | 0.00075 | 0.025204 |
| MIOX | 508591 | myo-inositol oxygenase | -0.79 | 0.002091 | 0.039013 |
| SDF4 | 528783 | stromal cell derived factor 4 | -0.79 | 7.39E-05 | 0.009586 |
| KLC2 | 518136 | kinesin light chain 2 | -0.79 | 0.003116 | 0.046543 |
| ANGEL1 | 508197 | angel homolog 1 | -0.79 | 2.9E-06 | 0.002576 |
| FAM83H | 524974 | family with sequence similarity 83 member H | -0.79 | 0.000447 | 0.020431 |
| ATP2A3 | 512313 | ATPase sarcoplasmic/endoplasmic reticulum Ca2+ transporting 3 | -0.79 | 0.001731 | 0.035555 |
| LIMS2 | 515401 | LIM zinc finger domain containing 2 | -0.78 | 0.000825 | 0.025979 |
| ABCD1 | 515178 | ATP binding cassette subfamily D member 1 | -0.78 | 0.000178 | 0.013421 |
| LOC132344462 | 132344462 | P2Y receptor family member 8 | -0.78 | 0.000158 | 0.012705 |
| YKT6 | 507409 | YKT6 v-SNARE homolog | -0.78 | 0.000219 | 0.01499 |
| ABCA1 | 535379 | ATP binding cassette subfamily A member 1 | -0.78 | 0.001602 | 0.034217 |
| RBM3 | 509771 | RNA binding motif protein 3 | -0.77 | 4.79E-07 | 0.00084 |
| NAA80 | 104968404 | N-alpha-acetyltransferase 80, NatH catalytic subunit | -0.77 | 0.00011 | 0.010685 |
| MYL5 | 101907739 | myosin light chain 5 | -0.77 | 0.000137 | 0.012136 |
| CYGB | 510299 | cytoglobin | -0.77 | 0.001522 | 0.033308 |
| ABI3 | 529835 | ABI family member 3 | -0.77 | 0.001711 | 0.035362 |
| LOC132343640 | 132343640 | uncharacterized LOC132343640 | -0.77 | 0.001561 | 0.033844 |
| RAB8A | 100125881 | RAB8A, member RAS onco family | -0.77 | 0.000753 | 0.025204 |
| LOC112442265 | 112442265 | uncharacterized LOC112442265 | -0.76 | 0.001005 | 0.02844 |
| XRRA1 | 369019 | X-ray radiation resistance associated 1 | -0.76 | 0.000892 | 0.027204 |
| IFT43 | 513228 | intraflagellar transport 43 | -0.76 | 6.8E-05 | 0.009576 |
| XCR1 | 617880 | X-C motif chemokine receptor 1 | -0.76 | 0.001712 | 0.035362 |
| SEC14L2 | 282469 | SEC14 like lipid binding 2 | -0.75 | 0.000748 | 0.025193 |
| NUDT16L1 | 518085 | nudix hydrolase 16 like 1 | -0.75 | 6.73E-05 | 0.009576 |
| JUP | 445543 | junction plakoglobin | -0.75 | 0.001286 | 0.031446 |
| LOC101905262 | 101905262 | uncharacterized LOC101905262 | -0.75 | 0.000449 | 0.020431 |
| EMID1 | 506282 | EMI domain containing 1 | -0.75 | 0.00025 | 0.01586 |
| CSK | 509246 | C-terminal Src kinase | -0.75 | 8.3E-05 | 0.009724 |
| UCP2 | 281562 | uncoupling protein 2 | -0.75 | 0.000835 | 0.026175 |
| UPK1A | 282112 | uroplakin 1A | -0.75 | 0.00152 | 0.033308 |
| IQCN | 788637 | IQ motif containing N | -0.75 | 2.93E-06 | 0.002576 |
| SDK1 | 516333 | sidekick cell adhesion molecule 1 | -0.75 | 0.001928 | 0.037546 |
| CRYBB1 | 282205 | crystallin beta B1 | -0.74 | 0.000223 | 0.015078 |
| HSPB6 | 534551 | heat shock protein family B (small) member 6 | -0.74 | 0.002797 | 0.04434 |
| PIANP | 614601 | PILR alpha associated neural protein | -0.74 | 0.000915 | 0.027508 |
| PYGM | 327664 | glycogen phosphorylase, muscle associated | -0.74 | 0.000588 | 0.022759 |
| NQO2 | 508566 | N-ribosyldihydronicotinamide:quinone dehydrogenase 2 | -0.74 | 4.3E-05 | 0.007543 |
| ZP3 | 280964 | zona pellucida glycoprotein 3 | -0.74 | 0.000682 | 0.024458 |
| LOC132342615 | 132342615 | uncharacterized LOC132342615 | -0.74 | 0.001245 | 0.031105 |
| DCP2 | 539770 | decapping mRNA 2 | -0.74 | 0.001219 | 0.03097 |
| C1QTNF6 | 506413 | C1q and TNF related 6 | -0.74 | 0.001634 | 0.034526 |
| PRPF6 | 534228 | pre-mRNA processing factor 6 | -0.73 | 0.000381 | 0.018767 |
| UBTF | 497012 | upstream binding transcription factor | -0.73 | 0.002424 | 0.041846 |
| CMTR1 | 509620 | cap methyltransferase 1 | -0.73 | 0.002775 | 0.044198 |
| ZNF687 | 787675 | zinc finger protein 687 | -0.73 | 0.002221 | 0.040116 |
| H2BC9 | 104968456 | H2B clustered histone 9 | -0.73 | 0.000758 | 0.025204 |
| RAB11FIP4 | 100848380 | RAB11 family interacting protein 4 | -0.72 | 0.000167 | 0.013089 |
| LOC112448841 | 112448841 | uncharacterized LOC112448841 | -0.72 | 0.001484 | 0.033269 |
| MIR339A | 100313035 | bta-mir-339a | -0.72 | 0.000797 | 0.025606 |
| LOC132346145 | 132346145 | basic proline-rich protein-like | -0.72 | 0.001983 | 0.038073 |
| LOC107133207 | 107133207 | uncharacterized LOC107133207 | -0.72 | 0.001336 | 0.032301 |
| RTN2 | 359720 | reticulon 2 | -0.72 | 0.00316 | 0.046841 |
| DHRS12 | 507276 | dehydrogenase/reductase 12 | -0.72 | 0.003564 | 0.049839 |
| EPPK1 | 100337278 | epiplakin 1 | -0.72 | 0.001112 | 0.029181 |
| SEPTIN5 | 615408 | septin 5 | -0.72 | 0.001609 | 0.034277 |
| PVRIG | 514026 | PVR related immunoglobulin domain containing | -0.72 | 0.000338 | 0.018151 |
| TSC22D4 | 524543 | TSC22 domain family member 4 | -0.71 | 0.003206 | 0.046996 |
| C15H11orf71 | 615529 | chromosome 15 C11orf71 homolog | -0.71 | 2.03E-05 | 0.005336 |
| TAF4 | 789854 | TATA-box binding protein associated factor 4 | -0.71 | 0.000772 | 0.025304 |
| INTS5 | 514150 | integrator complex subunit 5 | -0.71 | 0.000322 | 0.017809 |
| IFITM1 | 353510 | interferon induced transmembrane protein 1 (9-27) | -0.71 | 0.002221 | 0.040116 |
| MYH9 | 404108 | myosin heavy chain 9 | -0.71 | 0.000454 | 0.020559 |
| LOC104972363 | 104972363 | uncharacterized LOC104972363 | -0.70 | 0.001938 | 0.037625 |
| H2AC25 | 538911 | H2A clustered histone 25 | -0.70 | 0.001045 | 0.028926 |
| PRKD2 | 782793 | protein kinase D2 | -0.70 | 0.000595 | 0.022771 |
| TTYH3 | 512271 | tweety family member 3 | -0.70 | 0.001222 | 0.03097 |
| LOC100848642 | 100848642 | uncharacterized LOC100848642 | -0.70 | 0.001246 | 0.031105 |
| PLEKHG2 | 504530 | pleckstrin homology and RhoGEF domain containing G2 | -0.70 | 0.000282 | 0.016662 |
| RABIF | 616779 | RAB interacting factor | -0.70 | 0.00039 | 0.019083 |
| HSF1 | 506235 | heat shock transcription factor 1 | -0.70 | 0.00053 | 0.022235 |
| CACNA1S | 100337204 | calcium voltage-gated channel subunit alpha1 S | -0.70 | 0.001814 | 0.036458 |
| NUPR2 | 614047 | nuclear protein 2, transcriptional regulator | -0.70 | 4.18E-05 | 0.007513 |
| LOC132345761 | 132345761 | protein diaphanous homolog 1 | -0.70 | 0.003043 | 0.045718 |
| LOC132342368 | 132342368 | uncharacterized LOC132342368 | -0.70 | 0.000501 | 0.021578 |
| CARS1 | 515715 | cysteinyl-tRNA synthetase 1 | -0.70 | 0.002022 | 0.038398 |
| ALDOA | 509566 | aldolase, fructose-bisphosphate A | -0.69 | 0.000233 | 0.015399 |
| LOC504773 | 504773 | regakine 1 | -0.69 | 0.001724 | 0.035476 |
| BRAT1 | 532550 | BRCA1 associated ATM activator 1 | -0.69 | 0.002881 | 0.044702 |
| NOTCH1 | 767866 | notch receptor 1 | -0.69 | 2.22E-05 | 0.005417 |
| ITGAL | 281874 | integrin subunit alpha L | -0.69 | 0.000567 | 0.022597 |
| NIBAN2 | 613747 | niban apoptosis regulator 2 | -0.69 | 0.003541 | 0.049691 |
| LIME1 | 100140162 | Lck interacting transmembrane adaptor 1 | -0.69 | 0.000343 | 0.018179 |
| LOC101906086 | 101906086 | uncharacterized LOC101906086 | -0.68 | 0.001961 | 0.037853 |
| CERK | 100336737 | ceramide kinase | -0.68 | 7.52E-05 | 0.009586 |
| ZKSCAN1 | 100140434 | zinc finger with KRAB and SCAN domains 1 | -0.68 | 0.002647 | 0.043503 |
| GSTZ1 | 514822 | glutathione S-transferase zeta 1 | -0.68 | 0.00047 | 0.020906 |
| RNPEPL1 | 511497 | arginyl aminopeptidase like 1 | -0.68 | 0.000539 | 0.02255 |
| CDH3 | 281063 | cadherin 3 | -0.68 | 0.00014 | 0.012178 |
| PBX2 | 100139739 | PBX homeobox 2 | -0.68 | 0.003289 | 0.047598 |
| CTDSP1 | 516199 | CTD small phosphatase 1 | -0.68 | 8.8E-06 | 0.003862 |
| IGHMBP2 | 618606 | immunoglobulin mu DNA binding protein 2 | -0.68 | 3.98E-06 | 0.00286 |
| SMPD4 | 507207 | sphingomyelin phosphodiesterase 4 | -0.67 | 0.000429 | 0.020298 |
| LOC101905779 | 101905779 | uncharacterized LOC101905779 | -0.67 | 0.001414 | 0.032903 |
| IGF2R | 281849 | insulin like growth factor 2 receptor | -0.67 | 0.00014 | 0.012178 |
| LOC132344829 | 132344829 | uncharacterized LOC132344829 | -0.67 | 0.000483 | 0.021437 |
| LOC132345358 | 132345358 | uncharacterized LOC132345358 | -0.67 | 0.00317 | 0.04685 |
| NBEAL2 | 788207 | neurobeachin like 2 | -0.67 | 0.000274 | 0.016601 |
| ATXN7L3 | 525252 | ataxin 7 like 3 | -0.67 | 0.000683 | 0.024458 |
| LOC617475 | 617475 | coagulation factor VIII-associated 1-like | -0.67 | 0.000378 | 0.018767 |
| WDR86 | 100140069 | WD repeat domain 86 | -0.67 | 0.001708 | 0.035362 |
| CD82 | 506713 | CD82 molecule | -0.67 | 0.001828 | 0.03657 |
| SLC6A6 | 282366 | solute carrier family 6 member 6 | -0.67 | 0.000156 | 0.012705 |
| SNAI3 | 526694 | snail family transcriptional repressor 3 | -0.67 | 0.002301 | 0.040909 |
| ENTPD2 | 100126045 | ectonucleoside triphosphate diphosphohydrolase 2 | -0.66 | 0.000373 | 0.018767 |
| PREX1 | 527410 | phosphatidylinositol-3,4,5-trisphosphate dependent Rac exchange factor 1 | -0.66 | 9.94E-05 | 0.010197 |
| EMP3 | 535273 | epithelial membrane protein 3 | -0.66 | 0.0015 | 0.033269 |
| RORC | 527470 | RAR related orphan receptor C | -0.66 | 0.001897 | 0.037231 |
| PPM1N | 530233 | protein phosphatase, Mg2+/Mn2+ dependent 1N (putative) | -0.66 | 0.001657 | 0.034865 |
| CIRBP | 507120 | cold inducible RNA binding protein | -0.65 | 8.62E-06 | 0.043531 |
| CRACR2B | 615055 | calcium release activated channel regulator 2B | -0.65 | 0.002163 | 0.039557 |
| FBRS | 615050 | fibrosin | -0.65 | 0.000905 | 0.027394 |
| MMP24OS | 112449330 | MMP24 opposite strand | -0.65 | 0.00017 | 0.013096 |
| PARP1 | 286764 | poly(ADP-ribose) polymerase 1 | -0.65 | 0.002766 | 0.044198 |
| LOC100335608 | 100335608 | uncharacterized LOC100335608 | -0.65 | 0.000263 | 0.016343 |
| POLD1 | 281990 | DNA polymerase delta 1, catalytic subunit | -0.64 | 0.002052 | 0.038702 |
| CD7 | 510073 | CD7 molecule | -0.64 | 0.003163 | 0.046841 |
| ARL8A | 101906089 | ADP ribosylation factor like GTPase 8A | -0.64 | 0.001154 | 0.029884 |
| ALKBH6 | 539257 | alkB homolog 6 | -0.64 | 0.000567 | 0.022597 |
| AGFG2 | 510361 | ArfGAP with FG repeats 2 | -0.64 | 0.002777 | 0.044198 |
| DBP | 503577 | D-box binding PAR bZIP transcription factor | -0.64 | 0.002959 | 0.045299 |
| MRPS2 | 505681 | mitochondrial ribosomal protein S2 | -0.63 | 0.002839 | 0.044539 |
| KLF8 | 525558 | KLF transcription factor 8 | -0.63 | 0.001848 | 0.036665 |
| DAPK1 | 540873 | death associated protein kinase 1 | -0.63 | 0.000451 | 0.020499 |
| PPP1R3F | 513859 | protein phosphatase 1 regulatory subunit 3F | -0.63 | 0.001996 | 0.038196 |
| SYCE2 | 100141142 | synaptonemal complex central element protein 2 | -0.63 | 0.003393 | 0.048343 |
| LOC132342072 | 132342072 | 2-oxoglutarate dehydrogenase complex component E1-like | -0.63 | 0.000498 | 0.021578 |
| CLEC16A | 615054 | C-type lectin domain containing 16A | -0.63 | 7.67E-05 | 0.009586 |
| PPP1R10 | 510825 | protein phosphatase 1 regulatory subunit 10 | -0.62 | 0.000581 | 0.022759 |
| LOC513969 | 513969 | ankyrin repeat domain-containing protein 26 | -0.62 | 0.002447 | 0.041846 |
| SPA17 | 616974 | sperm autoantigenic protein 17 | -0.62 | 0.003223 | 0.047158 |
| STAG3 | 515399 | STAG3 cohesin complex component | -0.62 | 0.000112 | 0.010748 |
| ACCS | 505649 | 1-aminocyclopropane-1-carboxylate synthase homolog (inactive) | -0.61 | 0.001784 | 0.036246 |
| PTMA | 786336 | prothymosin alpha | -0.61 | 0.003008 | 0.04545 |
| AMBRA1 | 517263 | autophagy and beclin 1 regulator 1 | -0.61 | 0.000168 | 0.013096 |
| TSNARE1 | 535306 | t-SNARE domain containing 1 | -0.61 | 8.04E-05 | 0.009646 |
| SAMD10 | 540999 | sterile alpha motif domain containing 10 | -0.61 | 0.000736 | 0.024941 |
| PLEC | 786966 | plectin | -0.61 | 0.001692 | 0.035359 |
| IQCE | 618833 | IQ motif containing E | -0.61 | 0.003113 | 0.046542 |
| ARHGEF18 | 522521 | Rho/Rac guanine nucleotide exchange factor 18 | -0.61 | 0.001471 | 0.033269 |
| LOC132343398 | 132343398 | 5E5 antigen-like | -0.60 | 0.000921 | 0.02756 |
| TAF3 | 506674 | TATA-box binding protein associated factor 3 | -0.60 | 0.001353 | 0.032592 |
| SLC13A5 | 507000 | solute carrier family 13 member 5 | -0.60 | 0.002366 | 0.041437 |
| TUBB3 | 768070 | tubulin beta 3 class III | -0.60 | 0.000856 | 0.026632 |
| LARP1 | 505690 | La ribonucleoprotein 1, translational regulator | -0.60 | 0.002987 | 0.045385 |
| SEPTIN9 | 100140583 | septin 9 | -0.59 | 5.62E-05 | 0.008663 |
| THA1 | 507443 | threonine aldolase 1 | -0.59 | 0.000179 | 0.013421 |
| TMEM134 | 510129 | transmembrane protein 134 | -0.59 | 1.71E-05 | 0.004992 |
| NCBP2AS2 | 783161 | NCBP2 antisense 2 (head to head) | -0.59 | 0.002356 | 0.041362 |
| CAPN10 | 789674 | calpain 10 | -0.59 | 0.000246 | 0.01586 |
| PHYKPL | 537241 | 5-phosphohydroxy-L-lysine phospho-lyase | -0.59 | 1.45E-05 | 0.004866 |
| PIDD1 | 100137737 | p53-induced death domain protein 1 | -0.59 | 0.000262 | 0.016343 |
| ADD1 | 507193 | adducin 1 | -0.59 | 0.000273 | 0.0166 |
| LOC101902444 | 101902444 | uncharacterized LOC101902444 | -0.59 | 0.000498 | 0.021578 |
| NUDT1 | 525496 | nudix hydrolase 1 | -0.58 | 0.003241 | 0.047295 |
| B3GALT4 | 768038 | beta-1,3-galactosyltransferase 4 | -0.58 | 0.00097 | 0.028005 |
| PODNL1 | 534549 | podocan like 1 | -0.58 | 0.000439 | 0.020431 |
| PDAP1 | 100139074 | PDGFA associated protein 1 | -0.58 | 0.002994 | 0.045397 |
| BOLA1 | 509530 | bolA family member 1 | -0.58 | 0.000404 | 0.019423 |
| DBN1 | 505406 | drebrin 1 | -0.58 | 0.000807 | 0.025736 |
| OGDH | 534599 | oxoglutarate dehydrogenase | -0.58 | 0.000799 | 0.025606 |
| LCN2 | 526639 | lipocalin 2 | -0.58 | 3.57E-05 | 0.00688 |
| MICALL1 | 517259 | MICAL like 1 | -0.58 | 0.001467 | 0.033269 |
| AMDHD2 | 521401 | amidohydrolase domain containing 2 | -0.58 | 5.87E-06 | 0.003433 |
| STK40 | 515723 | serine/threonine kinase 40 | -0.58 | 3.25E-06 | 0.002635 |
| OMG | 407186 | oligodendrocyte myelin glycoprotein | -0.58 | 0.001886 | 0.037068 |
| CALM3 | 520277 | calmodulin 3 | -0.58 | 0.003162 | 0.046841 |
| VPS28 | 618785 | VPS28 subunit of ESCRT-I | -0.57 | 0.000593 | 0.022771 |
| PABPC4 | 534576 | poly(A) binding protein cytoplasmic 4 | -0.57 | 0.00061 | 0.023023 |
| B4GALT2 | 100125390 | beta-1,4-galactosyltransferase 2 | -0.57 | 0.001717 | 0.035378 |
| PARP10 | 510991 | poly(ADP-ribose) polymerase family member 10 | -0.57 | 0.000149 | 0.012596 |
| SEC61A1 | 505064 | SEC61 translocon subunit alpha 1 | -0.57 | 0.000193 | 0.013898 |
| USP30 | 100140210 | ubiquitin specific peptidase 30 | -0.57 | 0.001717 | 0.035378 |
| PGLYRP2 | 510803 | peptidoglycan recognition protein 2 | -0.57 | 0.000806 | 0.025736 |
| SPTBN2 | 132344210 | spectrin beta, non-erythrocytic 2 | -0.57 | 0.001172 | 0.030103 |
| P2RX3 | 530468 | purinergic receptor P2X 3 | -0.57 | 0.00082 | 0.025908 |
| SLC9A8 | 617800 | solute carrier family 9 member A8 | -0.56 | 0.000243 | 0.015798 |
| DDT | 615999 | D-dopachrome tautomerase | -0.56 | 0.000901 | 0.027353 |
| ZMIZ1 | 616740 | zinc finger MIZ-type containing 1 | -0.56 | 0.003021 | 0.045475 |
| ZNF646 | 783820 | zinc finger protein 646 | -0.56 | 0.002223 | 0.040116 |
| PDK2 | 524075 | pyruvate dehydrogenase kinase 2 | -0.56 | 0.000171 | 0.013096 |
| DNAL4 | 618379 | dynein axonemal light chain 4 | -0.56 | 0.002705 | 0.043668 |
| FAAP20 | 508039 | FA core complex associated protein 20 | -0.56 | 0.000824 | 0.025979 |
| WASF2 | 504482 | WASP family member 2 | -0.55 | 0.001963 | 0.037853 |
| NPRL3 | 534485 | NPR3 like, GATOR1 complex subunit | -0.55 | 8.4E-05 | 0.009724 |
| GAS8 | 504318 | growth arrest specific 8 | -0.55 | 0.000443 | 0.020431 |
| TIGD5 | 101903505 | tigger transposable element derived 5 | -0.55 | 0.00201 | 0.038265 |
| COQ4 | 511987 | coenzyme Q4 | -0.55 | 0.000143 | 0.0123 |
| TMEM250 | 784805 | transmembrane protein 250 | -0.55 | 0.00254 | 0.042459 |
| EME2 | 515662 | essential meiotic structure-specific endonuclease subunit 2 | -0.55 | 0.00073 | 0.02491 |
| GALNS | 515809 | galactosamine (N-acetyl)-6-sulfatase | -0.55 | 7.95E-05 | 0.009646 |
| IDUA | 511050 | alpha-L-iduronidase | -0.55 | 0.000107 | 0.010616 |
| CSKMT | 512401 | citrate synthase lysine methyltransferase | -0.54 | 0.002 | 0.038216 |
| TNK2 | 280710 | tyrosine kinase non receptor 2 | -0.54 | 0.002894 | 0.044789 |
| RHBDD3 | 507674 | rhomboid domain containing 3 | -0.54 | 0.00248 | 0.042106 |
| FXYD5 | 505584 | FXYD domain containing ion transport regulator 5 | -0.54 | 0.000341 | 0.018157 |
| SLC25A10 | 100848134 | solute carrier family 25 member 10 | -0.54 | 0.000558 | 0.022597 |
| CPSF7 | 504925 | cleavage and polyadenylation specific factor 7 | -0.54 | 0.000877 | 0.026869 |
| MYL6B | 515421 | myosin light chain 6B | -0.54 | 0.000603 | 0.022901 |
| EFCAB11 | 617365 | EF-hand calcium binding domain 11 | -0.54 | 0.000325 | 0.017809 |
| HINT2 | 281816 | histidine triad nucleotide binding protein 2 | -0.54 | 0.000348 | 0.018269 |
| ASXL1 | 522091 | ASXL transcriptional regulator 1 | -0.53 | 0.001127 | 0.029444 |
| GANC | 530330 | glucosidase alpha, neutral C | -0.53 | 0.001465 | 0.033269 |
| KDM3B | 100848816 | lysine demethylase 3B | -0.53 | 0.002688 | 0.043618 |
| GPR132 | 539146 | G protein-coupled receptor 132 | -0.53 | 0.002226 | 0.040116 |
| HNRNPD | 527471 | heteroous nuclear ribonucleoprotein D | -0.53 | 7.38E-05 | 0.009586 |
| ZBTB42 | 789062 | zinc finger and BTB domain containing 42 | -0.53 | 0.000332 | 0.017944 |
| RAB11B | 532723 | RAB11B, member RAS onco family | -0.53 | 0.003541 | 0.049691 |
| GANAB | 540155 | glucosidase II alpha subunit | -0.53 | 0.002624 | 0.043328 |
| MAGED1 | 512562 | MAGE family member D1 | -0.53 | 9.08E-05 | 0.009785 |
| TOR4A | 618444 | torsin family 4 member A | -0.53 | 0.000712 | 0.024626 |
| RGS9 | 281453 | regulator of G protein signaling 9 | -0.53 | 0.001346 | 0.032478 |
| TKFC | 512373 | triokinase and FMN cyclase | -0.53 | 8.44E-05 | 0.009724 |
| GPR35 | 505056 | G protein-coupled receptor 35 | -0.53 | 0.000515 | 0.021952 |
| TUBGCP2 | 781376 | tubulin gamma complex associated protein 2 | -0.53 | 0.000187 | 0.013898 |
| CIPC | 506130 | CLOCK interacting pacemaker | -0.52 | 0.002273 | 0.040639 |
| ALAD | 510679 | aminolevulinate dehydratase | -0.52 | 0.002851 | 0.044565 |
| PRRC2B | 505073 | proline rich coiled-coil 2B | -0.52 | 0.003335 | 0.047917 |
| LOC112447418 | 112447418 | uncharacterized LOC112447418 | -0.52 | 0.002737 | 0.044095 |
| CDK10 | 615171 | cyclin dependent kinase 10 | -0.52 | 4.65E-05 | 0.007964 |
| TCF25 | 100848970 | transcription factor 25 | -0.52 | 0.000771 | 0.025304 |
| RBM10 | 505749 | RNA binding motif protein 10 | -0.52 | 0.001365 | 0.032633 |
| TSPAN15 | 522371 | tetraspanin 15 | -0.52 | 0.001429 | 0.032903 |
| DLGAP4 | 520521 | DLG associated protein 4 | -0.52 | 0.001833 | 0.03657 |
| PPP2R1A | 535321 | protein phosphatase 2 scaffold subunit Aalpha | -0.52 | 0.002068 | 0.038901 |
| WDFY1 | 614729 | WD repeat and FYVE domain containing 1 | -0.51 | 0.003144 | 0.0468 |
| STK11IP | 515395 | serine/threonine kinase 11 interacting protein | -0.51 | 0.000377 | 0.018767 |
| TLE1 | 512888 | transducin-like enhancer of split 1 (E(sp1) homolog, Drosophila) | -0.51 | 0.000283 | 0.016686 |
| PRSS33 | 528784 | serine protease 33 | -0.51 | 7.11E-05 | 0.009586 |
| ARRB2 | 281638 | arrestin beta 2 | -0.51 | 0.000809 | 0.025736 |
| BLK | 532587 | BLK proto-onco, Src family tyrosine kinase | -0.51 | 0.002463 | 0.041935 |
| RAB33A | 510101 | RAB33A, member RAS onco family | -0.51 | 0.002165 | 0.039557 |
| MLXIP | 783217 | MLX interacting protein | -0.51 | 0.00087 | 0.026803 |
| COPRS | 506999 | coordinator of PRMT5 and differentiation stimulator | -0.51 | 0.000122 | 0.011386 |
| DPM2 | 523737 | dolichyl-phosphate mannosyltransferase subunit 2, regulatory | -0.51 | 0.000265 | 0.016382 |
| TYSND1 | 101903772 | trypsin like peroxisomal matrix peptidase 1 | -0.51 | 0.0005 | 0.021578 |
| ACTMAP | 509803 | actin maturation protease | -0.51 | 0.000264 | 0.016343 |
| YBEY | 768063 | ybeY metalloendoribonuclease | -0.51 | 0.000368 | 0.018767 |
| FANCA | 618375 | FA complementation group A | -0.50 | 0.000326 | 0.017809 |
| H2AJ | 618489 | H2A.J histone | -0.50 | 0.001229 | 0.03097 |
| MRPL41 | 506521 | mitochondrial ribosomal protein L41 | -0.50 | 0.000971 | 0.028005 |
| CDCA3 | 614434 | cell division cycle associated 3 | -0.50 | 0.000605 | 0.022934 |
| GMDS | 617688 | GDP-mannose 4,6-dehydratase | -0.50 | 0.000866 | 0.026803 |
| NUDC | 513277 | nuclear distribution C, dynein complex regulator | -0.50 | 0.002461 | 0.041935 |
| PLSCR3 | 510355 | phospholipid scramblase 3 | -0.50 | 0.000261 | 0.016343 |
| DIP2C | 535136 | disco interacting C | -0.50 | 0.00143 | 0.032903 |
| ALYREF | 537706 | Aly/REF export factor | -0.50 | 0.00037 | 0.018767 |
| ESS2 | 513758 | ess-2 splicing factor homolog | -0.50 | 0.002657 | 0.043556 |
| LOC112444276 | 112444276 | zinc finger protein 892 | -0.49 | 0.000401 | 0.019305 |
| TMEM187 | 508380 | transmembrane protein 187 | -0.49 | 0.000957 | 0.027954 |
| QSOX1 | 522986 | quiescin sulfhydryl oxidase 1 | -0.49 | 0.000931 | 0.027697 |
| ARHGAP1 | 512817 | Rho GTPase activating protein 1 | -0.49 | 0.000711 | 0.024626 |
| YJU2B | 614791 | YJU2 splicing factor homolog B | -0.49 | 9.45E-05 | 0.009785 |
| R3HDM4 | 614948 | R3H domain containing 4 | -0.49 | 0.002307 | 0.040909 |
| PALM | 786096 | paralemmin | -0.49 | 0.00253 | 0.042449 |
| VMAC | 515212 | vimentin type intermediate filament associated coiled-coil protein | -0.49 | 0.002813 | 0.04434 |
| MMP28 | 519276 | matrix metallopeptidase 28 | -0.49 | 0.002992 | 0.045397 |
| C21H15orf39 | 784903 | chromosome 21 C15orf39 homolog | -0.49 | 0.001652 | 0.034865 |
| SKI | 506127 | SKI proto-oncogene | -0.49 | 0.003311 | 0.047686 |
| LZTS2 | 504411 | leucine zipper tumor suppressor 2 | -0.48 | 0.001733 | 0.035555 |
| LOC112449602 | 112449602 | uncharacterized LOC112449602 | -0.48 | 0.000658 | 0.023973 |
| HTT | 615059 | huntingtin | -0.48 | 0.001844 | 0.036665 |
| TECPR2 | 530121 | tectonin beta-propeller repeat containing 2 | -0.48 | 0.002766 | 0.044198 |
| NFATC2 | 530401 | nuclear factor of activated T cells 2 | -0.48 | 0.001034 | 0.028795 |
| CFAP54 | 787705 | cilia and flagella associated protein 54 | -0.48 | 0.00223 | 0.040144 |
| ST6GALNAC4 | 404124 | ST6 N-acetylgalactosaminide alpha-2,6-sialyltransferase 4 | -0.48 | 0.00109 | 0.029054 |
| SIRT3 | 614027 | sirtuin 3 | -0.48 | 5.76E-05 | 0.008663 |
| PCIF1 | 535479 | phosphorylated CTD interacting factor 1 | -0.48 | 1.94E-05 | 0.005289 |
| NUMA1 | 513091 | nuclear mitotic apparatus protein 1 | -0.48 | 0.000563 | 0.022597 |
| PRPF40B | 513782 | pre-mRNA processing factor 40 homolog B | -0.47 | 0.001381 | 0.032633 |
| KCND1 | 518384 | potassium voltage-gated channel subfamily D member 1 | -0.47 | 0.000818 | 0.025908 |
| SIGIRR | 531801 | single Ig and TIR domain containing | -0.47 | 0.000381 | 0.018767 |
| BCL2L12 | 533338 | BCL2 like 12 | -0.47 | 0.000361 | 0.018656 |
| NDUFB7 | 338065 | NADH:ubiquinone oxidoreductase subunit B7 | -0.47 | 0.003361 | 0.048066 |
| CD99 | 509230 | CD99 molecule | -0.47 | 0.003192 | 0.046996 |
| TMEM80 | 613612 | transmembrane protein 80 | -0.47 | 0.001632 | 0.034516 |
| SCAF8 | 100298738 | SR-related CTD associated factor 8 | -0.47 | 0.001619 | 0.034347 |
| C10H14orf93 | 506904 | chromosome 10 C14orf93 homolog | -0.47 | 7.63E-06 | 0.00377 |
| TCF7 | 782690 | transcription factor 7 | -0.47 | 0.000339 | 0.018157 |
| NSD2 | 540769 | nuclear receptor binding SET domain protein 2 | -0.47 | 0.001428 | 0.032903 |
| ILF3 | 614936 | interleukin enhancer binding factor 3 | -0.46 | 0.000796 | 0.025606 |
| HSH2D | 100848273 | hematopoietic SH2 domain containing | -0.46 | 0.000926 | 0.027596 |
| EEF1G | 326581 | eukaryotic translation elongation factor 1 gamma | -0.46 | 0.002817 | 0.04434 |
| TP73 | 515105 | tumor protein p73 | -0.46 | 0.000934 | 0.027707 |
| CCHCR1 | 514215 | coiled-coil alpha-helical rod protein 1 | -0.46 | 0.002632 | 0.043333 |
| GSE1 | 538506 | Gse1 coiled-coil protein | -0.46 | 0.001606 | 0.034261 |
| POMC | 281416 | proopiomelanocortin | -0.46 | 0.001078 | 0.029051 |
| DAXX | 504336 | death domain associated protein | -0.46 | 0.002545 | 0.042459 |
| MSRB2 | 613475 | methionine sulfoxide reductase B2 | -0.46 | 0.000583 | 0.022759 |
| DNAJC4 | 533579 | DnaJ heat shock protein family (Hsp40) member C4 | -0.45 | 0.001485 | 0.033269 |
| USE1 | 512890 | unconventional SNARE in the ER 1 | -0.45 | 0.000699 | 0.024489 |
| SIN3A | 518504 | SIN3 transcription regulator family member A | -0.45 | 0.002476 | 0.042106 |
| TANC1 | 507983 | tetratricopeptide repeat, ankyrin repeat and coiled-coil containing 1 | -0.45 | 0.001514 | 0.033269 |
| C16H1orf53 | 112441873 | chromosome 16 C1orf53 homolog | -0.45 | 0.003429 | 0.04866 |
| DAZAP1 | 614783 | DAZ associated protein 1 | -0.45 | 0.001598 | 0.034211 |
| ADCK1 | 533372 | aarF domain containing kinase 1 | -0.45 | 0.002127 | 0.039204 |
| KIF22 | 506294 | kinesin family member 22 | -0.45 | 0.002366 | 0.041437 |
| TSPAN14 | 539055 | tetraspanin 14 | -0.45 | 0.000555 | 0.022597 |
| CNTROB | 539106 | centrobin, centriole duplication and spindle assembly protein | -0.44 | 0.000957 | 0.027954 |
| MCM5 | 506970 | minichromosome maintenance complex component 5 | -0.44 | 0.00276 | 0.044198 |
| UBXN1 | 506676 | UBX domain protein 1 | -0.44 | 0.000876 | 0.026869 |
| DNPH1 | 613560 | 2'-deoxynucleoside 5'-phosphate N-hydrolase 1 | -0.44 | 0.003226 | 0.047158 |
| NAA10 | 613636 | N-alpha-acetyltransferase 10, NatA catalytic subunit | -0.44 | 0.001613 | 0.034283 |
| GSK3A | 536561 | glycogen synthase kinase 3 alpha | -0.44 | 0.000798 | 0.025606 |
| BAZ1B | 508442 | bromodomain adjacent to zinc finger domain 1B | -0.44 | 0.001958 | 0.037853 |
| PLCB2 | 508888 | phospholipase C beta 2 | -0.44 | 0.000444 | 0.020431 |
| LOC101904947 | 101904947 | uncharacterized LOC101904947 | -0.44 | 0.000446 | 0.020431 |
| GNB2 | 281202 | G protein subunit beta 2 | -0.44 | 0.000571 | 0.022597 |
| CENPT | 513195 | centromere protein T | -0.44 | 0.000587 | 0.022759 |
| LMF1 | 505124 | lipase maturation factor 1 | -0.44 | 0.000988 | 0.028349 |
| PRR3 | 525344 | proline rich 3 | -0.44 | 0.000759 | 0.025204 |
| C1QTNF12 | 506102 | C1q and TNF related 12 | -0.44 | 0.000562 | 0.022597 |
| BSCL2 | 513558 | BSCL2 lipid droplet biosis associated, seipin | -0.43 | 0.002783 | 0.044205 |
| TRAF3 | 506182 | TNF receptor associated factor 3 | -0.43 | 0.000737 | 0.024941 |
| TRIM41 | 540265 | tripartite motif containing 41 | -0.43 | 0.002814 | 0.04434 |
| RNF187 | 618753 | ring finger protein 187 | -0.43 | 0.000528 | 0.022235 |
| C25H7orf50 | 522840 | chromosome 25 C7orf50 homolog | -0.43 | 0.000938 | 0.027707 |
| WNK1 | 506433 | WNK lysine deficient protein kinase 1 | -0.43 | 0.002129 | 0.039204 |
| SCLY | 790815 | selenocysteine lyase | -0.43 | 0.003341 | 0.047954 |
| ELL | 506578 | elongation factor for RNA polymerase II | -0.43 | 0.001765 | 0.035942 |
| PIGQ | 508048 | phosphatidylinositol glycan anchor biosynthesis class Q | -0.43 | 0.001818 | 0.036458 |
| TRAPPC9 | 533451 | trafficking protein particle complex subunit 9 | -0.43 | 0.001071 | 0.029051 |
| MYO9B | 513493 | myosin IXB | -0.43 | 0.002978 | 0.045325 |
| ST3GAL2 | 444879 | ST3 beta-galactoside alpha-2,3-sialyltransferase 2 | -0.42 | 0.001988 | 0.038124 |
| MOSPD3 | 506163 | motile sperm domain containing 3 | -0.42 | 0.001507 | 0.033269 |
| TMEM94 | 512110 | transmembrane protein 94 | -0.42 | 0.000719 | 0.024712 |
| XYLT1 | 541295 | xylosyltransferase 1 | -0.42 | 0.002756 | 0.044198 |
| COPS7A | 505079 | COP9 signalosome subunit 7A | -0.42 | 0.001496 | 0.033269 |
| SF3B2 | 531931 | splicing factor 3b subunit 2 | -0.42 | 0.001105 | 0.029169 |
| U2AF1L4 | 615198 | U2 small nuclear RNA auxiliary factor 1 like 4 | -0.42 | 0.000841 | 0.026199 |
| HIP1R | 508291 | huntingtin interacting protein 1 related | -0.42 | 0.002783 | 0.044205 |
| SPATA7 | 510352 | spermatosis associated 7 | -0.41 | 0.000356 | 0.018454 |
| PEDS1 | 507694 | plasmanylethanolamine desaturase 1 | -0.41 | 0.002433 | 0.041846 |
| PLIN3 | 767984 | perilipin 3 | -0.41 | 0.00298 | 0.045325 |
| MAD1L1 | 517837 | mitotic arrest deficient 1 like 1 | -0.41 | 0.00188 | 0.036998 |
| ACTG1 | 404122 | actin gamma 1 | -0.41 | 0.002388 | 0.041648 |
| HM13 | 512534 | histocompatibility minor 13 | -0.41 | 0.002033 | 0.038476 |
| KCNAB2 | 541597 | potassium voltage-gated channel subfamily A regulatory beta subunit 2 | -0.41 | 0.00118 | 0.030211 |
| SRSF5 | 510474 | serine and arginine rich splicing factor 5 | -0.41 | 0.002681 | 0.043608 |
| CRTC2 | 540959 | CREB regulated transcription coactivator 2 | -0.41 | 0.003203 | 0.046996 |
| CLN8 | 530874 | CLN8 transmembrane ER and ERGIC protein | -0.40 | 0.002974 | 0.045325 |
| PML | 100138545 | PML nuclear body scaffold | -0.40 | 0.002458 | 0.041935 |
| HERC3 | 510924 | HECT and RLD domain containing E3 ubiquitin protein ligase 3 | -0.40 | 0.001564 | 0.033845 |
| LOC100847190 | 100847190 | zinc finger protein 764 | -0.40 | 0.001108 | 0.029169 |
| KDM4B | 508141 | lysine demethylase 4B | -0.40 | 0.001068 | 0.029051 |
| MMP25 | 531092 | matrix metallopeptidase 25 | -0.40 | 0.003398 | 0.04835 |
| KLHL22 | 540134 | kelch like family member 22 | -0.40 | 0.001366 | 0.032633 |
| ZNF467 | 527301 | zinc finger protein 467 | -0.40 | 0.001254 | 0.031202 |
| C3H1orf122 | 132344942 | chromosome 3 C1orf122 homolog | -0.40 | 0.000223 | 0.015078 |
| MED15 | 618232 | mediator complex subunit 15 | -0.40 | 0.000301 | 0.01734 |
| POLR1G | 617937 | RNA polymerase I subunit G | -0.40 | 0.000649 | 0.023727 |
| TMEM234 | 100126052 | transmembrane protein 234 | -0.40 | 0.000158 | 0.012705 |
| METTL26 | 514636 | methyltransferase like 26 | -0.40 | 0.002331 | 0.041251 |
| PCNX3 | 536942 | pecanex 3 | -0.39 | 0.002075 | 0.03895 |
| ARAP1 | 511889 | ArfGAP with RhoGAP domain, ankyrin repeat and PH domain 1 | -0.39 | 0.00138 | 0.032633 |
| CTU1 | 522824 | cytosolic thiouridylase subunit 1 | -0.39 | 0.002341 | 0.041332 |
| SLC3A2 | 507107 | solute carrier family 3 member 2 | -0.39 | 0.002787 | 0.044223 |
| PEF1 | 506928 | penta-EF-hand domain containing 1 | -0.39 | 0.003569 | 0.049873 |
| LOC132345555 | 132345555 | uncharacterized LOC132345555 | -0.39 | 0.00057 | 0.022597 |
| C23H6orf226 | 100140540 | chromosome 23 C6orf226 homolog | -0.39 | 0.002427 | 0.041846 |
| FARS2 | 505390 | phenylalanyl-tRNA synthetase 2, mitochondrial | -0.39 | 0.003555 | 0.049756 |
| PLEKHM1 | 523424 | pleckstrin homology and RUN domain containing M1 | -0.39 | 0.001833 | 0.03657 |
| AREL1 | 539472 | apoptosis resistant E3 ubiquitin protein ligase 1 | -0.39 | 0.001164 | 0.030014 |
| SMG6 | 516411 | SMG6 nonsense mediated mRNA decay factor | -0.39 | 0.00116 | 0.029991 |
| SARS2 | 282060 | seryl-tRNA synthetase 2, mitochondrial | -0.38 | 0.0003 | 0.01734 |
| SYS1 | 614236 | SYS1 golgi trafficking protein | -0.38 | 0.00343 | 0.04866 |
| PPCDC | 614957 | phosphopantothenoylcysteine decarboxylase | -0.38 | 0.001763 | 0.035942 |
| MDH2 | 281306 | malate dehydrogenase 2 | -0.38 | 0.001567 | 0.033845 |
| IDH3G | 614145 | isocitrate dehydrogenase (NAD(+)) 3 non-catalytic subunit gamma | -0.38 | 0.001065 | 0.029051 |
| MRPS18B | 510824 | mitochondrial ribosomal protein S18B | -0.38 | 0.00032 | 0.017809 |
| SLF1 | 520250 | SMC5-SMC6 complex localization factor 1 | -0.38 | 0.001086 | 0.029051 |
| VIM | 280955 | vimentin | -0.38 | 0.001665 | 0.034985 |
| RWDD3 | 614557 | RWD domain containing 3 | -0.38 | 0.002199 | 0.039895 |
| PRR14 | 533235 | proline rich 14 | -0.37 | 0.003473 | 0.049102 |
| FBXO6 | 513023 | F-box protein 6 | -0.37 | 0.001292 | 0.031517 |
| LOC132343892 | 132343892 | uncharacterized LOC132343892 | -0.37 | 0.003288 | 0.047598 |
| GIMAP6 | 528288 | GTPase, IMAP family member 6 | -0.37 | 0.002749 | 0.044195 |
| ECE1 | 281133 | endothelin converting enzyme 1 | -0.37 | 0.002507 | 0.042281 |
| ZSWIM7 | 514094 | zinc finger SWIM-type containing 7 | -0.37 | 0.001542 | 0.03356 |
| CBX4 | 767865 | chromobox 4 | -0.37 | 0.000649 | 0.023727 |
| SLC38A5 | 512495 | solute carrier family 38 member 5 | -0.37 | 0.001744 | 0.035701 |
| LIMD2 | 508942 | LIM domain containing 2 | -0.36 | 0.001074 | 0.029051 |
| UFD1 | 507124 | ubiquitin recognition factor in ER associated degradation 1 | -0.36 | 0.000349 | 0.018269 |
| MRPL10 | 515014 | mitochondrial ribosomal protein L10 | -0.36 | 0.00209 | 0.039013 |
| ALKBH3 | 514579 | alkB homolog 3, alpha-ketoglutarate dependent dioxygenase | -0.36 | 0.000637 | 0.023578 |
| MRM1 | 526282 | mitochondrial rRNA methyltransferase 1 | -0.36 | 0.00101 | 0.02844 |
| TIMM50 | 505489 | translocase of inner mitochondrial membrane 50 | -0.36 | 0.002131 | 0.039204 |
| NR1D2 | 532076 | nuclear receptor subfamily 1 group D member 2 | -0.36 | 0.003169 | 0.04685 |
| IAH1 | 614320 | isoamyl acetate hydrolyzing esterase 1 (putative) | -0.36 | 0.00185 | 0.036665 |
| INO80C | 533426 | INO80 complex subunit C | -0.35 | 0.000694 | 0.024489 |
| AKNA | 539274 | AT-hook transcription factor | -0.35 | 0.000923 | 0.02756 |
| PIH1D1 | 509212 | PIH1 domain containing 1 | -0.35 | 0.002143 | 0.039374 |
| NOSIP | 511234 | nitric oxide synthase interacting protein | -0.35 | 0.002272 | 0.040639 |
| RBM42 | 540172 | RNA binding motif protein 42 | -0.35 | 0.000203 | 0.014411 |
| SMARCC2 | 509060 | SWI/SNF related, matrix associated, actin dependent regulator of chromatin subfamily c member 2 | -0.35 | 0.001964 | 0.037853 |
| PGLS | 616120 | 6-phosphogluconolactonase | -0.35 | 0.003107 | 0.046495 |
| ACAD9 | 617428 | acyl-CoA dehydrogenase family member 9 | -0.35 | 0.00256 | 0.042628 |
| CCDC12 | 617519 | coiled-coil domain containing 12 | -0.35 | 0.000376 | 0.018767 |
| HNRNPDL | 534770 | heteroous nuclear ribonucleoprotein D like | -0.35 | 0.002149 | 0.039445 |
| UROD | 504914 | uroporphyrinogen decarboxylase | -0.34 | 0.002416 | 0.041846 |
| QARS1 | 514586 | glutaminyl-tRNA synthetase 1 | -0.34 | 0.002118 | 0.039204 |
| TBC1D17 | 514858 | TBC1 domain family member 17 | -0.34 | 0.002206 | 0.039982 |
| MPST | 507313 | mercaptopyruvate sulfurtransferase | -0.34 | 0.001227 | 0.03097 |
| NICN1 | 614730 | nicolin 1, tubulin polyglutamylase complex subunit | -0.34 | 0.000868 | 0.026803 |
| LOC515042 | 515042 | SCAN domain containing 1-like | -0.34 | 0.001145 | 0.029769 |
| HDDC2 | 509282 | HD domain containing 2 | -0.34 | 0.003388 | 0.048328 |
| UBN1 | 525895 | ubinuclein 1 | -0.34 | 0.0018 | 0.036288 |
| TRPC4AP | 532834 | transient receptor potential cation channel subfamily C member 4 associated protein | -0.34 | 0.001578 | 0.033979 |
| TSTD3 | 782657 | thiosulfate sulfurtransferase like domain containing 3 | -0.33 | 0.000223 | 0.015078 |
| SFSWAP | 512326 | splicing factor SWAP | -0.33 | 0.002128 | 0.039204 |
| SUGP1 | 533971 | SURP and G-patch domain containing 1 | -0.33 | 0.000495 | 0.021578 |
| ZNF341 | 523204 | zinc finger protein 341 | -0.33 | 0.002316 | 0.041039 |
| CD2BP2 | 513857 | CD2 cytoplasmic tail binding protein 2 | -0.33 | 0.00088 | 0.026896 |
| ADAT3 | 132342112 | adenosine deaminase tRNA specific 3 | -0.33 | 0.001005 | 0.02844 |
| LOC101906226 | 101906226 | uncharacterized LOC101906226 | -0.32 | 0.001336 | 0.032301 |
| TCTA | 616169 | T cell leukemia translocation altered | -0.32 | 0.000875 | 0.026869 |
| CD27 | 512514 | CD27 molecule | -0.32 | 0.001697 | 0.035362 |
| COPS3 | 507932 | COP9 signalosome subunit 3 | -0.32 | 0.000678 | 0.024456 |
| TTLL3 | 531866 | tubulin tyrosine ligase like 3 | -0.32 | 0.001507 | 0.033269 |
| METTL21A | 615773 | methyltransferase 21A, HSPA lysine | -0.32 | 0.001006 | 0.02844 |
| NAXE | 404132 | NAD(P)HX epimerase | -0.32 | 0.003152 | 0.046812 |
| WRAP53 | 509631 | WD repeat containing antisense to TP53 | -0.31 | 0.000835 | 0.026175 |
| LGALS12 | 518931 | galectin 12 | -0.31 | 0.002737 | 0.044095 |
| UBA1 | 282869 | ubiquitin like modifier activating enzyme 1 | -0.31 | 0.001672 | 0.035033 |
| C16H1orf159 | 509263 | chromosome 16 C1orf159 homolog | -0.31 | 0.002898 | 0.044813 |
| RASGRP2 | 512056 | RAS guanyl releasing protein 2 | -0.31 | 0.002542 | 0.042459 |
| NDUFS3 | 287327 | NADH:ubiquinone oxidoreductase core subunit S3 | -0.30 | 0.001508 | 0.033269 |
| HNRNPA3 | 782669 | heteroous nuclear ribonucleoprotein A3 | -0.30 | 0.002072 | 0.038927 |
| MORC2 | 504634 | MORC family CW-type zinc finger 2 | -0.30 | 0.00292 | 0.044948 |
| MCM7 | 539924 | minichromosome maintenance complex component 7 | -0.30 | 0.003043 | 0.045718 |
| SLC50A1 | 520463 | solute carrier family 50 member 1 | -0.30 | 0.00097 | 0.028005 |
| IL6R | 507359 | interleukin 6 receptor | -0.29 | 0.001797 | 0.036288 |
| HP1BP3 | 510194 | heterochromatin protein 1 binding protein 3 | -0.29 | 0.000551 | 0.022597 |
| ELMO1 | 509821 | engulfment and cell motility 1 | -0.29 | 0.003277 | 0.047549 |
| SNRNP35 | 512142 | small nuclear ribonucleoprotein U11/U12 subunit 35 | -0.29 | 0.001818 | 0.036458 |
| BABAM2 | 614152 | BRISC and BRCA1 A complex member 2 | -0.28 | 0.000719 | 0.024712 |
| CHMP6 | 510596 | charged multivesicular body protein 6 | -0.28 | 0.002177 | 0.039719 |
| TP53 | 281542 | tumor protein p53 | -0.27 | 0.00109 | 0.029054 |
| MIIP | 614193 | migration and invasion inhibitory protein | -0.27 | 0.002962 | 0.045299 |
| DAP | 616066 | death associated protein | -0.25 | 0.002546 | 0.042459 |
| STK16 | 521237 | serine/threonine kinase 16 | -0.24 | 0.002741 | 0.044102 |
| HADH | 532785 | hydroxyacyl-CoA dehydrogenase | -0.24 | 0.003579 | 0.049962 |
| TRAPPC4 | 785345 | trafficking protein particle complex subunit 4 | 0.27 | 0.002402 | 0.041717 |
| ZNF394 | 522836 | zinc finger protein 394 | 0.27 | 0.001982 | 0.038073 |
| BCAS2 | 507944 | BCAS2 pre-mRNA processing factor | 0.29 | 0.003353 | 0.048001 |
| CRIPT | 617723 | CXXC repeat containing interactor of PDZ3 domain | 0.29 | 0.003147 | 0.0468 |
| RARS2 | 525894 | arginyl-tRNA synthetase 2, mitochondrial | 0.30 | 0.001924 | 0.037546 |
| UBE2V2 | 286803 | ubiquitin conjugating enzyme E2 V2 | 0.30 | 0.002236 | 0.04016 |
| TMED7 | 100125926 | transmembrane p24 trafficking protein 7 | 0.30 | 0.001701 | 0.035362 |
| ZNF32 | 512392 | zinc finger protein 32 | 0.31 | 0.000312 | 0.01779 |
| AK6 | 102216273 | adenylate kinase 6 | 0.31 | 0.002436 | 0.041846 |
| MED4 | 515299 | mediator complex subunit 4 | 0.31 | 0.002974 | 0.045325 |
| RWDD4 | 509865 | RWD domain containing 4 | 0.32 | 0.002675 | 0.043586 |
| MRPL22 | 614639 | mitochondrial ribosomal protein L22 | 0.32 | 0.001246 | 0.031105 |
| FXR1 | 536793 | FMR1 autosomal homolog 1 | 0.32 | 0.001656 | 0.034865 |
| MRPL51 | 513622 | mitochondrial ribosomal protein L51 | 0.32 | 0.001463 | 0.033269 |
| SBNO1 | 540582 | strawberry notch homolog 1 | 0.32 | 0.001368 | 0.032633 |
| ZNF207 | 505177 | zinc finger protein 207 | 0.33 | 0.002185 | 0.039797 |
| RDH14 | 505949 | retinol dehydrogenase 14 | 0.33 | 0.003259 | 0.04747 |
| RASA1 | 282032 | RAS p21 protein activator 1 | 0.33 | 0.000977 | 0.028127 |
| C3H1orf52 | 509947 | chromosome 3 C1orf52 homolog | 0.33 | 0.000943 | 0.027816 |
| CSNK2A1 | 282419 | casein kinase 2 alpha 1 | 0.34 | 0.000998 | 0.02844 |
| RHOT1 | 511257 | ras homolog family member T1 | 0.34 | 0.002401 | 0.041717 |
| TES | 534965 | testin LIM domain protein | 0.34 | 0.001797 | 0.036288 |
| ODR4 | 507724 | odr-4 GPCR localization factor homolog | 0.34 | 0.001627 | 0.034469 |
| PAIP2 | 536619 | poly(A) binding protein interacting protein 2 | 0.34 | 0.001576 | 0.033979 |
| POLR3K | 618296 | RNA polymerase III subunit K | 0.34 | 0.002516 | 0.042326 |
| CEP57 | 353245 | centrosomal protein 57 | 0.34 | 0.003268 | 0.047475 |
| RAB10 | 783373 | RAB10, member RAS onco family | 0.34 | 0.002547 | 0.042459 |
| KCTD6 | 613649 | potassium channel tetramerization domain containing 6 | 0.35 | 0.001072 | 0.029051 |
| TMEM243 | 614280 | transmembrane protein 243 | 0.35 | 0.003002 | 0.045402 |
| GIMAP4 | 510751 | GTPase, IMAP family member 4 | 0.35 | 0.000961 | 0.028005 |
| BRCC3 | 519513 | BRCA1/BRCA2-containing complex subunit 3 | 0.35 | 0.003484 | 0.049204 |
| CSE1L | 518622 | chromosome segregation 1 like | 0.35 | 0.002392 | 0.041673 |
| RPE | 533764 | ribulose-5-phosphate-3-epimerase | 0.35 | 0.002304 | 0.040909 |
| LOC522845 | 522845 | ankyrin repeat domain-containing protein 26 | 0.36 | 0.003054 | 0.045791 |
| C11H2orf49 | 540024 | chromosome 11 C2orf49 homolog | 0.36 | 0.001247 | 0.031105 |
| SSB | 338071 | small RNA binding exonuclease protection factor La | 0.36 | 0.00266 | 0.043556 |
| TXK | 504782 | TXK tyrosine kinase | 0.36 | 0.002808 | 0.04434 |
| ZZZ3 | 538498 | zinc finger ZZ-type containing 3 | 0.36 | 0.00209 | 0.039013 |
| COPS4 | 540223 | COP9 signalosome subunit 4 | 0.36 | 0.002008 | 0.038265 |
| C24H18orf25 | 615069 | ST8 alpha-N-acetyl-neuraminide alpha-2,8-sialyltransferase 5 | 0.37 | 0.002285 | 0.040748 |
| GON7 | 505131 | GON7 subunit of KEOPS complex | 0.37 | 0.001287 | 0.031446 |
| TMEM165 | 532600 | transmembrane protein 165 | 0.37 | 0.001263 | 0.031236 |
| EIF4G2 | 286870 | eukaryotic translation initiation factor 4 gamma 2 | 0.37 | 0.002505 | 0.042281 |
| MID1IP1 | 615572 | MID1 interacting protein 1 | 0.37 | 0.002903 | 0.044843 |
| FAM98A | 530070 | family with sequence similarity 98 member A | 0.37 | 0.003538 | 0.049691 |
| ZCCHC18 | 786510 | zinc finger CCHC-type containing 18 | 0.37 | 0.002536 | 0.042459 |
| POLR3F | 529410 | RNA polymerase III subunit F | 0.37 | 0.002755 | 0.044198 |
| SOCS5 | 514773 | suppressor of cytokine signaling 5 | 0.37 | 0.001248 | 0.031105 |
| ZWILCH | 534811 | zwilch kinetochore protein | 0.38 | 0.001281 | 0.031435 |
| MMADHC | 521533 | metabolism of cobalamin associated D | 0.38 | 0.002506 | 0.042281 |
| HAGHL | 511836 | hydroxyacylglutathione hydrolase like | 0.38 | 0.00267 | 0.043586 |
| SNRNP48 | 513601 | small nuclear ribonucleoprotein U11/U12 subunit 48 | 0.38 | 0.002627 | 0.043328 |
| RIOK1 | 516289 | RIO kinase 1 | 0.39 | 0.003259 | 0.04747 |
| PPP1R3E | 527187 | protein phosphatase 1 regulatory subunit 3E | 0.39 | 0.002911 | 0.044924 |
| GTF2F2 | 509259 | ral transcription factor IIF subunit 2 | 0.39 | 0.001907 | 0.037391 |
| LOC100848895 | 100848895 | zinc finger protein 665 | 0.39 | 0.001007 | 0.02844 |
| SCO1 | 508586 | synthesis of cytochrome C oxidase 1 | 0.39 | 0.00246 | 0.041935 |
| CMAH | 537017 | cytidine monophospho-N-acetylneuraminic acid hydroxylase | 0.39 | 0.000396 | 0.019296 |
| ZNF12 | 511194 | zinc finger protein 12 | 0.39 | 0.003351 | 0.048001 |
| RBM4B | 505756 | RNA binding motif protein 4B | 0.39 | 0.001052 | 0.028984 |
| NAA20 | 540212 | N-alpha-acetyltransferase 20, NatB catalytic subunit | 0.39 | 0.001711 | 0.035362 |
| MTF2 | 615211 | metal response element binding transcription factor 2 | 0.39 | 0.001927 | 0.037546 |
| LOC789027 | 789027 | putative uncharacterized transposon-derived protein F52C9.6 | 0.39 | 0.001759 | 0.035905 |
| ERGIC2 | 512481 | ERGIC and golgi 2 | 0.39 | 0.003302 | 0.047686 |
| CEP20 | 782821 | myosin heavy chain 11 | 0.40 | 0.000757 | 0.025204 |
| THOC3 | 529231 | THO complex subunit 3 | 0.40 | 0.000899 | 0.027353 |
| UQCRC2 | 282394 | ubiquinol-cytochrome c reductase core protein 2 | 0.40 | 0.001972 | 0.037953 |
| TMEM126B | 504229 | transmembrane protein 126B | 0.40 | 0.000768 | 0.025304 |
| SENP1 | 538091 | SUMO specific peptidase 1 | 0.40 | 0.001519 | 0.033308 |
| NHLRC2 | 534327 | NHL repeat containing 2 | 0.40 | 0.002111 | 0.039204 |
| TARDBP | 540632 | TAR DNA binding protein | 0.40 | 0.001109 | 0.029169 |
| SLC25A32 | 782162 | solute carrier family 25 member 32 | 0.41 | 0.001433 | 0.032916 |
| CCT4 | 613336 | chaperonin containing TCP1 subunit 4 | 0.41 | 0.003399 | 0.04835 |
| PAFAH1B2 | 282514 | platelet activating factor acetylhydrolase 1b catalytic subunit 2 | 0.41 | 0.001555 | 0.033748 |
| CLNS1A | 613968 | chloride nucleotide-sensitive channel 1A | 0.42 | 0.002188 | 0.039797 |
| ZC3H12D | 615863 | zinc finger CCCH-type containing 12D | 0.42 | 0.002027 | 0.038456 |
| COMMD8 | 507292 | COMM domain containing 8 | 0.42 | 0.000665 | 0.024163 |
| SRP14 | 512792 | signal recognition particle 14 | 0.42 | 0.000558 | 0.022597 |
| OSTC | 768033 | oligosaccharyltransferase complex non-catalytic subunit | 0.42 | 0.00019 | 0.013898 |
| CEP19 | 613916 | centrosomal protein 19 | 0.42 | 0.002123 | 0.039204 |
| SNX16 | 507462 | sorting nexin 16 | 0.42 | 0.001074 | 0.029051 |
| MTFR1L | 508852 | mitochondrial fission regulator 1 like | 0.42 | 0.003172 | 0.04685 |
| MCOLN2 | 532671 | mucolipin TRP cation channel 2 | 0.43 | 0.002701 | 0.043668 |
| DHX15 | 512327 | DEAH-box helicase 15 | 0.43 | 0.000648 | 0.023727 |
| KLHL28 | 525011 | kelch like family member 28 | 0.43 | 0.00281 | 0.04434 |
| ATP1B3 | 532844 | ATPase Na+/K+ transporting subunit beta 3 | 0.43 | 0.003021 | 0.045475 |
| TOMM70 | 507707 | translocase of outer mitochondrial membrane 70 | 0.43 | 0.000153 | 0.012705 |
| PLEKHM3 | 533312 | pleckstrin homology domain containing M3 | 0.43 | 0.002343 | 0.041332 |
| PDCD10 | 506411 | programmed cell death 10 | 0.43 | 0.000783 | 0.025525 |
| TMEM170B | 101906104 | transmembrane protein 170B | 0.44 | 0.001872 | 0.03698 |
| NCK2 | 526430 | NCK adaptor protein 2 | 0.44 | 0.000241 | 0.015798 |
| ARL4A | 767906 | ADP ribosylation factor like GTPase 4A | 0.44 | 0.001667 | 0.034985 |
| PAPOLG | 529071 | poly(A) polymerase gamma | 0.44 | 0.000313 | 0.017802 |
| FANCM | 513626 | FA complementation group M | 0.44 | 0.001141 | 0.029696 |
| MOB4 | 781884 | MOB family member 4, phocein | 0.44 | 0.002845 | 0.044539 |
| RPP14 | 515208 | hydroxyacyl-thioester dehydratase type 2 | 0.45 | 0.001912 | 0.037444 |
| MSH2 | 533115 | mutS homolog 2 | 0.45 | 0.002351 | 0.041352 |
| FDFT1 | 281767 | farnesyl-diphosphate farnesyltransferase 1 | 0.45 | 0.002685 | 0.043608 |
| MKKS | 614288 | MKKS centrosomal shuttling protein | 0.45 | 0.0016 | 0.034211 |
| RNF8 | 515933 | ring finger protein 8 | 0.45 | 0.001538 | 0.033533 |
| LCORL | 540095 | ligand dependent nuclear receptor corepressor like | 0.45 | 0.000623 | 0.023341 |
| HSPA14 | 534751 | heat shock protein family A (Hsp70) member 14 | 0.45 | 0.001362 | 0.032633 |
| ZNF239 | 100848535 | zinc finger protein 239 | 0.45 | 0.000698 | 0.024489 |
| AHSA1 | 539220 | activator of HSP90 ATPase activity 1 | 0.45 | 0.00139 | 0.032712 |
| THNSL1 | 788561 | threonine synthase like 1 | 0.45 | 0.002702 | 0.043668 |
| EAF2 | 613523 | ELL associated factor 2 | 0.45 | 0.002276 | 0.04064 |
| VPS45 | 541230 | vacuolar protein sorting 45 homolog | 0.45 | 0.003231 | 0.047195 |
| NDUFAF1 | 541232 | NADH:ubiquinone oxidoreductase complex assembly factor 1 | 0.45 | 9.47E-05 | 0.009785 |
| ETFBKMT | 530461 | electron transfer flavoprotein subunit beta lysine methyltransferase | 0.45 | 0.000692 | 0.024489 |
| DONSON | 522248 | DNA replication fork stabilization factor DONSON | 0.46 | 0.002873 | 0.044702 |
| ZKSCAN7 | 539552 | zinc finger with KRAB and SCAN domains 7 | 0.46 | 0.002506 | 0.042281 |
| UNC50 | 513629 | unc-50 inner nuclear membrane RNA binding protein | 0.46 | 0.000583 | 0.022759 |
| ETF1 | 541077 | eukaryotic translation termination factor 1 | 0.46 | 0.000154 | 0.012705 |
| TADA1 | 521918 | transcriptional adaptor 1 | 0.46 | 0.000808 | 0.025736 |
| STAT4 | 515988 | signal transducer and activator of transcription 4 | 0.46 | 0.001006 | 0.02844 |
| PRMT9 | 532021 | protein arginine methyltransferase 9 | 0.46 | 0.00179 | 0.036288 |
| KBTBD8 | 530516 | kelch repeat and BTB domain containing 8 | 0.46 | 0.003053 | 0.045791 |
| ADPRM | 534038 | ADP-ribose/CDP-alcohol diphosphatase, manganese dependent | 0.46 | 0.00044 | 0.020431 |
| MAK16 | 504683 | MAK16 homolog | 0.46 | 0.001453 | 0.033238 |
| ABHD3 | 539795 | abhydrolase domain containing 3, phospholipase | 0.47 | 0.001506 | 0.033269 |
| TRAF6 | 539124 | TNF receptor associated factor 6 | 0.47 | 0.003015 | 0.045473 |
| DEGS1 | 507290 | delta 4-desaturase, sphingolipid 1 | 0.47 | 0.001465 | 0.033269 |
| ZNF189 | 540816 | zinc finger protein 189 | 0.47 | 0.00149 | 0.033269 |
| SPR | 533836 | sepiapterin reductase | 0.47 | 0.003439 | 0.048742 |
| DPP4 | 281122 | dipeptidyl peptidase 4 | 0.47 | 0.002833 | 0.044502 |
| SS18L1 | 768207 | SS18L1 subunit of BAF chromatin remodeling complex | 0.47 | 0.003345 | 0.047973 |
| UQCC1 | 618503 | ubiquinol-cytochrome c reductase complex assembly factor 1 | 0.47 | 8.06E-05 | 0.009646 |
| RNF139 | 788471 | ring finger protein 139 | 0.48 | 0.001474 | 0.033269 |
| AAGAB | 507035 | alpha and gamma adaptin binding protein | 0.48 | 0.001376 | 0.032633 |
| SLC35A1 | 536838 | solute carrier family 35 member A1 | 0.48 | 0.001044 | 0.028926 |
| NAP1L5 | 508508 | nucleosome assembly protein 1 like 5 | 0.48 | 0.003016 | 0.045473 |
| STAP1 | 507296 | signal transducing adaptor family member 1 | 0.48 | 0.00144 | 0.033019 |
| KANSL2 | 540194 | KAT8 regulatory NSL complex subunit 2 | 0.48 | 0.002159 | 0.039529 |
| PPP4R3B | 516489 | protein phosphatase 4 regulatory subunit 3B | 0.48 | 0.001476 | 0.033269 |
| CACYBP | 618428 | calcyclin binding protein | 0.48 | 0.001499 | 0.033269 |
| ATP2B1 | 282641 | ATPase plasma membrane Ca2+ transporting 1 | 0.48 | 0.002613 | 0.043233 |
| ZNF814 | 613566 | zinc finger protein 814 | 0.48 | 0.001588 | 0.034099 |
| CD40 | 286849 | CD40 molecule | 0.48 | 0.00025 | 0.01586 |
| BOD1 | 540063 | biorientation of chromosomes in cell division 1 | 0.49 | 0.00076 | 0.025204 |
| C3H1orf109 | 508021 | AFG2 interacting ribosome maturation factor | 0.49 | 0.001379 | 0.032633 |
| ARL1 | 517345 | ADP ribosylation factor like GTPase 1 | 0.49 | 0.002372 | 0.041459 |
| CENPL | 615527 | centromere protein L | 0.49 | 0.003107 | 0.046495 |
| VPS50 | 535248 | VPS50 subunit of EARP/GARPII complex | 0.49 | 0.001073 | 0.029051 |
| NDST2 | 286838 | N-deacetylase and N-sulfotransferase 2 | 0.49 | 0.000914 | 0.027508 |
| GPR137B | 613826 | G protein-coupled receptor 137B | 0.49 | 0.001178 | 0.030211 |
| ORC6 | 515476 | origin recognition complex subunit 6 | 0.49 | 0.000621 | 0.023341 |
| RPF1 | 513081 | ribosome production factor 1 homolog | 0.49 | 0.000554 | 0.022597 |
| ACOT13 | 504870 | acyl-CoA thioesterase 13 | 0.49 | 0.002807 | 0.04434 |
| ZDHHC21 | 535814 | zinc finger DHHC-type palmitoyltransferase 21 | 0.49 | 0.000172 | 0.013096 |
| ZNF93 | 510078 | zinc finger protein 93 | 0.50 | 0.00123 | 0.03097 |
| ANLN | 518274 | anillin, actin binding protein | 0.50 | 0.002588 | 0.042912 |
| PPID | 281420 | peptidylprolyl isomerase D | 0.50 | 0.000554 | 0.022597 |
| CISD2 | 781260 | CDGSH iron sulfur domain 2 | 0.50 | 0.001453 | 0.033238 |
| CHAC2 | 511605 | ChaC glutathione specific gamma-glutamylcyclotransferase 2 | 0.51 | 0.000469 | 0.020906 |
| TLR10 | 539791 | toll like receptor 10 | 0.51 | 0.002444 | 0.041846 |
| NOCT | 540641 | nocturnin | 0.51 | 0.000204 | 0.014411 |
| FBXL20 | 511007 | F-box and leucine rich repeat protein 20 | 0.51 | 0.000434 | 0.020428 |
| RHOG2 | 531038 | RHOG family member 2 | 0.51 | 4.69E-05 | 0.007964 |
| PAIP1 | 510246 | poly(A) binding protein interacting protein 1 | 0.51 | 0.000733 | 0.024941 |
| MTMR2 | 536810 | myotubularin related protein 2 | 0.51 | 0.001065 | 0.029051 |
| MSANTD4 | 511226 | Myb/SANT DNA binding domain containing 4 with coiled-coils | 0.51 | 0.002675 | 0.043586 |
| LINS1 | 539267 | lines homolog 1 | 0.51 | 0.002705 | 0.043668 |
| LOC613444 | 613444 | RIKEN cDNA 2700097O09Rik-like | 0.51 | 0.001597 | 0.034211 |
| TMEM260 | 614796 | transmembrane protein 260 | 0.52 | 0.000938 | 0.027707 |
| TAF8 | 539938 | TATA-box binding protein associated factor 8 | 0.52 | 0.001849 | 0.036665 |
| LYSMD2 | 511013 | LysM domain containing 2 | 0.52 | 0.00011 | 0.010685 |
| COX15 | 517811 | cytochrome c oxidase assembly homolog COX15 | 0.52 | 0.000496 | 0.021578 |
| STARD4 | 132342078 | StAR related lipid transfer domain containing 4 | 0.52 | 0.000709 | 0.024626 |
| STARD4 | 100847750 | StAR related lipid transfer domain containing 4 | 0.52 | 0.000709 | 0.024626 |
| CYP51A1 | 505060 | cytochrome P450 family 51 subfamily A member 1 | 0.52 | 0.001409 | 0.032903 |
| PLEKHA5 | 532887 | pleckstrin homology domain containing A5 | 0.52 | 0.002117 | 0.039204 |
| KCTD21 | 539645 | potassium channel tetramerization domain containing 21 | 0.52 | 0.003332 | 0.047914 |
| ZFP28 | 100301148 | ZFP28 zinc finger protein | 0.53 | 0.000772 | 0.025304 |
| LOC100848357 | 100848357 | uncharacterized LOC100848357 | 0.53 | 0.001376 | 0.032633 |
| UFSP2 | 617788 | UFM1 specific peptidase 2 | 0.53 | 1.89E-05 | 0.005289 |
| CDK7 | 515462 | cyclin dependent kinase 7 | 0.53 | 0.003504 | 0.04936 |
| ZNF473 | 784785 | zinc finger protein 473 | 0.53 | 0.001793 | 0.036288 |
| PBK | 534781 | PDZ binding kinase | 0.53 | 0.0025 | 0.042281 |
| ABCE1 | 514991 | ATP binding cassette subfamily E member 1 | 0.53 | 0.000631 | 0.023497 |
| POT1 | 511292 | protection of telomeres 1 | 0.53 | 0.001875 | 0.036987 |
| GLO1 | 540335 | glyoxalase I | 0.53 | 0.000558 | 0.022597 |
| GNL3 | 506152 | G protein nucleolar 3 | 0.53 | 0.002159 | 0.039529 |
| MBNL2 | 527679 | muscleblind like splicing regulator 2 | 0.53 | 0.001774 | 0.036076 |
| FICD | 505923 | FIC domain protein adenylyltransferase | 0.54 | 0.000902 | 0.027353 |
| SNX2 | 509769 | sorting nexin 2 | 0.54 | 0.001932 | 0.037546 |
| RYK | 781353 | receptor like tyrosine kinase | 0.54 | 0.000298 | 0.017325 |
| HMGCS1 | 407767 | 3-hydroxy-3-methylglutaryl-CoA synthase 1 | 0.54 | 0.001848 | 0.036665 |
| ZNF260 | 112442192 | zinc finger protein 260 | 0.54 | 0.003269 | 0.047475 |
| PCNX4 | 539495 | pecanex 4 | 0.54 | 0.000492 | 0.021578 |
| GLT8D1 | 512192 | glycosyltransferase 8 domain containing 1 | 0.54 | 0.000463 | 0.02077 |
| GMFB | 615255 | glia maturation factor beta | 0.54 | 0.002648 | 0.043503 |
| STIP1 | 617109 | stress induced phosphoprotein 1 | 0.54 | 0.000563 | 0.022597 |
| ACTR6 | 613596 | actin related protein 6 | 0.55 | 0.002893 | 0.044789 |
| RINT1 | 510686 | RAD50 interactor 1 | 0.55 | 0.001583 | 0.034045 |
| UQCRB | 616871 | ubiquinol-cytochrome c reductase binding protein | 0.55 | 0.000762 | 0.025204 |
| MED6 | 505293 | mediator complex subunit 6 | 0.55 | 9.29E-05 | 0.009785 |
| FKRP | 539701 | fukutin related protein | 0.55 | 0.00032 | 0.017809 |
| ZNF770 | 786537 | zinc finger protein 770 | 0.55 | 0.001362 | 0.032633 |
| METTL3 | 540339 | methyltransferase 3, N6-adenosine-methyltransferase complex catalytic subunit | 0.55 | 9.12E-05 | 0.009785 |
| GDAP2 | 508774 | ganglioside induced differentiation associated protein 2 | 0.55 | 0.00038 | 0.018767 |
| GCNT2 | 613924 | glucosaminyl (N-acetyl) transferase 2, I-branching enzyme (I blood group) | 0.55 | 0.003388 | 0.048328 |
| ZNF226 | 789006 | zinc finger protein 226 | 0.56 | 0.000922 | 0.02756 |
| ZNF33B | 520684 | zinc finger protein 33B | 0.56 | 0.002668 | 0.043586 |
| TTC9C | 786577 | tetratricopeptide repeat domain 9C | 0.56 | 0.002106 | 0.039204 |
| TSPAN6 | 514741 | tetraspanin 6 | 0.56 | 7.38E-05 | 0.009586 |
| RTCB | 525106 | RNA 2',3'-cyclic phosphate and 5'-OH ligase | 0.56 | 0.002921 | 0.044948 |
| GNPNAT1 | 512299 | glucosamine-phosphate N-acetyltransferase 1 | 0.56 | 0.00037 | 0.018767 |
| ANKRD40 | 522429 | ankyrin repeat domain 40 | 0.56 | 0.001505 | 0.033269 |
| PRKRA | 282875 | protein activator of interferon induced protein kinase EIF2AK2 | 0.57 | 0.003307 | 0.047686 |
| EXOC8 | 540237 | exocyst complex component 8 | 0.57 | 0.000633 | 0.023497 |
| EPB41L4B | 519310 | erythrocyte membrane protein band 4.1 like 4B | 0.57 | 0.000444 | 0.020431 |
| ZNF181 | 767826 | zinc finger protein 181 | 0.58 | 0.00288 | 0.044702 |
| HACD4 | 618814 | 3-hydroxyacyl-CoA dehydratase 4 | 0.58 | 0.001368 | 0.032633 |
| LOC112443000 | 112443000 | uncharacterized LOC112443000 | 0.58 | 0.001054 | 0.028984 |
| SINHCAF | 538649 | SIN3-HDAC complex associated factor | 0.58 | 0.001043 | 0.028926 |
| LOC104969378 | 104969378 | uncharacterized LOC104969378 | 0.58 | 0.001035 | 0.028795 |
| PRNP | 281427 | prion protein | 0.58 | 0.001799 | 0.036288 |
| HSF2 | 510557 | heat shock transcription factor 2 | 0.58 | 0.00193 | 0.037546 |
| SLC41A2 | 524417 | solute carrier family 41 member 2 | 0.58 | 0.001416 | 0.032903 |
| LOC100299712 | 100299712 | zinc finger protein 665 | 0.58 | 8.78E-05 | 0.009785 |
| LOC104976020 | 104976020 | endogenous retrovirus group K member 9 Gag polyprotein | 0.58 | 0.003267 | 0.047475 |
| EED | 404183 | embryonic ectoderm development | 0.58 | 0.000108 | 0.010616 |
| CCT6A | 521540 | chaperonin containing TCP1 subunit 6A | 0.58 | 0.001083 | 0.029051 |
| DNAL1 | 538164 | dynein axonemal light chain 1 | 0.58 | 0.002444 | 0.041846 |
| ZBTB43 | 509462 | zinc finger and BTB domain containing 43 | 0.59 | 0.00061 | 0.023023 |
| HAUS2 | 508698 | HAUS augmin like complex subunit 2 | 0.59 | 0.00146 | 0.033269 |
| SH2D1B | 617485 | SH2 domain containing 1B | 0.59 | 0.000781 | 0.025505 |
| MOB3C | 614275 | MOB kinase activator 3C | 0.59 | 0.002577 | 0.042788 |
| EFCAB7 | 505160 | EF-hand calcium binding domain 7 | 0.60 | 0.000204 | 0.014411 |
| CERS6 | 616901 | ceramide synthase 6 | 0.60 | 0.002958 | 0.045299 |
| ERI1 | 540728 | exoribonuclease 1 | 0.60 | 0.001373 | 0.032633 |
| CCT8 | 281047 | chaperonin containing TCP1 subunit 8 | 0.60 | 0.002225 | 0.040116 |
| KIF3A | 541246 | kinesin family member 3A | 0.60 | 0.00028 | 0.016662 |
| SIMC1 | 100336733 | SUMO interacting motifs containing 1 | 0.60 | 0.0011 | 0.029165 |
| PRORP | 532995 | protein only RNase P catalytic subunit | 0.60 | 0.001497 | 0.033269 |
| CTNNAL1 | 515749 | catenin alpha like 1 | 0.60 | 0.002419 | 0.041846 |
| ZFP30 | 100124429 | ZFP30 zinc finger protein | 0.60 | 0.002652 | 0.04352 |
| BCDIN3D | 505650 | BCDIN3 domain containing RNA methyltransferase | 0.60 | 0.000993 | 0.02844 |
| ZNF484 | 538489 | zinc finger protein 484 | 0.61 | 0.000956 | 0.027954 |
| LOC112441500 | 112441500 | zinc finger protein 677 | 0.61 | 0.002397 | 0.041717 |
| LOC789996 | 789996 | zinc finger protein 211 | 0.61 | 0.001735 | 0.035555 |
| LOC790009 | 790009 | zinc finger protein 211-like | 0.61 | 0.000173 | 0.013137 |
| ELOVL5 | 617293 | ELOVL fatty acid elongase 5 | 0.61 | 0.000623 | 0.023341 |
| KYNU | 520327 | kynureninase | 0.61 | 0.001025 | 0.028732 |
| NANP | 516539 | N-acetylneuraminic acid phosphatase | 0.62 | 0.002518 | 0.042326 |
| SC5D | 525154 | sterol-C5-desaturase | 0.62 | 0.002443 | 0.041846 |
| LOC100847495 | 100847495 | uncharacterized LOC100847495 | 0.62 | 0.000512 | 0.021952 |
| C29H11orf54 | 530784 | chromosome 29 C11orf54 homolog | 0.63 | 0.000838 | 0.026175 |
| PCBD1 | 530736 | pterin-4 alpha-carbinolamine dehydratase 1 | 0.63 | 0.000572 | 0.022602 |
| PDHA1 | 407109 | pyruvate dehydrogenase E1 subunit alpha 1 | 0.63 | 0.001916 | 0.037481 |
| BNIP2 | 516077 | BCL2 interacting protein 2 | 0.63 | 0.00033 | 0.017935 |
| ZNG1A | 100300568 | Zn regulated GTPase metalloprotein activator 1 | 0.63 | 0.000322 | 0.017809 |
| KCTD10 | 540881 | potassium channel tetramerization domain containing 10 | 0.63 | 0.000179 | 0.013421 |
| LOC101902301 | 101902301 | heterogeneous nuclear ribonucleoprotein A3-like | 0.63 | 0.00195 | 0.037813 |
| AHSA2 | 531017 | activator of HSP90 ATPase homolog 2 | 0.63 | 0.001953 | 0.037832 |
| MTX2 | 615424 | metaxin 2 | 0.63 | 0.000599 | 0.022803 |
| ZNF677 | 525522 | zinc finger protein 677 | 0.63 | 0.00012 | 0.011306 |
| ORC5 | 519409 | origin recognition complex subunit 5 | 0.63 | 0.001099 | 0.029165 |
| LOC619000 | 619000 | ankyrin repeat domain-containing protein 26 | 0.63 | 0.001133 | 0.029534 |
| TMEM19 | 538974 | transmembrane protein 19 | 0.64 | 0.001213 | 0.030959 |
| P4HA1 | 518288 | prolyl 4-hydroxylase subunit alpha 1 | 0.64 | 0.000217 | 0.01499 |
| MSMO1 | 504481 | methylsterol monooxygenase 1 | 0.64 | 0.00186 | 0.036783 |
| IFT57 | 531436 | intraflagellar transport 57 | 0.64 | 1.61E-05 | 0.004992 |
| RXYLT1 | 515147 | ribitol xylosyltransferase 1 | 0.65 | 0.002684 | 0.043608 |
| LOC504548 | 504548 | ubiquitin D | 0.65 | 0.001751 | 0.035805 |
| ANKMY2 | 509032 | ankyrin repeat and MYND domain containing 2 | 0.65 | 7.78E-05 | 0.009606 |
| GPR171 | 767929 | G protein-coupled receptor 171 | 0.65 | 0.000198 | 0.014185 |
| TAF13 | 513065 | TATA-box binding protein associated factor 13 | 0.65 | 0.001263 | 0.031236 |
| SPIN2 | 506451 | spindlin family, member 2 | 0.65 | 0.002245 | 0.040262 |
| FSD1L | 510535 | fibronectin type III and SPRY domain containing 1 like | 0.66 | 5.25E-05 | 0.008378 |
| ZNF596 | 511959 | zinc finger protein 596 | 0.66 | 0.00024 | 0.015798 |
| NANOS1 | 786356 | nanos C2HC-type zinc finger 1 | 0.66 | 0.001048 | 0.028962 |
| KLHDC2 | 535436 | kelch domain containing 2 | 0.66 | 0.000132 | 0.011918 |
| ZNF461 | 100296502 | zinc finger protein 461 | 0.66 | 0.001107 | 0.029169 |
| RAD54B | 533414 | RAD54 homolog B | 0.66 | 0.000374 | 0.018767 |
| LOC100140915 | 100140915 | zinc finger protein OZF | 0.66 | 0.001408 | 0.032903 |
| KIF18A | 537566 | kinesin family member 18A | 0.67 | 0.000985 | 0.028292 |
| LOC507787 | 507787 | multiple coagulation factor deficiency protein 2 homolog | 0.67 | 0.000632 | 0.023497 |
| NCBP1 | 100124428 | nuclear cap binding protein subunit 1 | 0.67 | 0.000247 | 0.01586 |
| LOC101907749 | 101907749 | uncharacterized LOC101907749 | 0.67 | 0.001568 | 0.033845 |
| NMNAT1 | 522863 | nicotinamide nucleotide adenylyltransferase 1 | 0.68 | 0.000726 | 0.02488 |
| ZNF729 | 768043 | zinc finger protein 729 | 0.68 | 0.0017 | 0.035362 |
| ZNF383 | 112442193 | zinc finger protein 383 | 0.68 | 0.000499 | 0.021578 |
| TMEM184C | 504966 | transmembrane protein 184C | 0.68 | 0.002481 | 0.042106 |
| CYP2R1 | 541302 | cytochrome P450 family 2 subfamily R member 1 | 0.68 | 1.69E-05 | 0.004992 |
| TRIM36 | 539023 | tripartite motif containing 36 | 0.68 | 0.000111 | 0.010689 |
| SLC49A4 | 783019 | solute carrier family 49 member 4 | 0.68 | 0.001468 | 0.033269 |
| BANP | 513446 | BTG3 associated nuclear protein | 0.69 | 0.000308 | 0.017717 |
| DCLRE1C | 517886 | DNA cross-link repair 1C | 0.69 | 0.000228 | 0.015293 |
| C16H1orf74 | 511599 | chromosome 16 C1orf74 homolog | 0.69 | 0.001116 | 0.029214 |
| C1GALT1 | 539417 | core 1 synthase, glycoprotein-N-acetylgalactosamine 3-beta-galactosyltransferase 1 | 0.69 | 0.000907 | 0.027394 |
| BTLA | 531767 | B and T lymphocyte associated | 0.69 | 0.001512 | 0.033269 |
| LOC132345896 | 132345896 | uncharacterized LOC132345896 | 0.69 | 0.001486 | 0.033269 |
| ABRAXAS1 | 504796 | abraxas 1, BRCA1 A complex subunit | 0.69 | 6.17E-06 | 0.003433 |
| LOC787397 | 787397 | zinc finger protein 607 | 0.70 | 0.001327 | 0.032259 |
| IPPK | 521083 | inositol-pentakisphosphate 2-kinase | 0.70 | 0.00107 | 0.029051 |
| LOC112443328 | 112443328 | Small nucleolar RNA SNORA3/SNORA45 family | 0.70 | 0.000966 | 0.028005 |
| NCR3LG1 | 523303 | natural killer cell cytotoxicity receptor 3 ligand 1 | 0.70 | 0.002005 | 0.038265 |
| LOC787287 | 787287 | zinc finger protein 471 | 0.70 | 0.002017 | 0.038362 |
| WDR47 | 512354 | WD repeat domain 47 | 0.70 | 0.00292 | 0.044948 |
| WDR89 | 539045 | WD repeat domain 89 | 0.71 | 0.001391 | 0.032712 |
| LOC132342469 | 132342469 | uncharacterized LOC132342469 | 0.71 | 0.003284 | 0.047598 |
| LOC786726 | 786726 | heterogeneous nuclear ribonucleoprotein K-like | 0.71 | 0.000719 | 0.024712 |
| E2F5 | 539427 | E2F transcription factor 5 | 0.71 | 0.000397 | 0.019296 |
| BTG3 | 541054 | BTG anti-proliferation factor 3 | 0.71 | 0.001419 | 0.032903 |
| LOC101909736 | 101909736 | uncharacterized LOC101909736 | 0.72 | 6.74E-05 | 0.009576 |
| NOCT | 540641 | nocturnin | 0.72 | 1.58E-05 | 0.049982 |
| TCP1 | 512043 | t-complex 1 | 0.72 | 0.000356 | 0.018454 |
| MARS2 | 514726 | methionyl-tRNA synthetase 2, mitochondrial | 0.72 | 5.67E-05 | 0.008663 |
| ARL8B | 511009 | ADP ribosylation factor like GTPase 8B | 0.72 | 0.000351 | 0.018289 |
| ZNF829 | 790881 | zinc finger protein 829 | 0.72 | 9.27E-05 | 0.009785 |
| FAM89A | 616421 | family with sequence similarity 89 member A | 0.73 | 0.001374 | 0.032633 |
| LOC112442563 | 112442563 | uncharacterized LOC112442563 | 0.73 | 0.00151 | 0.033269 |
| RFXAP | 540760 | regulatory factor X associated protein | 0.73 | 0.001086 | 0.029051 |
| CFL2 | 539332 | cofilin 2 | 0.73 | 0.000128 | 0.011873 |
| ZNF892 | 132342086 | zinc finger protein 892 | 0.73 | 0.001337 | 0.032301 |
| CTH | 539159 | cystathionine gamma-lyase | 0.73 | 0.003188 | 0.046996 |
| PHF6 | 518186 | PHD finger protein 6 | 0.73 | 0.000135 | 0.012038 |
| SLC19A2 | 532860 | solute carrier family 19 member 2 | 0.74 | 0.00128 | 0.031435 |
| BAG2 | 506107 | BAG cochaperone 2 | 0.75 | 0.000269 | 0.016399 |
| LOC107132982 | 107132982 | uncharacterized LOC107132982 | 0.76 | 0.000217 | 0.01499 |
| CHORDC1 | 505144 | cysteine and histidine rich domain containing 1 | 0.76 | 0.000136 | 0.012038 |
| OBI1 | 782050 | ORC ubiquitin ligase 1 | 0.77 | 0.001298 | 0.031617 |
| TNFAIP1 | 539674 | TNF alpha induced protein 1 | 0.77 | 0.003196 | 0.046996 |
| LOC132342570 | 132342570 | uncharacterized LOC132342570 | 0.77 | 0.002816 | 0.04434 |
| ARL6 | 519014 | ADP ribosylation factor like GTPase 6 | 0.78 | 0.000349 | 0.018269 |
| LOC101907622 | 101907622 | ADP-ribosyl cyclase/cyclic ADP-ribose hydrolase 1-like | 0.78 | 0.001212 | 0.030959 |
| C9orf72 | 506309 | C9orf72-SMCR8 complex subunit | 0.79 | 0.00038 | 0.018767 |
| LOC510185 | 510185 | interleukin-2 receptor subunit beta | 0.80 | 0.000687 | 0.024487 |
| LOC104969670 | 104969670 | uncharacterized LOC104969670 | 0.80 | 0.002348 | 0.041352 |
| ZNF565 | 538504 | zinc finger protein 565 | 0.80 | 0.001499 | 0.033269 |
| XRCC2 | 789410 | X-ray repair cross complementing 2 | 0.82 | 0.000338 | 0.018151 |
| PVR | 526865 | PVR cell adhesion molecule | 0.82 | 0.002514 | 0.042326 |
| CLK1 | 613808 | CDC like kinase 1 | 0.83 | 0.001013 | 0.028474 |
| SCIN | 281478 | scinderin | 0.84 | 0.001231 | 0.03097 |
| LOC112446726 | 112446726 | apolipoprotein L3 | 0.85 | 5.12E-05 | 0.008378 |
| CPM | 513281 | carboxypeptidase M | 0.85 | 0.001391 | 0.032712 |
| SVOPL | 518832 | ATPase H+ transporting V0 subunit a4 | 0.85 | 0.002924 | 0.044948 |
| FASTKD5 | 788680 | FAST kinase domains 5 | 0.85 | 0.002881 | 0.044702 |
| DNAJA1 | 528862 | DnaJ heat shock protein family (Hsp40) member A1 | 0.86 | 0.002368 | 0.041437 |
| LOC132346917 | 132346917 | uncharacterized LOC132346917 | 0.86 | 0.000585 | 0.022759 |
| HSPA8 | 281831 | heat shock protein family A (Hsp70) member 8 | 0.86 | 6.81E-05 | 0.009576 |
| ADAP2 | 534106 | ArfGAP with dual PH domains 2 | 0.86 | 0.002305 | 0.040909 |
| LOC786798 | 786798 | bile acid-CoA:amino acid N-acyltransferase | 0.86 | 0.003495 | 0.049308 |
| LOC112448903 | 112448903 | Small nucleolar RNA SNORD36 | 0.87 | 0.002847 | 0.044539 |
| STIP1 | 617109 | stress induced phosphoprotein 1 | 0.89 | 1.1E-05 | 0.043531 |
| IL17RE | 783335 | interleukin 17 receptor E | 0.89 | 0.001429 | 0.032903 |
| FBXO48 | 786890 | F-box protein 48 | 0.90 | 0.000916 | 0.027508 |
| TBC1D8B | 100337282 | TBC1 domain family member 8B | 0.91 | 0.000268 | 0.016387 |
| GPR82 | 101904496 | G protein-coupled receptor 82 | 0.91 | 0.003196 | 0.046996 |
| MEMO1 | 615803 | mediator of cell motility 1 | 0.91 | 0.001015 | 0.028493 |
| LOC104976009 | 104976009 | uncharacterized LOC104976009 | 0.91 | 6.18E-06 | 0.003433 |
| DNAJB9 | 614588 | DnaJ heat shock protein family (Hsp40) member B9 | 0.92 | 0.000595 | 0.022771 |
| ANKRD37 | 509139 | ankyrin repeat domain 37 | 0.92 | 0.000131 | 0.011906 |
| ELOVL4 | 532015 | ELOVL fatty acid elongase 4 | 0.93 | 0.000739 | 0.024945 |
| C18H16orf46 | 614002 | chromosome 18 C16orf46 homolog | 0.94 | 0.00023 | 0.015298 |
| TNFSF15 | 514239 | TNF superfamily member 15 | 0.95 | 0.00071 | 0.024626 |
| NUDT13 | 504993 | nudix hydrolase 13 | 0.96 | 0.000462 | 0.02077 |
| XKRX | 524975 | XK related X-linked | 0.96 | 7.7E-05 | 0.009586 |
| KCNE3 | 527762 | potassium voltage-gated channel subfamily E regulatory subunit 3 | 0.96 | 0.000438 | 0.020431 |
| ADRB2 | 281605 | adrenoceptor beta 2 | 0.96 | 0.000191 | 0.013898 |
| MIS12 | 767858 | MIS12 kinetochore complex component | 0.97 | 4.86E-05 | 0.008175 |
| HYKK | 530270 | hydroxylysine kinase | 0.97 | 0.000588 | 0.022759 |
| TASL | 513911 | TLR adaptor interacting with endolysosomal SLC15A4 | 0.98 | 5.97E-05 | 0.008897 |
| LOC789148 | 789148 | RAP1B, member of RAS onco family | 0.98 | 0.000344 | 0.018179 |
| GRHL1 | 617248 | grainyhead like transcription factor 1 | 0.99 | 0.001993 | 0.03818 |
| LOC132344835 | 132344835 | uncharacterized LOC132344835 | 1.00 | 0.000103 | 0.010351 |
| ZNF165 | 506398 | zinc finger protein 165 | 1.00 | 0.000131 | 0.011906 |
| KLF15 | 407241 | KLF transcription factor 15 | 1.02 | 0.003369 | 0.048144 |
| RSRP1 | 615263 | arginine and serine rich protein 1 | 1.02 | 8.15E-05 | 0.009686 |
| OTUB2 | 504880 | OTU deubiquitinase, ubiquitin aldehyde binding 2 | 1.02 | 0.001262 | 0.031236 |
| GPR141 | 538837 | G protein-coupled receptor 141 | 1.03 | 3.51E-05 | 0.00688 |
| SCML1 | 786670 | Scm polycomb group protein like 1 | 1.03 | 0.002188 | 0.039797 |
| CLECL1 | 618591 | C-type lectin like 1 | 1.04 | 0.000688 | 0.024487 |
| RPGRIP1L | 518659 | RPGRIP1 like | 1.04 | 1.65E-05 | 0.004992 |
| RGMB | 540954 | repulsive guidance molecule BMP co-receptor b | 1.05 | 0.000513 | 0.021952 |
| ABAT | 280969 | 4-aminobutyrate aminotransferase | 1.05 | 0.001076 | 0.029051 |
| HORMAD1 | 529615 | HORMA domain containing 1 | 1.06 | 0.001286 | 0.031446 |
| TMEM100 | 613987 | transmembrane protein 100 | 1.07 | 0.001428 | 0.032903 |
| TCAM1 | 616168 | testicular cell adhesion molecule 1 | 1.10 | 0.00079 | 0.025542 |
| GPR148 | 787234 | G protein-coupled receptor 148 | 1.11 | 0.000633 | 0.023497 |
| ART1 | 539042 | ADP-ribosyltransferase 1 | 1.11 | 0.000565 | 0.022597 |
| LOC132342863 | 132342863 | small ribosomal subunit protein eS10-like | 1.11 | 0.001008 | 0.02844 |
| FCER1A | 506783 | Fc epsilon receptor Ia | 1.12 | 0.001086 | 0.029051 |
| SOCS1 | 518795 | suppressor of cytokine signaling 1 | 1.13 | 0.001411 | 0.032903 |
| STXBP6 | 534718 | syntaxin binding protein 6 | 1.15 | 0.001334 | 0.032301 |
| OSTN | 511114 | osteocrin | 1.16 | 0.000705 | 0.024626 |
| LOC132344119 | 132344119 | uncharacterized LOC132344119 | 1.19 | 0.00123 | 0.03097 |
| ROR2 | 785924 | receptor tyrosine kinase like orphan receptor 2 | 1.20 | 0.002632 | 0.043333 |
| LOC782951 | 782951 | dnaJ homolog subfamily A member 1-like | 1.25 | 4.37E-05 | 0.007586 |
| ARSJ | 540514 | arylsulfatase family member J | 1.26 | 0.001712 | 0.035362 |
| LOC112448867 | 112448867 | uncharacterized LOC112448867 | 1.33 | 0.002999 | 0.045397 |
| SPRY1 | 507095 | sprouty RTK signaling antagonist 1 | 1.33 | 6.18E-05 | 0.00904 |
| CR1L | 100138386 | complement C3b/C4b receptor 1 like | 1.34 | 0.000328 | 0.017888 |
| HSPH1 | 507165 | heat shock protein family H (Hsp110) member 1 | 1.34 | 6.52E-06 | 0.003433 |
| PTGS2 | 282023 | prostaglandin-endoperoxide synthase 2 | 1.37 | 0.000434 | 0.020428 |
| LOC112446424 | 112446424 | uncharacterized LOC112446424 | 1.42 | 5.32E-06 | 0.003366 |
| CDH10 | 541259 | cadherin 10 | 1.45 | 0.000487 | 0.021545 |
| LOC112444184 | 112444184 | uncharacterized LOC112444184 | 1.47 | 0.001082 | 0.029051 |
| LOC132345128 | 132345128 | uncharacterized LOC132345128 | 1.53 | 0.002855 | 0.044586 |
| LOC132346579 | 132346579 | uncharacterized LOC132346579 | 1.59 | 0.001831 | 0.03657 |
| LOC112449452 | 112449452 | Small nucleolar RNA SNORA70 | 1.62 | 5.73E-05 | 0.008663 |
| CUPIN1 | 112441476 | cupin superfamily member 1 | 1.62 | 0.002882 | 0.044702 |
| LOC132342190 | 132342190 | craniofacial development protein 2-like | 1.69 | 0.002845 | 0.044539 |
| LOC112441687 | 112441687 | U6 spliceosomal RNA | 1.80 | 0.000101 | 0.010317 |
| LOC132346101 | 132346101 | uncharacterized LOC132346101 | 1.80 | 0.003312 | 0.047686 |
| LOC132346013 | 132346013 | uncharacterized LOC132346013 | 1.89 | 0.00142 | 0.032903 |
| TNFAIP8L3 | 523131 | TNF alpha induced protein 8 like 3 | 2.07 | 0.001527 | 0.033384 |
| LOC132345742 | 132345742 | uncharacterized LOC132345742 | 2.22 | 0.002198 | 0.039895 |
| MIR3064 | 104796064 | bta-mir-3064 | 2.40 | 5.35E-05 | 0.008378 |
| KLRJ1 | 444861 | killer cell lectin-like receptor family J member 1 | 2.45 | 0.002936 | 0.045086 |
| TTLL10 | 100336825 | tubulin tyrosine ligase like 10 | 2.90 | 0.002773 | 0.044198 |
| LOC112444896 | 112444896 | uncharacterized LOC112444896 | 3.28 | 0.000869 | 0.026803 |
