# Supplementary material for: Transcriptome changes in leukocytes of dairy calves exposed to heat stress
Source: Transl Anim Sci. 2026 Mar 15;10:txag029. doi: 10.1093/tas/txag029 (PMC13152581; doi:10.1093/tas/txag029)
Supplement: txag029_Supplementary_Data [file txag029_supplementary_data.zip › Additional Table 4.docx]

**Additional Table 4**

| **Category** | **Term** | **Count** | **%** | **PValue** | **Genes** | **Fold Enrichment** | **FDR** |
| --- | --- | --- | --- | --- | --- | --- | --- |
| GOTERM_BP_DIRECT | GO:0031507~heterochromatin formation | 17 | 1.503094607 | 1.36E-08 | 524808, 518504, 783767, 538911, 104975684, 614970, 617975, 616611, 618489, 788724, 404183, 616634, 614974, 618824, 104975683, 529277, 104968446 | 5.988420885 | 3.42E-05 |
| GOTERM_CC_DIRECT | GO:0005634~nucleus | 320 | 28.29354553 | 2.15E-20 | 536561, 509060, 513446, 512477, 286764, 132342086, 540024, 538504, 338054, 510299, 535479, 788459, 533971, 614027, 522836, 112445076, 614936, 613968, 506235, 615907, 509620, 132342072, 540134, 540816, 614014, 767866, 445543, 132342190, 786726, 100140434, 532887, 509613, 768043, 505131, 519409, 521083, 510194, 539257, 514550, 510078, 510991, 615572, 517263, 615211, 618606, 281605, 767858, 767865, 787397, 511959, 504634, 505169, 525895, 539924, 506130, 517139, 536793, 532076, 613941, 787287, 281831, 506127, 786510, 100140210, 514418, 369019, 515988, 616090, 788250, 514579, 515662, 514215, 513125, 518931, 530023, 617854, 617975, 353245, 512392, 618824, 100298738, 528862, 101903505, 100137737, 282875, 281542, 613808, 506398, 281420, 513911, 280691, 529277, 100847190, 517837, 540194, 516866, 514326, 536810, 767826, 534751, 100300568, 511292, 616634, 523615, 282869, 616627, 527301, 526694, 789006, 533414, 505177, 504880, 100299712, 522091, 783820, 100848970, 613596, 618489, 100140915, 786537, 504548, 615527, 530233, 504796, 520684, 783396, 506970, 506294, 540172, 524808, 537566, 540959, 522863, 616611, 532550, 788724, 505749, 511194, 281638, 614557, 768226, 503577, 505756, 786670, 525106, 538091, 515105, 539674, 529071, 534781, 517886, 614783, 286803, 515462, 768207, 539552, 504336, 506999, 107131750, 534413, 507165, 510569, 530736, 513277, 538911, 613560, 615069, 767906, 615863, 617248, 534551, 613566, 517192, 530744, 326581, 132342112, 510557, 509212, 515208, 104968446, 615171, 510474, 540760, 510355, 618441, 540641, 497012, 786336, 100848895, 615059, 616701, 540769, 507619, 282419, 617905, 504599, 768313, 529615, 525496, 100139739, 616819, 527679, 787675, 505564, 115945168, 512888, 115945169, 115945166, 115945167, 115945165, 619094, 540095, 404132, 540632, 534770, 540873, 513971, 518622, 100124429, 100124428, 102216273, 518186, 617109, 782669, 115945171, 112442192, 115945172, 112442193, 520250, 531931, 115945170, 508942, 506402, 541054, 508021, 789062, 508141, 512792, 783767, 540223, 523204, 616800, 504925, 613777, 618428, 524543, 506676, 789996, 506433, 530773, 535306, 515933, 539124, 535321, 615021, 615263, 540332, 540212, 781353, 534599, 522248, 614970, 540339, 518085, 614974, 513195, 787581, 613636, 527388, 534228, 282469, 509139, 525522, 530784, 513626, 535436, 540582, 782690, 513782, 338071, 538489, 540564, 104975684, 614288, 515042, 614047, 538483, 616901, 508508, 280955, 280710, 104975683, 527470, 537706, 508197, 510825, 515957, 527471, 512562, 511234, 519513, 614152, 515399, 614275, 516489, 534576, 617788, 514062, 617428, 100296502, 614948, 100140532, 100137803, 530401, 533115, 790881, 507895, 511226, 509275, 511901 | 1.58819766 | 4.01E-18 |
| GOTERM_CC_DIRECT | GO:0005737~cytoplasm | 241 | 21.30857648 | 1.17E-05 | 539159, 536561, 541597, 512354, 338054, 510299, 788459, 112445076, 523131, 789674, 614936, 506235, 615907, 509620, 539825, 281063, 282032, 786966, 445543, 786726, 532887, 509613, 511007, 541230, 281296, 282023, 515401, 510991, 615211, 522810, 538164, 618606, 525894, 782821, 516289, 539023, 532076, 533836, 281831, 506127, 369019, 539938, 515988, 616090, 505390, 515749, 100848273, 404122, 514215, 518136, 513125, 518931, 539634, 516077, 353245, 100298738, 281306, 282514, 529835, 528862, 282875, 504411, 281542, 281420, 507484, 782162, 507120, 517953, 536810, 534751, 618379, 100300568, 536490, 615424, 617723, 530270, 786096, 282869, 512817, 786890, 613916, 404108, 513228, 511605, 509771, 104968404, 513023, 781884, 100848970, 515444, 540728, 530121, 615773, 613475, 100299845, 506294, 540172, 508591, 534718, 514586, 100337278, 537566, 541246, 540959, 616974, 532550, 513493, 520327, 281638, 614557, 768226, 526430, 507932, 525106, 505079, 789148, 534965, 506163, 537017, 615198, 614783, 515462, 615999, 504336, 528784, 534413, 507165, 530736, 530858, 513277, 515212, 613560, 521237, 767906, 519014, 534551, 504318, 505406, 504565, 326581, 520277, 132342112, 505650, 281453, 510557, 509212, 541077, 506182, 511889, 615171, 510474, 516333, 618441, 540641, 615059, 509803, 539770, 768313, 525011, 525496, 527679, 281202, 505564, 509246, 514822, 327664, 540876, 534770, 282090, 516323, 540873, 407767, 518622, 519276, 534652, 102216273, 505437, 520250, 508942, 506402, 541054, 508021, 789062, 508291, 618785, 540223, 509821, 504925, 539472, 618428, 100138545, 531866, 524543, 506676, 506433, 510246, 515933, 510924, 539124, 535321, 510361, 781353, 541302, 617688, 281816, 613636, 282469, 534106, 509139, 504482, 509282, 338071, 517345, 513785, 540564, 614288, 532021, 539332, 788561, 280955, 280710, 784089, 768070, 537706, 536619, 530516, 515715, 527471, 781376, 511234, 519513, 614152, 614275, 516489, 534576, 618753, 515395, 281478, 530401, 534567, 514858 | 1.282705471 | 0.001312892 |
| GOTERM_CC_DIRECT | GO:0005829~cytosol | 219 | 19.36339523 | 4.33E-18 | 132342081, 539951, 536561, 286764, 541597, 338054, 539274, 523131, 789674, 613968, 522960, 507443, 101906089, 540134, 535248, 525558, 786966, 534032, 445543, 532887, 509613, 281179, 132342078, 281296, 541232, 505131, 511009, 519409, 535916, 512373, 510751, 510991, 615572, 526616, 614002, 517263, 767984, 504634, 509769, 539924, 506130, 536793, 516289, 539701, 505949, 616776, 519310, 522824, 614236, 539023, 616779, 533836, 281831, 100140210, 509631, 515749, 514215, 518136, 518931, 530023, 516077, 353245, 112447338, 281427, 527410, 282514, 528862, 100137737, 282875, 504411, 281542, 533764, 522521, 281420, 507000, 505183, 784903, 132344210, 536810, 100847750, 534751, 523615, 282869, 508566, 404108, 511605, 512056, 516411, 615773, 616740, 100337282, 530233, 509566, 506970, 528288, 506294, 615091, 514586, 286870, 521533, 617268, 513493, 520327, 281638, 768226, 505756, 526430, 507932, 617937, 525106, 504782, 505079, 789148, 508464, 534965, 281990, 529071, 104968456, 616164, 614783, 515462, 506999, 790815, 507165, 530858, 617485, 521918, 521237, 767906, 534551, 504318, 520270, 520277, 507035, 505650, 541077, 510679, 524075, 511889, 510474, 615054, 540881, 510352, 510355, 616701, 282419, 514150, 520120, 789854, 506411, 787675, 513857, 512888, 404132, 507292, 540876, 282090, 518622, 515593, 100124428, 112442192, 531017, 282883, 511665, 513601, 507296, 508021, 508141, 508291, 518659, 100139074, 616120, 540223, 782793, 539472, 618428, 100138545, 525512, 506676, 506433, 515933, 510129, 535321, 540212, 540339, 504914, 522371, 506306, 787465, 614729, 282469, 509139, 505690, 338071, 514991, 515723, 517345, 540564, 404183, 280955, 280710, 613747, 506578, 511919, 506451, 768070, 508197, 515715, 534576, 513091, 618753, 788207, 507409, 539220, 100137803, 534327, 530401, 514858, 507095 | 1.761598859 | 6.06E-16 |
| GOTERM_CC_DIRECT | GO:0005654~nucleoplasm | 178 | 15.7382847 | 5.45E-21 | 515105, 104968456, 509060, 517886, 535479, 788459, 614783, 533971, 286803, 539274, 613336, 506999, 523131, 614936, 613968, 532995, 506235, 522960, 509620, 507165, 521918, 515299, 521237, 614014, 767906, 615863, 525558, 617248, 505406, 517192, 532887, 505650, 510557, 505131, 524590, 524075, 511889, 519409, 535916, 539257, 100848134, 510474, 540881, 526616, 615211, 614002, 615059, 616701, 540769, 507619, 514150, 539770, 617905, 768313, 507107, 504634, 505169, 520120, 527679, 787675, 505564, 115945168, 512888, 115945166, 506130, 507292, 517811, 540632, 534770, 511037, 515476, 539701, 518622, 616776, 505949, 100124428, 102216273, 532076, 112442192, 115945172, 533836, 112442193, 520250, 115945170, 282641, 511665, 513601, 507296, 508021, 616090, 789062, 508141, 518659, 514579, 518136, 513125, 515147, 782793, 530023, 516199, 512392, 509947, 618428, 112447338, 100298738, 100138545, 506676, 525512, 282875, 530773, 281420, 280691, 507000, 505183, 515933, 507120, 517953, 514326, 618375, 515014, 614730, 511292, 504914, 786096, 506306, 282869, 616627, 527301, 527388, 787465, 282469, 613916, 527304, 509139, 508566, 513626, 505177, 508442, 509771, 505293, 515723, 540564, 533235, 616740, 518795, 518274, 789410, 613747, 530233, 506451, 510824, 527471, 615091, 538498, 511234, 519513, 515399, 618232, 516489, 617268, 618753, 513091, 532550, 520327, 511194, 281638, 100296502, 507932, 617937, 525106, 100137803, 504782, 530401, 505079, 790881, 538091, 281990, 507095 | 2.048472029 | 1.52E-18 |
| GOTERM_CC_DIRECT | GO:0000786~nucleosome | 53 | 4.686118479 | 6.12E-23 | 783767, 104968456, 617854, 617975, 616800, 618824, 107131750, 525512, 522960, 530773, 529277, 280691, 505183, 538911, 614970, 616634, 614974, 506306, 616627, 787581, 527388, 787465, 527304, 104968446, 510194, 104975684, 618489, 617905, 504599, 520120, 616819, 104975683, 115945168, 115945169, 115945166, 115945167, 115945165, 515957, 615091, 619094, 524808, 517139, 513971, 616611, 616776, 788724, 115945171, 115945172, 520250, 115945170, 509275, 788250, 511901 | 5.283821568 | 3.42E-20 |
| GOTERM_CC_DIRECT | GO:0000785~chromatin | 41 | 3.625110522 | 4.47E-05 | 783767, 286764, 104975684, 616740, 618489, 540769, 618824, 508508, 100848816, 505169, 104975683, 281542, 529277, 506294, 510825, 515957, 527471, 524808, 540632, 518504, 534770, 515399, 618375, 538911, 516489, 615069, 540816, 522863, 614970, 616611, 788724, 616634, 614974, 514062, 506402, 530401, 513626, 782690, 104968446, 508141, 519409 | 2.005754529 | 0.004161455 |
| GOTERM_CC_DIRECT | GO:0016604~nuclear body | 23 | 2.033598585 | 4.98E-04 | 507193, 540876, 529071, 513446, 540265, 286764, 287327, 522863, 617975, 618606, 513195, 522371, 767865, 525895, 504796, 530784, 535436, 527470, 369019, 404108, 782690, 510825, 506152 | 2.292979171 | 0.03979153 |
| GOTERM_MF_DIRECT | GO:0046872~metal ion binding | 120 | 10.61007958 | 2.60E-05 | 515105, 529071, 537017, 512477, 538504, 615198, 539552, 522836, 505144, 282394, 132342072, 534038, 540816, 613442, 615863, 505406, 100140434, 282023, 510679, 535916, 515401, 539257, 512373, 510078, 539417, 618441, 533312, 615211, 100848895, 616701, 540769, 539770, 100848816, 511959, 525496, 527679, 508039, 504993, 509246, 511836, 404132, 517811, 516289, 517259, 519276, 100124429, 518186, 112442192, 112442193, 787287, 508942, 506402, 541295, 514418, 282366, 511665, 513601, 782793, 523204, 100301148, 281427, 528862, 789996, 506674, 281542, 533764, 515933, 540335, 407109, 781353, 534599, 767826, 280969, 527301, 789006, 515809, 614729, 525522, 281133, 505177, 508442, 100299712, 522091, 782050, 509282, 783820, 517345, 516498, 515444, 523424, 521401, 613475, 100140915, 280710, 281767, 520684, 529410, 100337204, 768070, 510825, 515715, 519513, 614275, 618753, 505749, 513493, 511194, 518384, 768226, 525344, 100296502, 281874, 100125390, 525106, 790881, 768063, 508586, 781260, 281990, 509032 | 1.45140736 | 0.006262262 |
| GOTERM_MF_DIRECT | GO:0003677~DNA binding | 114 | 10.0795756 | 1.53E-14 | 104968456, 513446, 286764, 538504, 522836, 107131750, 614936, 506235, 522960, 538911, 540816, 614014, 525558, 617248, 613566, 517192, 531436, 768043, 104968446, 535916, 510194, 513065, 510078, 497012, 615211, 100848895, 618606, 540769, 617905, 504599, 768313, 511959, 100139739, 616819, 520120, 789854, 787675, 115945168, 115945169, 539924, 115945166, 115945167, 115945165, 619094, 540095, 518504, 517139, 534770, 515476, 616776, 100124429, 518186, 407241, 115945171, 112442192, 115945172, 112442193, 115945170, 506402, 515988, 616090, 788250, 783767, 515662, 617854, 616800, 100301148, 618824, 101903505, 525512, 789996, 281542, 530773, 529277, 280691, 505183, 615021, 614970, 767826, 614974, 513195, 616634, 506306, 787581, 616627, 527388, 789006, 787465, 527304, 509259, 525522, 513626, 782690, 522091, 505293, 538489, 104975684, 618489, 538483, 100140915, 616901, 789410, 104975683, 520684, 506294, 515957, 615091, 524808, 616611, 788724, 511194, 100296502, 530401, 281990 | 2.139298613 | 4.91E-12 |
| GOTERM_MF_DIRECT | GO:0042802~identical protein binding | 69 | 6.100795756 | 4.45E-05 | 404122, 515105, 539674, 104968456, 512477, 540265, 788459, 526639, 353245, 539791, 281427, 529835, 613649, 281542, 506235, 517837, 505183, 530858, 539645, 536810, 617688, 518288, 618457, 280969, 786966, 767866, 505649, 506306, 787465, 534228, 281210, 510557, 510679, 506182, 540881, 512299, 615572, 783068, 614320, 506928, 615059, 404183, 281849, 618606, 280955, 614957, 615408, 505169, 506413, 509566, 512888, 509246, 507462, 515715, 404132, 540632, 512562, 540954, 517259, 539701, 540873, 522863, 616776, 532671, 505749, 537241, 506127, 509631, 515988 | 1.658259886 | 0.008586293 |
| GOTERM_MF_DIRECT | GO:0046982~protein heterodimerization activity | 59 | 5.216622458 | 4.99E-16 | 783767, 508291, 104968456, 616800, 618824, 107131750, 282514, 525512, 506674, 281542, 522960, 530773, 506235, 529277, 280691, 505183, 538911, 614970, 540339, 513195, 616634, 614974, 506306, 616627, 787581, 527388, 787465, 104968446, 513065, 104975684, 506928, 618489, 617905, 504599, 507107, 509769, 520120, 616819, 789854, 104975683, 115945168, 115945169, 115945166, 115945167, 115945165, 615091, 619094, 524808, 517139, 536793, 616611, 616776, 788724, 115945171, 115945172, 115945170, 539938, 539616, 788250 | 3.413188426 | 2.41E-13 |
| GOTERM_MF_DIRECT | GO:0030527~structural constituent of chromatin | 52 | 4.597701149 | 8.61E-25 | 783767, 104968456, 617854, 617975, 616800, 618824, 107131750, 525512, 522960, 530773, 529277, 280691, 505183, 538911, 614970, 616634, 614974, 506306, 616627, 787581, 527388, 787465, 527304, 104968446, 510194, 104975684, 618489, 617905, 504599, 520120, 616819, 104975683, 115945168, 115945169, 115945166, 115945167, 115945165, 515957, 615091, 619094, 524808, 517139, 513971, 616611, 616776, 788724, 115945171, 115945172, 115945170, 509275, 788250, 511901 | 5.871967417 | 8.30E-22 |
| INTERPRO | IPR009072:Histone-fold | 49 | 4.33244916 | 2.19E-22 | 783767, 104968456, 616800, 618824, 107131750, 525512, 506674, 522960, 530773, 529277, 280691, 505183, 538911, 614970, 513195, 616634, 614974, 506306, 616627, 787581, 527388, 787465, 104968446, 513065, 104975684, 618489, 617905, 504599, 520120, 616819, 789854, 104975683, 115945168, 115945169, 115945166, 115945167, 115945165, 615091, 619094, 524808, 517139, 616611, 616776, 788724, 115945171, 115945172, 115945170, 539938, 788250 | 5.622196911 | 5.28E-19 |
| INTERPRO | IPR013087:Znf_C2H2_type | 48 | 4.24403183 | 1.11E-04 | 538489, 783820, 512477, 510078, 132342086, 538504, 523204, 100848895, 100140915, 512392, 616701, 507619, 786537, 100301148, 539552, 522836, 511959, 789996, 787675, 522521, 520684, 506398, 509462, 615021, 540816, 767826, 614014, 100124429, 525558, 505749, 511194, 613566, 407241, 112442192, 527301, 112442193, 100296502, 787287, 526694, 789006, 100140434, 100137803, 525522, 790881, 514418, 505177, 100299712, 789062 | 1.812678819 | 0.019076039 |
| INTERPRO | IPR036236:Znf_C2H2_sf | 46 | 4.067197171 | 1.41E-04 | 538489, 783820, 512477, 510078, 132342086, 538504, 523204, 100848895, 100140915, 512392, 616701, 507619, 786537, 100301148, 539552, 522836, 511959, 789996, 787675, 520684, 506398, 509462, 615021, 540816, 767826, 614014, 100124429, 525558, 511194, 613566, 407241, 112442192, 527301, 112442193, 100296502, 787287, 526694, 789006, 100140434, 100137803, 525522, 790881, 514418, 100299712, 513601, 789062 | 1.821329636 | 0.022656812 |
| INTERPRO | IPR007125:Histone_H2A/H2B/H3 | 35 | 3.094606543 | 2.20E-16 | 783767, 104968456, 104975684, 618489, 616800, 618824, 107131750, 504599, 525512, 616819, 520120, 104975683, 522960, 115945169, 529277, 115945167, 505183, 115945165, 615091, 524808, 517139, 538911, 614970, 616776, 616611, 788724, 616634, 614974, 506306, 115945171, 787581, 616627, 787465, 104968446, 788250 | 5.71955097 | 2.66E-13 |
| INTERPRO | IPR000980:SH2 | 14 | 1.237842617 | 3.12E-04 | 539951, 514773, 100848273, 540876, 617485, 518795, 526430, 504782, 532587, 509246, 507296, 515988, 282032, 512726 | 3.311318983 | 0.047102848 |
| INTERPRO | IPR002119:Histone_H2A | 14 | 1.237842617 | 3.56E-10 | 524808, 783767, 538911, 104975684, 614970, 616611, 618489, 788724, 616634, 614974, 618824, 104975683, 529277, 104968446 | 10.20244227 | 1.72E-07 |
| INTERPRO | IPR032454:Histone_H2A_C | 14 | 1.237842617 | 4.12E-11 | 524808, 783767, 538911, 104975684, 614970, 616611, 618489, 788724, 616634, 614974, 618824, 104975683, 529277, 104968446 | 11.79657388 | 2.49E-08 |
| INTERPRO | IPR032458:Histone_H2A_CS | 14 | 1.237842617 | 1.52E-11 | 524808, 783767, 538911, 104975684, 614970, 616611, 618489, 788724, 616634, 614974, 618824, 104975683, 529277, 104968446 | 12.58301213 | 1.23E-08 |
| INTERPRO | IPR055333:HISTONE_H2B_site | 11 | 0.972590628 | 6.91E-06 | 506306, 787581, 616627, 104968456, 525512, 787465, 520120, 522960, 616776, 505183, 615091 | 6.310629186 | 0.001390516 |
| INTERPRO | IPR000164:Histone_H3/CENP-A | 10 | 0.884173298 | 4.83E-06 | 107131750, 115945171, 504599, 517139, 616819, 115945169, 115945167, 616800, 115945165, 788250 | 7.489888175 | 0.001167184 |
| INTERPRO | IPR035425:CENP-T/H4_C | 10 | 0.884173298 | 4.63E-07 | 617905, 115945172, 527388, 115945170, 530773, 115945168, 115945166, 280691, 513195, 619094 | 9.629856225 | 1.42E-04 |
| INTERPRO | IPR001951:Histone_H4 | 9 | 0.795755968 | 9.75E-06 | 617905, 115945172, 527388, 115945170, 530773, 115945168, 115945166, 280691, 619094 | 8.089079229 | 0.001811052 |
| INTERPRO | IPR004823:TAF_TATA-bd_Histone-like_dom | 9 | 0.795755968 | 2.97E-06 | 617905, 115945172, 527388, 115945170, 530773, 115945168, 115945166, 280691, 619094 | 9.333552957 | 7.97E-04 |
| INTERPRO | IPR019809:Histone_H4_CS | 9 | 0.795755968 | 5.53E-06 | 617905, 115945172, 527388, 115945170, 530773, 115945168, 115945166, 280691, 619094 | 8.666870603 | 0.001213944 |
| INTERPRO | IPR005818:Histone_H1/H5_H15 | 8 | 0.707338638 | 4.64E-07 | 510194, 527304, 513971, 617854, 617975, 509275, 515957, 511901 | 14.3805853 | 1.42E-04 |
| INTERPRO | IPR005819:H1/H5 | 7 | 0.618921309 | 4.70E-07 | 527304, 513971, 617854, 617975, 509275, 515957, 511901 | 18.8745182 | 1.42E-04 |
| KEGG_PATHWAY | bta03040:Spliceosome | 44 | 3.890362511 | 1.99E-11 | 112442846, 513782, 112443636, 112443614, 112442845, 510474, 112442807, 112441916, 112446509, 615198, 112448627, 112442784, 112446125, 112442843, 507944, 112447196, 512327, 529231, 537706, 112442857, 112442855, 112442856, 112448638, 101902301, 112442110, 112442111, 112449629, 100124428, 112443342, 112443327, 112442853, 112442854, 782669, 112442852, 786726, 617519, 531931, 534228, 281831, 112446130, 112446131, 107132278, 507895, 112446153 | 3.178661754 | 2.11E-09 |
| KEGG_PATHWAY | bta04613:Neutrophil extracellular trap formation | 40 | 3.536693192 | 5.20E-12 | 404122, 783767, 104968456, 104975684, 618489, 616800, 618824, 100138386, 107131750, 617905, 504599, 525512, 616819, 520120, 104975683, 522960, 530773, 529277, 280691, 505183, 619094, 615091, 524808, 517139, 538911, 614970, 616776, 616611, 788724, 616634, 614974, 506306, 787581, 616627, 527388, 787465, 281874, 508888, 104968446, 788250 | 3.570322557 | 8.24E-10 |
| KEGG_PATHWAY | bta05034:Alcoholism | 38 | 3.359858532 | 2.94E-11 | 783767, 104968456, 104975684, 618489, 616800, 618824, 107131750, 617905, 504599, 525512, 616819, 520120, 104975683, 522960, 530773, 281202, 529277, 280691, 505183, 619094, 615091, 524808, 517139, 538911, 614970, 616776, 616611, 788724, 616634, 614974, 506306, 787581, 616627, 527388, 787465, 520277, 104968446, 788250 | 3.522820411 | 2.33E-09 |
| KEGG_PATHWAY | bta05322:Systemic lupus erythematosus | 38 | 3.359858532 | 4.66E-14 | 783767, 104968456, 338071, 104975684, 618489, 616800, 286849, 618824, 107131750, 617905, 504599, 525512, 616819, 520120, 104975683, 522960, 530773, 529277, 280691, 505183, 619094, 615091, 524808, 517139, 538911, 614970, 616776, 616611, 788724, 616634, 614974, 506306, 787581, 616627, 527388, 787465, 104968446, 788250 | 4.320090293 | 1.48E-11 |
| KEGG_PATHWAY | bta03082:ATP-dependent chromatin remodeling | 23 | 2.033598585 | 3.74E-08 | 524808, 404122, 783767, 509060, 538911, 104975684, 614970, 616611, 618489, 613596, 788724, 768207, 616634, 614974, 518186, 618824, 533426, 506402, 104975683, 529277, 508442, 616090, 104968446 | 4.039108811 | 2.37E-06 |
