# Supplementary material for: Transcriptome changes in leukocytes of dairy calves exposed to heat stress
Source: Transl Anim Sci. 2026 Mar 15;10:txag029. doi: 10.1093/tas/txag029 (PMC13152581; doi:10.1093/tas/txag029)
Supplement: txag029_Supplementary_Data [file txag029_supplementary_data.zip › Additional Table 6.docx]

**Additional Table 6.** Differentially expressed genes in the “Neutrophils extracellular trap formation” KEGG pathways in white blood cells of heat-stressed dairy calves.
