# Supplementary figures and images for: Transcriptome changes in leukocytes of dairy calves exposed to heat stress
Source: Transl Anim Sci. 2026 Mar 15;10:txag029. doi: 10.1093/tas/txag029 (PMC13152581; doi:10.1093/tas/txag029)

**Additional Table 5**


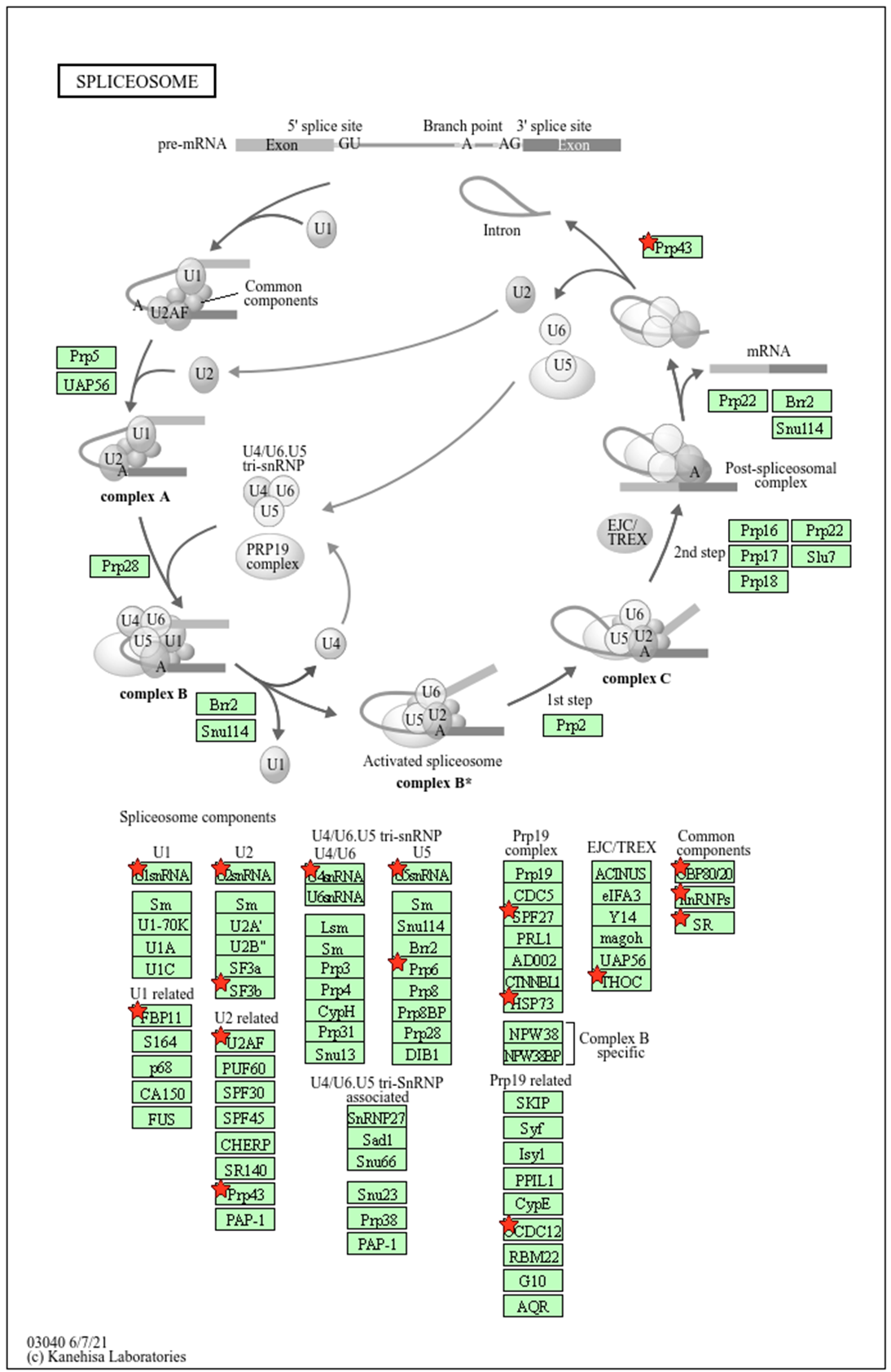

Supplement: txag029_Supplementary_Data [file txag029_supplementary_data.zip › Additional Table 5.docx]
